# Detecting Important Features and Predicting Yield from Defects Detected by SEM in Semiconductor Production

**DOI:** 10.3390/s25134218

**Published:** 2025-07-06

**Authors:** Umberto Amato, Anestis Antoniadis, Italia De Feis, Anastasiia Doinychko, Irène Gijbels, Antonino La Magna, Daniele Pagano, Francesco Piccinini, Easter Selvan Suviseshamuthu, Carlo Severgnini, Andres Torres, Patrizia Vasquez

**Affiliations:** 1 Istituto di Scienze Applicate e Sistemi Intelligenti, National Research Council of Italy, 80131 Napoli, Italy; antoniadis.anestis@na.isasi.cnr.it; 2 Istituto per le Applicazioni del Calcolo ‘Mauro Picone’, National Research Council of Italy, 80131 Napoli, Italy; i.defeis@na.iac.cnr.it; 3 Siemens EDA, Plano, TX 75024, USA; anastasiia.doinychko@siemens.com (A.D.); andres.torres@siemens.com (A.T.); 4 Department of Mathematics, KU Leuven, 3001 Leuven, Belgium; irene.gijbels@kuleuven.be; 5 Istituto per la Microelettronica e Microsistemi, National Research Council of Italy, 95121 Catania, Italy; antonino.lamagna@imm.cnr.it; 6 STMicroelectronics, 95121 Catania, Italy; daniele.pagano@st.com (D.P.); patriziavasquez59@gmail.com (P.V.); 7 STMicroelectronics, 20864 Agrate Brianza, Italy; francesco.piccinini@st.com (F.P.); carlo.severgnini@st.com (C.S.); 8 Kessler Foundation, East Hanover, NJ 07936, USA; eselvan@kesslerfoundation.org

**Keywords:** semiconductors, yield, Scanning Electron Microscope, predictive maintenance, Gradient Boosting, Odds Ratio, 82.45.VP, 62P30

## Abstract

A key step to optimize the tests of semiconductors during the production process is to improve the prediction of the final yield from the defects detected on the wafers during the production process. This study investigates the link between the defects detected by a Scanning Electron Microscope (SEM) and the electrical failure of the final semiconductors, with two main objectives: (a) to identify the best layers to inspect by SEM; (b) to develop a model that predicts electrical failures of the semiconductors from the detected defects. The first objective has been reached by a model based on Odds Ratio that gave a (ranked) list of the layers that best predict the final yield. This allows process engineers to concentrate inspections on a few important layers. For the second objective, a regression/classification model based on Gradient Boosting has been developed. As a by-product, this latter model confirmed the results obtained by Odds Ratio analysis. Both models take account of the high lacunarity of the data and have been validated on two distinct datasets from STMicroelectronics.

## 1. Introduction

### 1.1. Background of the Study

The semiconductor industry accounted for USD 627 billion in sales in 2024, with an increase of 19% year-to-year and also a prospective double-digit increase for the current year (∼ 11% ) [1]. It is estimated that sales could reach USD 1 trillion by 2030 and 2 trillion by 2040. Even though other industries show a higher market size (about USD 3 trillion for automotive, almost USD 2 trillion for pharmaceutical, about USD 1 trillion for consumer electronics, about USD 2 trillion for the energy transition market), the semiconductor industry assumes a strategic role since it is a key enabling technology for other industries (primarily, automotive and consumer electronics) and is a very high value-add (an inexpensive chip can greatly increase the value of an end product). Moreover, it has a growing geopolitical role for maintaining national power and security (consider, for example, the recent US and EU Chips Acts).

Therefore, it is imperative for industries to reduce production costs in order to maintain competitiveness in an aggressive and rapidly changing technological world, while ensuring that the high cost of R&D is met (presently about 50% of profits, excluding interest and taxes [1]).

While models exist to optimize the entire semiconductor production process [2], yield is a crucial indicator for the performance and reliability of the fabrication facility, and its increase, together with the mitigation of the risk of equipment failure, are key objectives of the semiconductor industry. It is estimated that a 1% increase in yield produces about USD 150M in profit [3].

### 1.2. Problem Statement and Research Objectives

In semiconductor manufacturing, the production of integrated circuits follows a sequential flow that involves hundreds of ordered elementary operations (layers), e.g., polishing, material deposition, modification, metallization, lithography, etching, and more. Upon completion of this fabrication process, a wafer with a circular shape emerges, hosting a certain number of dice, typically around 1000. Then, individual dice on the wafer undergo electrical testing to identify and discard those that do not meet the specified functionality requirements. The yield of the production process is determined by the rate of dice that successfully pass the final electrical tests.

In the absence of any control, all wafers undergo the complete set of extensive operations scheduled by the manufacturing process. However, when a wafer eventually exhibits a substantial number of dice with electrical failures, it results in a waste of time and incurs economic costs. To mitigate this, various checks are strategically integrated into the process flow at certain layers to detect defects occurring on the wafers. They comprise two distinct stages. The initial stage results from an automated preliminary analysis conducted on a wafer at the conclusion of the operation, corresponding to the specific layer. This analysis provides an estimation based solely on the presence of defects on each die within the wafer. Subsequently, a more thorough inspection can be conducted using a Scanning Electron Microscope (SEM) SemVision G3 by Applied Materials Inc., Santa Clara, CA, USA, equipped with Artificial Intelligence (AI) software designed to process the resultant images [4]. In essence, the entire wafer undergoes a comprehensive scan, and defects are potentially identified at various positions on the wafer (and consequently, on the individual dice) through the AI software. The software also possesses the capability to categorize defects into predetermined types, such as scratches, impurities, residues, and specific patterns. Hence, defects are assigned numerical codes corresponding to their specific types during the AI processing. The code “0" is reserved for generic defects found during the initial step of defect detection. It is worth noting that, as we will explore later, the majority of dice undergo investigation only for generic defects. Importantly, the presence of a defect on a dice does not automatically imply that an issue is prone to electrical failures.

This study has two primary objectives:

(a) to find the layers (and types of defect) mostly associated with failures of dice;
(b) to develop a predictive model of the electrical failures (and consequently of the yield of production) starting from the defects detected.

These objectives allow process engineers to focus on the specific detection of defects by SEM at the layers with the highest association with electrical failures, thereby reducing the number of SEM investigations and ultimately the cost of tests. Moreover, this approach allows for the early identification of issues in the process chain that may reduce the final yield of production, enabling the preemptive rejection of wafers expected to have a low final yield, thus resulting in further cost savings. Finally, identifying the process responsible for the malfunction facilitates targeted maintenance interventions, preventing the involvement of other wafers, and further reducing the cost of production.

## 2. Research Process

To reach the objectives of the study outlined in Section 1.2, the key tool is a predictive model of the outcome of the final electrical test on each die at the end of production, starting from defects detected on the wafers during the production process, and it must also be able to interpret the results and select important features.

Several methodologies are available for the classification engine needed. The primary approach employed in this study is Gradient Boosting (GB) [5], an ensemble-based method for regression and classification tasks. In this technique, many Decision Trees are iteratively and sequentially trained. Each of them is a weak predictor, but each iteration sequentially minimizes the residuals of prediction estimated since then, focusing on cases with the largest error, so that the ensemble of all trees has a high accuracy. See Bentéjac et al. [6] for a recent review of GB and its variants.

The main computational cost of GB lies in constructing Decision Trees, with the most time-consuming part being the search for the best split points for each node. Notably, Decision Tree solvers can automatically handle missing values, a crucial feature for our study.

By their very construction, methods based on Decision Trees are subject to oversmoothing, due to the increasing number of trees built during iterations. The consolidated method for facing this problem is interrupting iterations. We chose the optimal iteration by 10-fold cross validation on the validation subset. In practice, the prediction model is trained on the training subset, and error is estimated separately on the training and validation subsets. While the former decreases with iterations due to oversmoothing, the latter, acting on a subset unseen during training, generally reaches a minimum value in correspondence to the optimal iteration. This 10-fold cross validation procedure has also been used for choosing all the other hyperparameters involved in the GB method.

A nice property of GB is its capability to naturally treat missing data as information, instead of discarding the entire sample, as happens with most competitor methods (e.g., logistic regression, SVM).

In this work, eXtreme Gradient Boosting (XGB) implementation was used [7], offering improvements in computational speed compared to other basic GB-based methods. Since the response is binary, we endowed it with the logistic function, with the output representing the probability that a die is predicted to be faulty. This probability is then converted into a binary variable using a suitable cutoff.

As will be clear in Section 4, and particularly Section 4.3, our study is affected by missing data of layers. This is a consequence of the cost incurred in gathering images through SEM during the production process, which severely limits the number of inspected layers, generating theoretical and practical processing issues.

Several methods are available in the literature to face the problem of missing data (imputation), beginning with the most simplistic, followed by the probabilistic ones, and ending with more elaborated ones [8,9]. Actually, a debate is active on the minimum fraction of missing data for which imputation can take effect (minimum fraction depends on the amount of data; the larger the sample size, the higher the admissible fraction of missing data). Until recently, any guidelines on imputation discouraged the estimation of missing data when their fraction was higher than the rule-of-thumb of 5–10%. For a reference, in Section 4 we show that the fraction of missing data in our study reaches values above 95%. Recent works have proved the benefits of some imputation techniques on specific datasets and for fractions of missing data as high as 80-90% in the frameworks of MCAR (Missing Completely at Random) and MAR (Missing at Random) types of missing data, whereas no results are available to our knowledge for the most difficult case of NMAR (Not Missing at Random). See, e.g., Madley-Dowd et al. [10], Lee and Huber [11], Chen and Savalei [12] for general arguments and [13] for the specific case of semiconductors. For our problem of semiconductors, it seems reasonable that the category of missing data is the most difficult one (NMAR). This is due to the non-random selection of layers subject to inspection for defects through SEM, as will be shown in Section 4.3. See, in this respect, the excellent discussion in [14] for the semiconductor problem. See also Lee et al. [13] for an easier case where all layers are always investigated for a wafer, resulting in a possible MCAR framework. The practical consequence is that neither probabilistic methods can be considered accurate in imputing missing data. Therefore, among the objectives of the present study we can add:

(c) to develop methodologies maximally noncommittal with respect to the distribution of missing data, potentially working without imputation.

We will rely on our chosen engine for classification (GB), considering that it naturally deals with missing data. However, to strengthen our analysis, we develop an alternative and independent methodology for estimating the interesting features. Such a methodology is based on Odds Ratio analysis, commonly employed in case-control studies in medical sciences. This approach allows for a quantitative assessment of the impact of layers and types of defects (herein also generically called factors) on predicting electrical failures.

The Odds Ratio represents the odds that an outcome will occur (i.e., an electrical test fails) given a specific exposure (i.e., defects at a particular layer or of a certain type are detected), compared to the odds of the outcome occurring in the absence of that exposure. Odds Ratio analysis enhances the contrast between the two conditions and, consequently, the two opposing roles of factors with respect to electrical failures (the outcome). It answers the research question “What is the probability of a failure of the electrical test given the exposure to a defect of a certain type or at a certain layer?”

**Figure 1:**
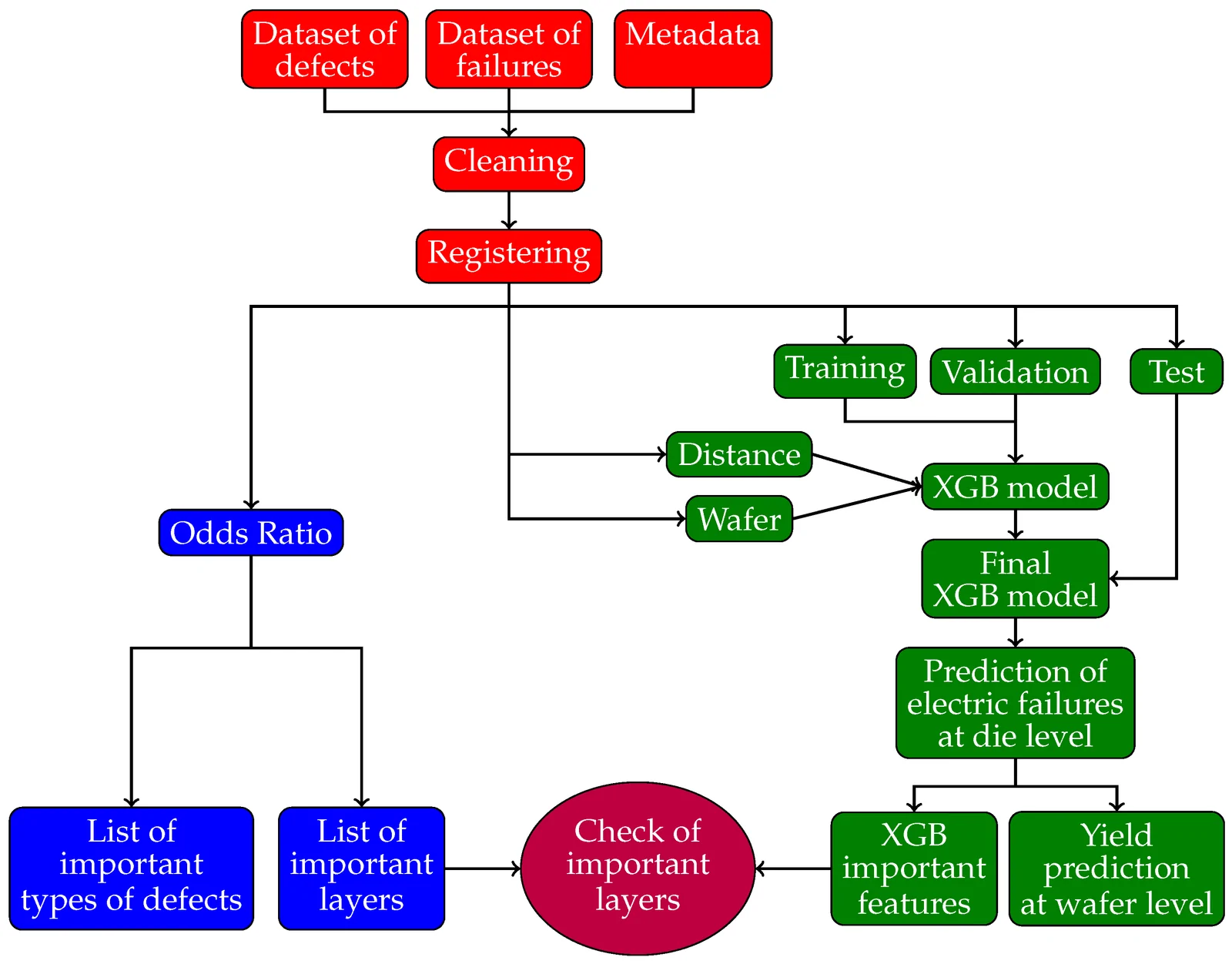
Scheme of the work: data collection and preprocessing (red boxes); Odds Ratio analysis (blue boxes); predictive models of die failure by Gradient Boosting and of wafer yield (green boxes). An ellipsoidal purple box indicates the cross-check of results between Odds Ratio and Gradient Boosting analyses.

The paper is organized as in the scheme of Figure 1. Section 4 (red boxes in Figure 1) describes the datasets supplied by STMicroelectronics, which are the focal point of our analysis, and their preprocessing (cleaning and registration of data). An analysis of layers is included in Section 4.3, particularly relevant to this study. Section 5 (blue boxes in Figure 1) explores the associations between various factors measured throughout the production process and the final failure of the dice by using Odds Ratio analysis. Section 6 (green boxes in Figure 1) is dedicated to the development of the XGB model that predicts the electrical failure of a die. The general framework of the methodology is first introduced (including the preparation of the training, validation, and test subsets and the choice of the predictors). Then, the model for estimating the outcome of the final electrical tests is discussed. Finally, the prediction of yield at the wafer level is obtained, aggregating the results of the prediction model die-by-die. All results are discussed in Section 7. Finally, conclusions are drawn in Section 8.

## 3. Literature Review

There is a rich source of literature on the prediction and characterization of the yield of semiconductor production.

A first class of models investigate maps of electrical failures on the wafers because their type are predictive of the reason of the failures. See, for example, Shukla [15] through a simple probabilistic method, Li et al. [16], using a quite complex procedure involving several statistics and Machine Learning (ML) tools, and Li et al. [17] using the Hough transform. Many recent models are based on CNN and its variants [18,19,20,21,22,23,24], also endowed with Long Short-term Memory (LSTM) for time-series analysis [25]. We also mention alternative Deep Learning tools such as Bidirectional Encoder Representation from Image Transformers [26], and Deep Capsule Network [27].

Another class of methods attempts to predict the yield starting from data gathered during the production. Yu [28] develops a method relying on a Principal Components-Based Gaussian Mixture Model, while Baly and Hajj [29] and Lee et al. [13] use SVM. Stich et al. [30] introduce a general framework based on a cascading prediction algorithm involving all the steps of the fabrication process. Fan et al. [31] propose a model based on an LSTM encoder network analyzing the time series of data, validated on proprietary data from the Seagate company. Lee and Roh [32], basing on proprietary data from Samsung Electronics, introduce eXplainable Artificial Intelligence (XAI) as a framework and test several regression methodologies, also estimating the driving factors of prediction by the Shapley additive explanation (SHAP). Han et al. [33] use XGBoost and Bayesian Optimization to improve imbalanced learning performance, also suggesting key factors for detection diagnosis. Park et al. [34] rely on the SECOM public dataset and also compare a selection of regression methodologies, focusing on reducing the dimension of predictors and balancing data through SMOTE. Jiang et al. [35] focus their attention on introducing factors of many different types (numerical, categorical, etc.) into the models without prior knowledge, making them more friendly. Their experiments are based on proprietary data from Silicon Laboratories. They also provide a feature importance analysis through the Gini importance indicator. Vankayalapati et al. [36] rely on Ada Boost and a Recurrent Neural Network (RNN) model, while the model by Zhai et al. [3] is based on a specific xAutoML model and also includes an estimate of key diagnosis factors.

The advantage of the above models is the availability of massive data that are routinely gathered in real-time during the process. However, they do not generally directly include data on defects detected on the wafers, which need an offline acquisition of images through suitable equipment and their processing to recognize defects. In this respect, some recent works are available that introduce hardware detectors to collect images of wafers endowed with software tools to recognize defects on each die of the wafers. We should mention the system based on SEM and on a modified U-net architecture to recognize and classify defects on which this study relies [4]. We also mention Chu et al. [37], who consider images detected by generic optical systems and propose a domain adaptation method based on adversial learning Deep Learning tools for detecting and classifying defects, aimed at keeping detection accuracy high when changing optical systems.

The availability of such systems has fostered research on methods that specifically consider defects as variables for predicting the electrical failure of dice, and ultimately yield. In their early works, Yeh et al. [38] estimated yield by a simple probabilistic model assuming a random or a clustered distribution of defects recognized from images by experts. Nutsch et al. [39] consider the association between yield and defects on the wafers, giving insights in the decrease of the former with the improvement of the generation technology process (νm) without proposing a direct prediction model. Such a model is instead developed in Kang et al. [40], where predictor variables are the distance of the die from the wafer center, previous final yield at the die position, wafer test pass rate of the adjacent dies, and abnormalities of the wafer map pattern. The methodology relies on Random Forest and works at the die level. No estimation of the features is included. Support Vector Regression (SVR) is used in Kong and Ni [41]. Defects are detected through alternate stripe scanning data collection; therefore, the resulting missing data are imputed by K-Nearest Neighbor (KNN) methodology. Noteworthy is the fact that a random subset of wafers was inspected at all 23 chosen layers in the experiments.

More recently, Lenhard et al. [42] proposed a die-based model that uses both data gathered during the process and defects detected by the KLA-Tencor Altair 8290 patterned wafer inspection system in one of the experiments. The work relies on Feedforward Deep Neural Networks (FNNs) with a non-polynomial activation function. No key features are estimated. Incidentally, Lenhard et al. [42] prove that the use of defect data gathered from the inspection of wafers through dedicated acquisition systems significantly improves the prediction of yield with respect to models using only massive data routinely gathered during the process.

A distinct approach was employed in Baron et al. [43], building on Baron et al. [44] and Baron et al. [45]. The experimental setup is very similar to the present study, as is the main research question—to predict the probability of an electrical failure of each single die starting from defects detected at certain layers. The principal contribution of Baron et al. [43] is the development of a theoretical framework based on Bayesian arguments that estimates the probability of occurrence of missing data from available information, yielding causal relationships between defects and failing dice. The model is detailed enough to include dependence between types of defects and layers and the number of detected defects, again based on Bayesian arguments. Both generic defects and those found after specific inspection are considered in the same model. Moreover, the effect of the lot is also included.

Finally we mention the work by Nurani et al. [46], who introduce a general methodology for optimizing the inspection of wafers aimed at balancing the cost of inspection and the cost of failed devices through a suitable trade-off. Their framework is general and not linked to a specific technology for the inspection of wafers.

Only a few papers predict the outcome of the final electrical tests on a die basis [40,41,42,43]. In addition, significant features are estimated only in Baron et al. [43] (and Jiang et al. [35], who, however, did not develop an operational model). Most importantly, the problem of missing data does not occur in the data or is not as severe as in our study. For example, all layers are subject to inspection in [22]. Even in Baron et al. [43], whose study is the closest to ours in many respects, the data are of a much larger size than our study and are taken at a much higher number of layers (exact data are not disclosed due to commercial confidentiality), with the number of possible layers being also much smaller than our study (11 vs. 30–35). In practice, studies were not taken in severely critical conditions of missing data.

Therefore, our main contribution is to develop methods, as presented in Section 2, able to deal with severely lacunary datasets, where only very few layers are inspected.

## 4. The Dataset

STMicroelectronics has supplied two distinct datasets, designated as ARES and TETIS, corresponding to two different products. Each dataset includes data for a selection of lots, and for each lot a sample of wafers is included, with each wafer drawn from a possible set of 25. The number of dice on each wafer varies depending on the product within the dataset. Certain wafers undergo an inspection of all dice at specific selected layers. On each die, one or more defects may be identified, and in many instances the SEM system equipped with AI software is utilized to estimate the type of defect detected through image processing. It is crucial to highlight that this dataset represents a unique occurrence in the entire production process, where joint information collected during production and the final results of the electrical tests are available on a die-by-die basis.

Table 1 provides a comprehensive summary of the datasets furnished by STMicroelectronics for the purposes of this study.

The two datasets, although related to distinct products, exhibit similarities in several aspects. Both share comparable numbers of lots and wafers. However, the dataset TETIS features larger dice, resulting in a lower overall number of dice. The number of inspected layers is similar, with 30 layers for the dataset ARES and 35 for the dataset TETIS. While the defects inspected by SEM are the same for both datasets, the absence of defects for a few types results in a slight disparity in the number of defect types, with 72 for ARES and 75 for TETIS. Notably, the dataset TETIS displays a higher rate of electrical failures (lower yield), at 9.1% compared to 4.7% for ARES. However, the average number of detected defects per die is similar between the two datasets, with 0.20 for TETIS and 0.24 for ARES. It is noteworthy that the low average number of layers inspected per wafer (2.3–2.4 on average out of the 30–35 available for the two datasets) poses a significant challenge for the statistical methods used, resulting in a high fraction of missing data that reaches values greater than 95%.

Additionally, the fraction of dice with a certified defect (type >0 ) is small, accounting for 4.2% and 5.1% of the inspected dice for the datasets ARES and TETIS, respectively. This figure increases when considering unclassified defects (type =0 ), to 20.5% for the dataset ARES and 14.8% for the dataset TETIS.

From Table 1, all dice in the datasets are investigated to identify generic defects (type =0 ), while only a fraction of the dice are searched by SEM (15.4% and 16.1% for the datasets ARES and TETIS, respectively). When limiting the analysis to dice inspected for specific defects, the failure rate remains similar, at 5.3% for ARES and 9.2% for TETIS (it was 4.7% and 9.1%, respectively, for the entire datasets), indicating that the choice of wafers for SEM analysis is seemingly random. However, when restricting the analysis to dice showing at least one generic defect (type =0 ), the failure rate increases to 6.8% for ARES and 13.2% for TETIS. Further narrowing the analysis to dice investigated by SEM for classified defects (type >0 ) with at least one classified defect detected, the failure rate increases to 16.3% for ARES and 19.0% for TETIS. This represents more than triple and double the average rate of the entire dataset, and more than double and 50% more compared to the case when only generic defects are found. This emphasizes that, while the inspected wafers have a similar failure rate to the entire population, when defects are detected the failure rate significantly increases. This confirms the effectiveness of defect detection in predicting die failure.

This observation carries immediate and significant consequences:

- The detection of defects on a die is associated with a higher probability of failure in the final electrical test. This increase becomes notably substantial, reaching 250% for the dataset ARES and 110% for the dataset TETIS in comparison to the entire population.
- The criteria employed by STMicroelectronics process engineers for selecting layers at which to inspect wafers prove to be effective. This is evident from the resulting significantly higher rate of electrical failures observed.

Table 1 also highlights a notable occurrence—a substantial number of dice experience a failed final electrical test despite having no detected defects. This phenomenon is observed in 87.1% and 89.5% of dice with a failure of the final electrical tests for the datasets ARES and TETIS, respectively. In other words, no defect was identified, even though the final electrical test indicated a failure. It is evident that, lacking additional information, it is challenging to predict the final failure of the electrical test for each individual die based solely on the defects (not) detected at the inspected layers. Moreover, identifying the types of defects and/or layers responsible for the failure is not feasible. However, these cases can still provide indirect insights into the types of defect and/or layers that are least (or not at all) associated with an electrical failure.

Three main possible explanations account for this phenomenon:

1. There are causes contributing to electrical failures that extend beyond those discernible through defects detected by the SEM system and the AI tools.
2. Defects have not been recognized by the AI tools.
3. Defects might not have been inspected at the appropriate layers. Given the low average number of inspected layers for each wafer, as detailed in Table 1, it is plausible that the layers potentially experiencing defects were not adequately inspected.

This paper focuses on the last explanation by identifying the layers most strongly associated with electrical failures, thereby enhancing the predictability of failure rates. By doing so, process engineers can concentrate their controls specifically on this reduced set of layers, avoiding unnecessary resource allocation in analyzing non-predictive layers. Additionally, we will evaluate the impact of different types of defects on the final electrical failure of dice, aiming to identify which types are most predictive of electrical failures. While, from a predictive standpoint, this analysis may be less intriguing than the one on layers, as all possible defects are examined in the SEM images of wafers, it remains valuable in providing insights to engineers regarding the reasons for failures during the process (e.g., scratches).

The analysis has been conducted independently for the datasets ARES and TETIS, considering two distinct configurations: the entire dataset that was inspected for a generic defect (type =0 ) and dice inspected for a classified defect through SEM (type >0 ). However, for the sake of brevity, the detailed results will be presented only for the latter case and for the dataset ARES, with differences in the results highlighted when pertinent and relevant. Analysis of the dataset TETIS and further analysis are provided in the Supplementary Materials.

Codes for running the methodologies have been developed in the R environment and executed on an AMD EPYC 7301@2.20 GHz processor (16 core), with 256 GB RAM.

### 4.1. Data Collection

Data are provided by STMicrolectronics for each dataset in three different databases in csv format:

- Defect files (5589 for the dataset ARES), one for each layer and wafer of a lot, including information on defects for each die of the wafer, coordinates of dice on the wafer, and some metadata (lot, wafer, product, etc.).
- Test files (2724 for the dataset ARES), including the outcome of the final electrical tests, coordinates of dice on the wafer, and some metadata, e.g., product, layer, wafer.
- Region files (386 for the dataset ARES), including coordinates of dice on the wafer and a few metadata (e.g., layer).

All files are matched through a key provided in the three types of files (Reference).

Not all files of defects have a counterpart in the test files. In particular, there are 197 defect files without a counterpart in region files, and vice-versa, 482 testing files without a counterpart in defect files.

### 4.2. Data Preprocessing

Only a minor cleaning has been necessary in removing duplicated layers for the same lot/wafer, removing files of lot/wafers not having a counterpart in corresponding outcome ones, and vice-versa, outcome files of lot/wafers without a counterpart in the corresponding defect ones (see Section 4.1). They amount to about 1%, 3.5%, and 17%, respectively, of the entire dataset.

Coordinates of the dice on a wafer, necessary for matching defect and outcome of the electrical test data, have to be aligned because they are provided with different reference systems in the two databases. At the end, it has been possible to compute the distance of each die from the center of the wafer.

Moreover, the outcome of the final electrical test has been converted to a binary variable, since the original values were a code representing the type of electrical failures on the dice.

No outlier data were detected, no scaling of variables was necessary, and no imputation was made of missing data according to the procedure described in Section 6.2, assigning the code NA for their representation. At the end of the preprocessing step, all data included in the different defects, the outcome of the electrical tests, and region files have been merged in a unique database for each dataset.

The database has been randomly split for 10-fold cross validation according to the methodology described in detail in Section 6.2.

### 4.3. Data: Layer

**Figure 2:**
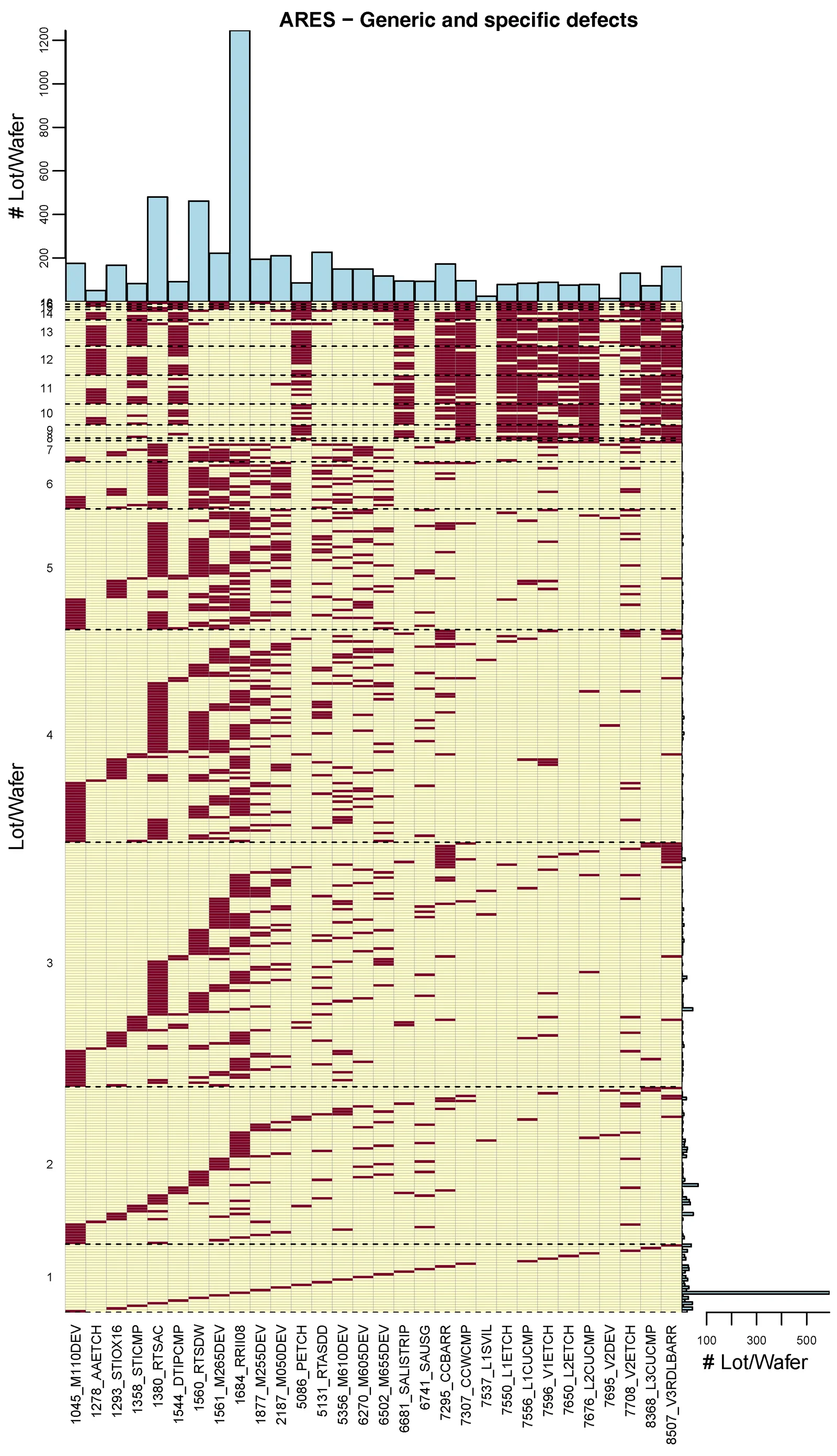
Structure of inspected layers for the dataset ARES, considering dice for which at least one defect of any type (including generic defects of type =0 ) is detected.

Since layer is the key variable of this study, we examine the structure of inspected layers (Figure 2) depicted as a matrix.

The figure is composed of three parts.

The main central part is a visual representation of a matrix: columns represent the layers of the process; rows highlight specific combinations of layers subject to inspection for a same wafer (brown color for inspected layers, ivory for uninspected ones). For instance, the bottom row refers to wafers for which a single layer had been inspected, namely 1045_M110DE, depicted in brown color in the first column of the row. As a further example, the top row shows, in brown color, the 18 layers that have been inspected for the same wafer. Rows of the matrix are sorted by blocks separated by horizontal dashed lines, each block representing the number of layers inspected for each wafer (reported on the left of the rows for each block). Each row of the block refers to different combinations of layers inspected. For example, the lowest block refers to one single layer inspected for the same wafer, and each row of the block indicates the corresponding layer in brown color; as a further example, the top box of the matrix refers to the highest number of layers inspected for the same wafer (18), which occurred in only one combination of layers, represented in brown color in the row of the block.

The right part of the figure, strictly associated with the rows of the central matrix, shows, as a histogram, the number of wafers inspected at the same combination of layers. For example, the highest number of wafers (about 600) corresponds to the unique layer 1684_RRII08 of inspection.

Finally, the third part of the figure (the top *x*-axis histogram) displays the total number of wafers inspected at the layers indicated on the *x*-axis, independently of the number of layers actually inspected. For example, the layer most frequently inspected is again 1684_RII08 (more than 1200 wafers).

The figure refers to the entire dataset, or, equivalently, to dice inspected for defects of any type, including generic defects of type =0 . We observe that the distribution of inspected layers (top histogram of the diagram) is far from uniform. Similarly, the distribution of combinations of inspected layers for a wafer (right histogram) is not uniform and not random, with most cases largely limited to three layers at most. The maximum number of inspected layers is 18, and the number of different combinations of layers inspected is 285. In particular, the modal value of the combinations of inspected layers is reached for the single inspected layer 1684_RRII08. Even though such results are similar to dataset TETIS (compare with Figure S5 of the Supplementary Materials), the criteria for choosing layers subject to inspection by process engineers are different.

The analogous figure corresponding to only specific defects (type >0 ) inspected is deferred to the Supplementary Materials (Figures S6 and S4 for the datasets ARES and TETIS, respectively) because of its main interest for STMicroelectronics. Besides, indications are quite different from the case of generic defects (type =0 ). In particular, the number of inspected layers per wafer is very low, just one in most cases. This means that the second level of inspection (through the SEM) has been very selectively chosen by STMicroelectronics process engineers. Moreover, we observe that the lack of information of the uninspected layers amounts to a fraction of missing data as high as 95.3% for the dataset ARES and 96.6% for TETIS.

Summarizing, the above analysis supports the claim of Section 2 on very high rates of missing data and non-random selection of layers to inspect.

## 5. Odds Ratio Analysis

We first introduce the contingency table in Table 2 for a specific factor (layer, type of defect, or their combination).

Let $N_{df}$ be the number of dice with a status of defect *d* ( d=0 : no detected defect, d=1 : at least one defect detected) and outcome of the electrical test *f* ( f=0 : functional die, f=1 : failed die). Then, the Odds Ratio (Equation (1)) is defined as [47]

(1) $$OR=\frac{\frac{N_{11}}{N_{10}}}{\frac{N_{01}}{N_{00}}}=\frac{N_{11}}{N_{10}}\frac{N_{00}}{N_{01}}.$$

If OR=1 , then the exposure does not influence the odds of the outcome (the factor is neutral or indifferent to electrical failures). When OR>1 , the exposure is associated with higher odds of the outcome (the factor is more closely related to electrical failures than to functional dice; in medical terminology, we refer to this case as a “risk factor”). We do not expect to have variables with Odds Ratio <1 , as this would imply that the presence of defects increases the probability of a successful electrical test.

The analysis of Odds Ratios is endowed with a statistical test for the null hypothesis OR=1 . In this study, Odds Ratio is computed using the median-unbiased estimator (mid-p) and confidence intervals are computed using exact methods (mid-p) [48] using the R oddsratio function. Additionally, given the multiple tests conducted on various subsets, a multiplicity correction is applied using the False Discovery Rate [49].

Odds Ratio analysis will first be conducted separately on each layer and type of defect, and then on each combination layer/type of defect.

The dataset ARES for the Odds Ratio analysis includes 629,195 dice, among which 33,041 exhibited a failed final electrical test (5.3%), which is a failure rate similar to the entire dataset. This implies that the selection of wafers for the investigation of specific defects is not made with a targeted approach but is compatible with a random selection, or that the selection criterion is not effective. We can consider the following subsets:

(i) The dice with a successful final electrical test that do not exhibit any specific defect (type >0 ) amount to 574,231 dice (91.3% of all inspected ones). Although these dice are not directly informative for identifying factors most predictive of an electrical failure, they serve as a control subset for the analysis. Furthermore, their large number indirectly confirms that defects are a reliable indicator of potential electrical failure; their absence serves as a proxy for functional dice.
(ii) The dice with a failed electrical test and at least one detected specific defect of type >0 amount to 4269, which represents 16.3% of the dice inspected having at least a specific defect of type >0 . In practice, this is the rate of failure of the subset of dice having at least one specific defect of type >0 , which is much higher than the overall rate for the inspected dice (4.2%).
(iii) The dice with a final electrical test that was successful but with at least one specific detected defect (type >0 ) amount to 21,923 (3.5% of the dice inspected). This subset provides information on factors that are not associated with a final electrical failure of dice.
(iv) The electrically failed dice without any detected defect of any type constitute a group of 28,772 dice (4.6% of inspected dice). These are particularly challenging because there is no direct information on factors related to a final electrical failure. The failure rate within this subset is 4.8%, which is lower than the rate in the entire inspected set. The possible causes for this behavior have already been discussed in Section 4.2.

Subsets (i) and (ii) establish a direct relationship between the presence/lack of electrical failures and the presence/lack of defects, respectively, covering a combined 87.6% of the entire inspected set.

Table 3 presents the Odds Ratio analysis layer by layer. Contingent upon the layer, it provides the following: the number of inspected dice; the contingency table forming the basis of the Odds Ratio (Table 2); the failure rate for all dice inspected at a specific layer; the value restricted to the subset showing at least one defect of any type; the values of the Odds Ratio; the corresponding *p*-value resulting from the test of the null hypothesis OR=1 , corrected for multiplicity. Entries in the table are sorted by *p*-value in decreasing order; layers without dice showing electrical failure and any type of detected defect are excluded.

If a layer is not predictive of an electrical failure, the values of the failure rate for all dice and for those inspected are similar: the higher the failure rate, the stronger the association between defects and a condition of faulty dice, and the better the predictability of a failure.

The same analysis for the subset of dice inspected for generic defects (type =0 ), which coincides with the entire dataset, is deferred to Table S5 for the sake of brevity.

Table 4 presents the same analysis for the type of defect. Since all defects are inspected by SEM at each layer, the number of involved dice is the same for each type of defect (equal to the size of the subset), and the corresponding failure rate is also the same. The last row of Table 4 reports the results for the case of type of defect =0 , for which the entire dataset has been inspected.

The analyses presented in Table 3 and Table 4 consider layers and types of defects separately, neglecting their interaction. This could obscure crucial predictability of specific defects at specific layers. Therefore, the Odds Ratio analysis was extended to all pairs of layers and types of defects. The complete table of results for the datasets ARES and TETIS are deferred to the Supplementary Materials as Tables S6 and S4, respectively. They can be valuable for process engineers.

**Figure 3:**
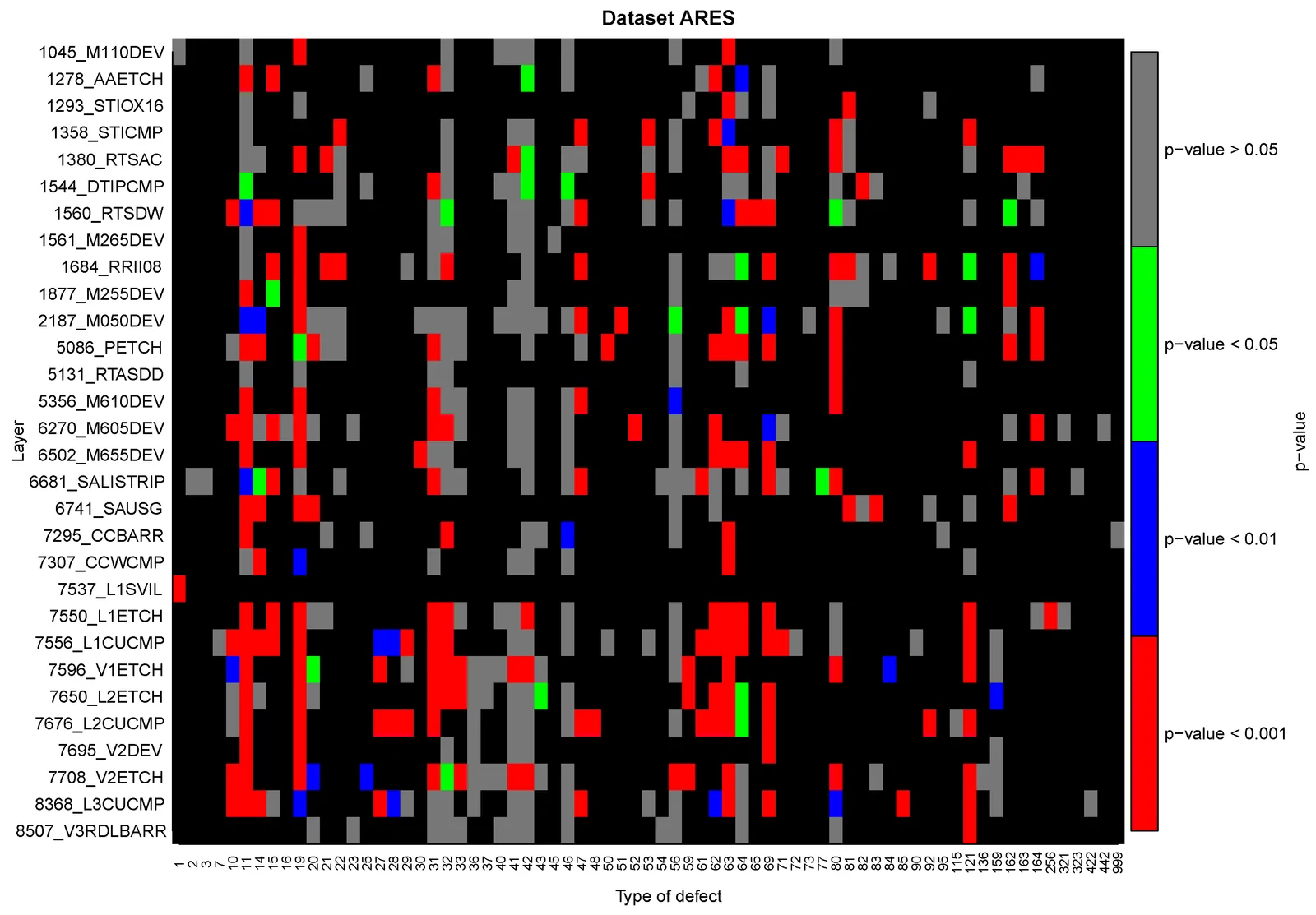
Matrix plot depicting the significance of the test OR=1 based on layers (rows) and types of defects (columns) for the dataset ARES. Color representation is assigned according to the *p*-value of the test: red for p-value≤0.001 , blue for 0.001<p-value≤0.01 , green for 0.01<p-value≤0.05 , and gray for p-value>0.05 (not significant). The background in black indicates an undefined test.

Figure 3 illustrates the resulting Odds Ratio analysis through a matrix in graphical form. Rows correspond to layers, and columns represent the types of defect. Each pair is colored based on the *p*-value of the corresponding test for OR=1 , as indicated in the legend. Black cells indicate undefined values of Odds Ratio, primarily due to a lack of useful data, such as the absence of defects.

**Figure 4:**
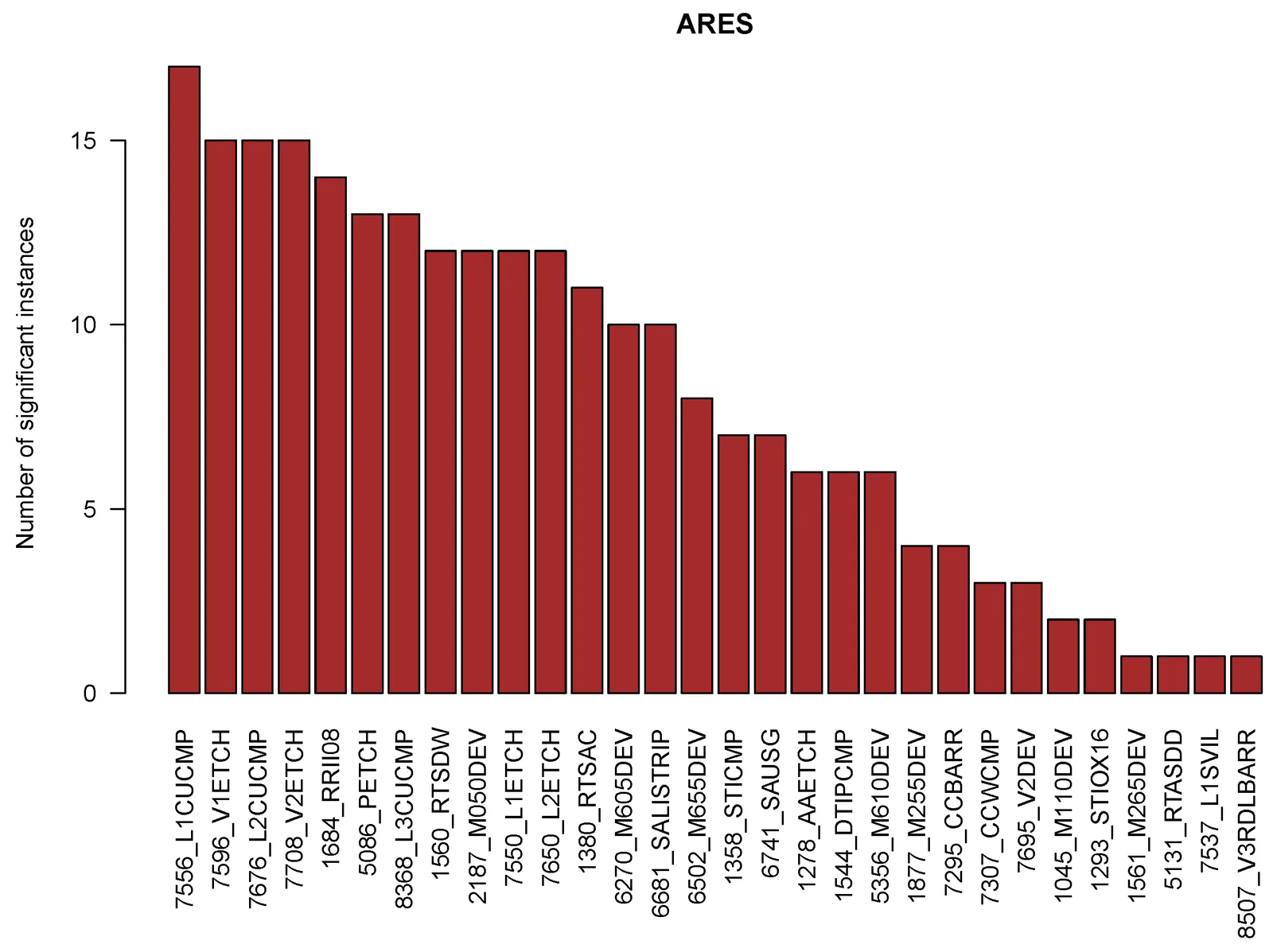
Bar chart illustrating the number of significances by layer, derived from the Odds Ratio analysis on pairs of layers and types of defects for the dataset ARES (significance level 0.001).

**Figure 5:**
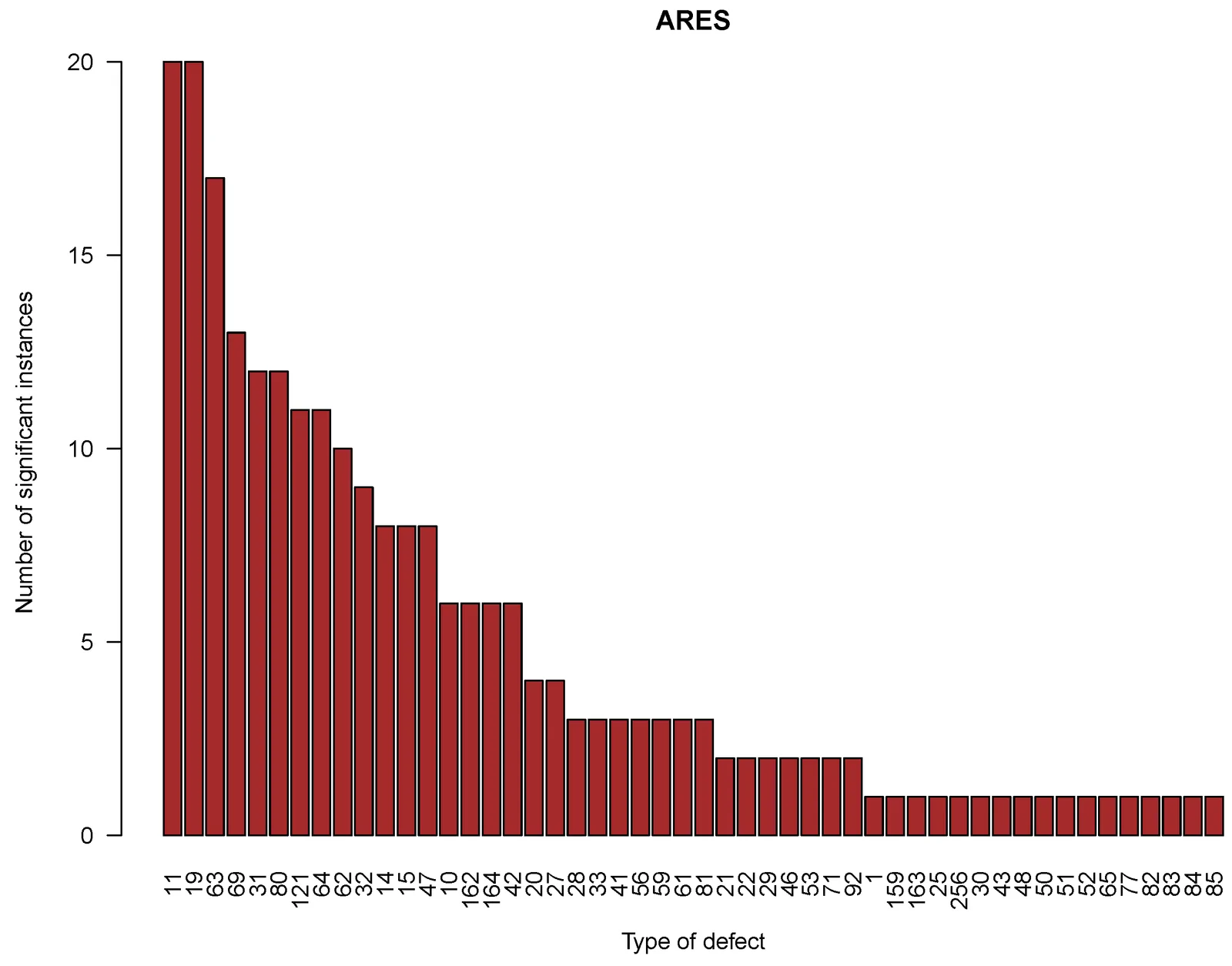
Bar chart illustrating the number of significances by type of defect, derived from the Odds Ratio analysis on pairs of layers and types of defects for the dataset ARES (significance level 0.001).

For a clearer understanding of the most significant layers and types of defects, Figure 4 and Figure 5 present the distribution of the number of significances detected for layers and types of defects, respectively, through the Odds Ratio analysis on the pairs of layer and type of defect (significance level 0.001).

## 6. Prediction of Electrical Failures

This section introduces the model for predicting the outcome of the electrical test for each die from defects detected during the process. Once we obtain a die-by-die estimate of the electrical test results, it becomes possible to straightforwardly estimate the production yield, for example at the wafer level, by aggregating predictions for the corresponding dice.

### 6.1. Predictor Variables

We begin listing the predictors of the model other than layer and type of defect, already proved by Odds Ratio analysis in Section 5 to be associated with the outcome of the electrical test.

#### 6.1.1. Wafer Slot

**Figure 6:**
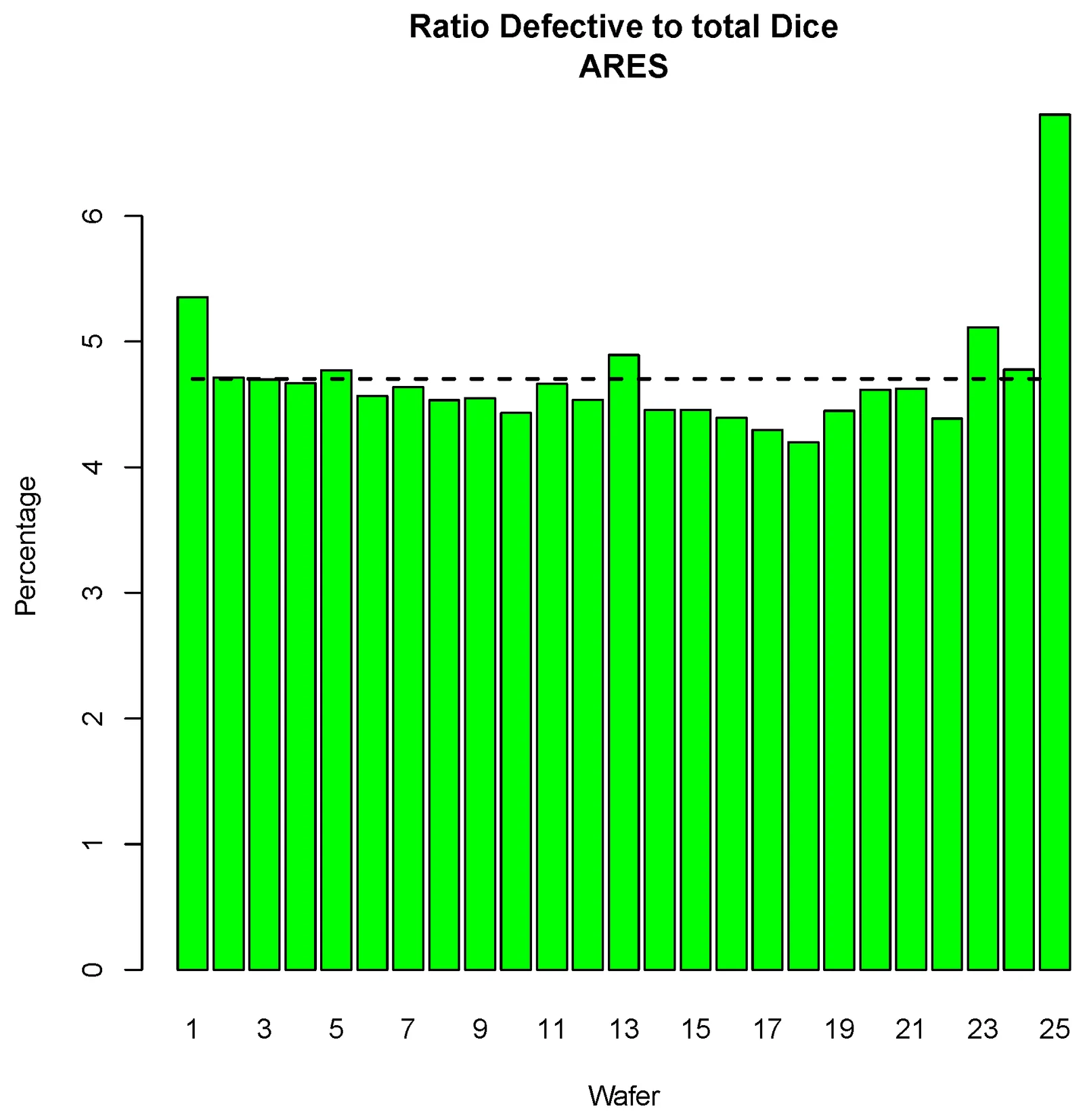
Bar chart illustrating the average rate of electrically failed dice by wafer slot for the dataset ARES. The dashed horizontal line indicates the global average rate of electrical failures for the dice.

Figure 6 depicts the average failure rate of dice per wafer slot. The failure rate is not uniform across wafer slots, with slot 25 and, to a lesser extent, slot 1 showing a higher rate of failure. This observation is supported quantitatively by an ANOVA analysis on the failure rate for each wafer slot, yielding a *p*-value numerically close to 0. Subsequent post hoc analysis using Tukey Honest Significant Differences (HSD) with multiplicity correction reveals that the average failure rate for wafer slot 25 is significantly different from all other slots, and wafer slot 1 is significantly different from the other slots. No other significant differences are observed among the remaining wafers slots. In practice, we can cluster the wafer slots into three different behaviors: wafer slot 1, 25, and all the other ones. This observation has been confirmed through interaction with STMicroelectronics engineers, particularly regarding wafer slot 25.

#### 6.1.2. Location of Dice on the Wafer

**Figure 7:**
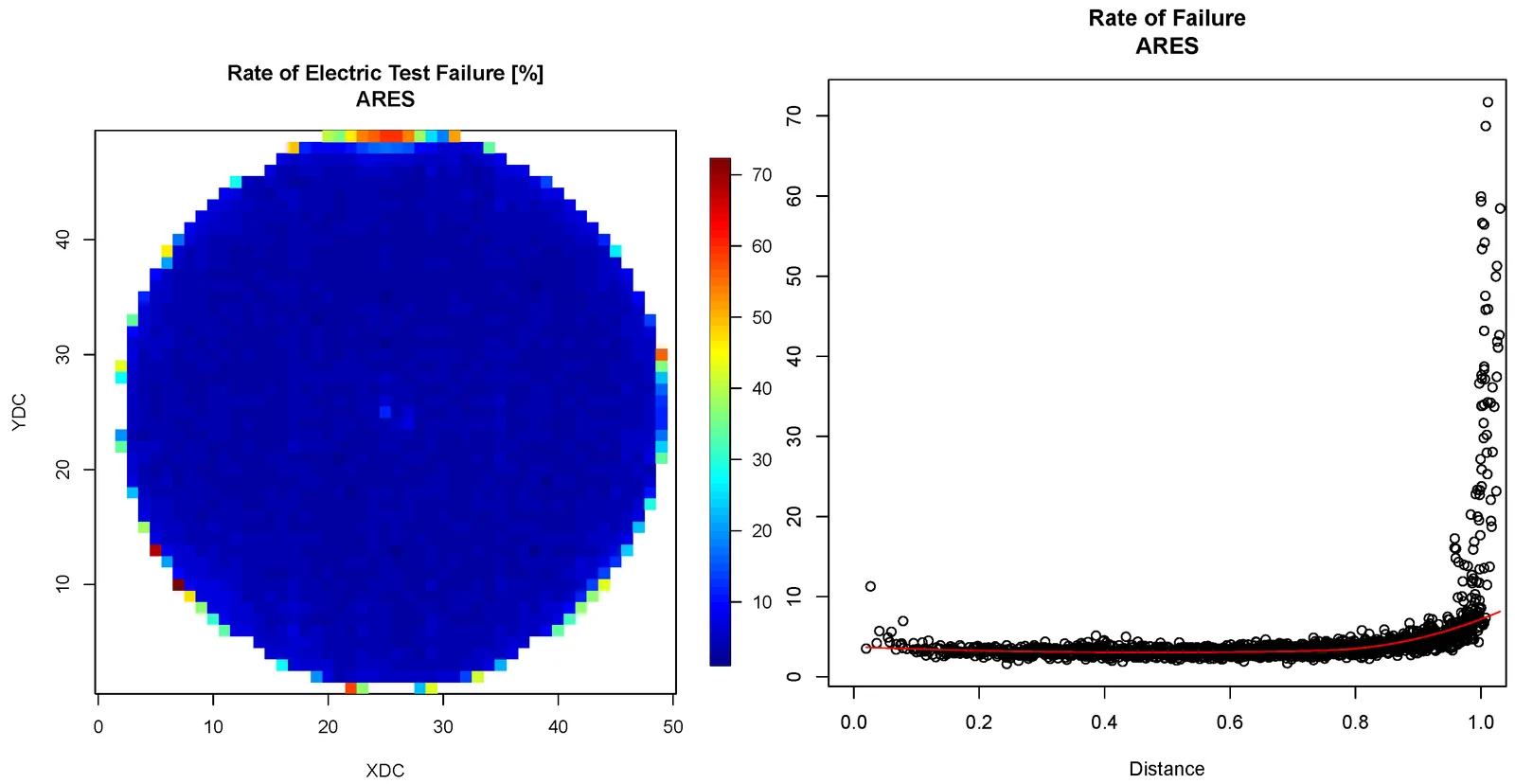
Spatial distribution of electrical failures on the wafer (**left**) and plot of the rate of electrical failures of dice vs. the distance from the center of the wafer, normalized to the range [0, 1] (**right**) (the red line represents LOWESS nonparametric smoothing). Plots refer to the dataset ARES.

Figure 7 (left) illustrates the spatial distribution of the average rate of electrical failures.

We observe that the majority of failures are concentrated at the boundary of the wafer, reaching high values that exceed 70%. To better visualize the global effect, Figure 7 (right) presents a scatter plot of the rate of electrical failures against the distance of the die from the center of the wafer (normalized to 1).

The impact of failures on the boundary is highly evident. In other regions of the wafer, the average rate of failure remains relatively constant up to a (normalized) distance of 0.75–0.80 (depending on the dataset). Beyond this point, it gradually increases before steeply rising at the boundary.

Summarizing, Table 5 presents the list of predictors considered in the models.

### 6.2. The Prediction Model—No Interaction Layer-Type of Defect

The outcome of the model is the result of the electrical tests on each die (binary variable *Y*: faulty (or 1) if at least one test fails, functional (or 0) if all tests are successful).

As is well-known, many binary classification methods are actually regression models that basically give as output a continuous variable representing the probability ( $y_{f}$ ) of one of the two classes (in our framework failure, coded as 1). Afterward, the final binary prediction, *Y*, can be obtained through a cutoff *c* as (see, e.g., Fawcett [50] also for a discussion on its choice)

(2) $$Y=\left\{\begin{array}{cc} \mathrm{faulty} & \mathrm{if}\;y_{f}\geq c,\\ \mathrm{functional} & \mathrm{if}\;y_{f}<c. \end{array}\right.$$

To avoid overfitting, the following cross-validation scheme will be adopted: the dataset is first purely randomly split according to a Hold Out configuration as 70% (training+validation) and 30% (test). The former 70% part is purely randomly split by 10-fold cross-validation as training and validation. The 10-fold cross-validation is used by the classification method to train the model on the training folds and then to evaluate it on the validation ones for choosing hyperparameters. At the end, once the hyperparameters have been tuned, the classification model is again estimated once on the full 70% training+validation subset using these hyperparameters. Finally, this last model predicts the results of the electrical test on each die in the 30% test subset, which is independent of observations used to train the model and tune its hyperparameters.

Conventional classification methodologies rely on classification accuracy to evaluate the classifier’s performance and tune its hyperparameters. While global accuracy is a natural metric, it can be criticized, especially when the sample is imbalanced between the two classes [51]. Consequently, various alternative indicators have been developed in the literature (e.g., [52,53]), each with its own advantages and drawbacks and with its own optimal cutoff, generally different.

The key point is that Type-I and Type-II errors cannot be treated in the same way due to their different consequences. For example, in the current project, predicting a faulty die as functional incurs a different economic cost than predicting a functional die as faulty. Similarly, in medical practice, the consequences of an undiagnosed disease are often more severe than those of an erroneously diagnosed disease, which can be further examined through subsequent medical tests.

In other words, the choice of the cutoff is not determined solely by minimizing a statistical indicator but is based on how much a user is willing to tolerate Type-I and Type-II errors. Consequently, a user can select the indicator that best fits their balance of Type-I and Type-II errors. In practice, users choose the optimal cutoff (or indicator) based on the associated costs (economic, human lives, etc.) related to the errors committed with this choice. In this respect, see also [42], who propose in a more general way to optimize the inspection of layers based on the cost of inspection and of failed dice/wafers.

The following indicators have been considered, expressed in terms of True Positive ( TP ), True Negative ( TN ), False Positive ( FP ), and False Negative ( FN ).

- Total accuracy [54]:
(3) $$\mathrm{Accuracy}=\frac{TP+TN}{TP+TN+FP+FN}.$$
- Sensitivity and Specificity [54]:
$$\mathrm{Sensitivity}=\frac{TP}{P},\;\mathrm{Specificity}=\frac{TN}{N}.$$
- The Cohen kappa statistic ( κ ), a chance-corrected accuracy measure [55]:
$$\kappa =\frac{2TP\times TN-FN\times FP}{\left(TP+FP)(FP+TN)+(TP+FN)(FN+TN\right)}.$$
  Criticism has been expressed in the literature regarding its use [56]. Also note that κ=0 for a random model based only on the sample size of the classes. Therefore, negative values are possible, indicating a model that is worse than random.
- *G*-mean, the geometric mean of Specificity and Sensitivity [57], suitable for unbalanced datasets:
$$G\text{-}\mathrm{mean}=\sqrt{\mathrm{Sensitivity}\times \mathrm{Specificity}}=\sqrt{\frac{TP\times TN}{P\times N}}.$$
- *F*-1 score, the harmonic mean between Precision and Recall [54], also suitable for unbalanced datasets:
$$F\text{-}1=\frac{2TP}{2TP+FP+FN}.$$
- Matthews Correlation Coefficient (MCC) [58].
$$\mathrm{MCC}=\frac{TP\times TN-FP\times FN}{\sqrt{\left(TP+FP)(TP+FN)(TN+FP)(TN+FN\right)}}.$$
  In contrast to *F*-1, MCC and *G*-mean are symmetric indicators—their value does not change if one swaps Positives and Negatives. Recent literature [59] asserts that MCC is superior to several alternatives.

Optimization of each indicator on the validation subset yields its own optimal cutoff. Finally, we mention the Area Under the Curve (AUC), based on the area under the Receiving Operating Characteristic (ROC) curve Sensitivity vs. (1−Specificity) , related to the prediction method itself and not to the cutoff.

Analysis of this section is based on the datasets restricted to dice inspected for specific defects through SEM (type >0 ), specific defects being much more predictive of a final electrical failure than generic defects.

As mentioned in Section 2, we used XGB as a classification/regression engine. XGB requires a loss and an evaluation function. Different choices were explored in this study (e.g., mean square error, logistic regression, accuracy with a fixed cutoff) and compared based on AUC, but they yielded very similar values in terms of optimal indicators, even though the corresponding optimal cutoffs varied significantly.

Additionally, a comparison was made with Random Forest-based methods again endowed with different losses and evaluation metrics. Random Forest, also based on Decision Trees, shares the nice property of handling missing data as information. Results of the comparison show that XGB is more accurate than Random Forest according to the AUC indicator. They are are not shown for the sake of brevity.

**Figure 8:**
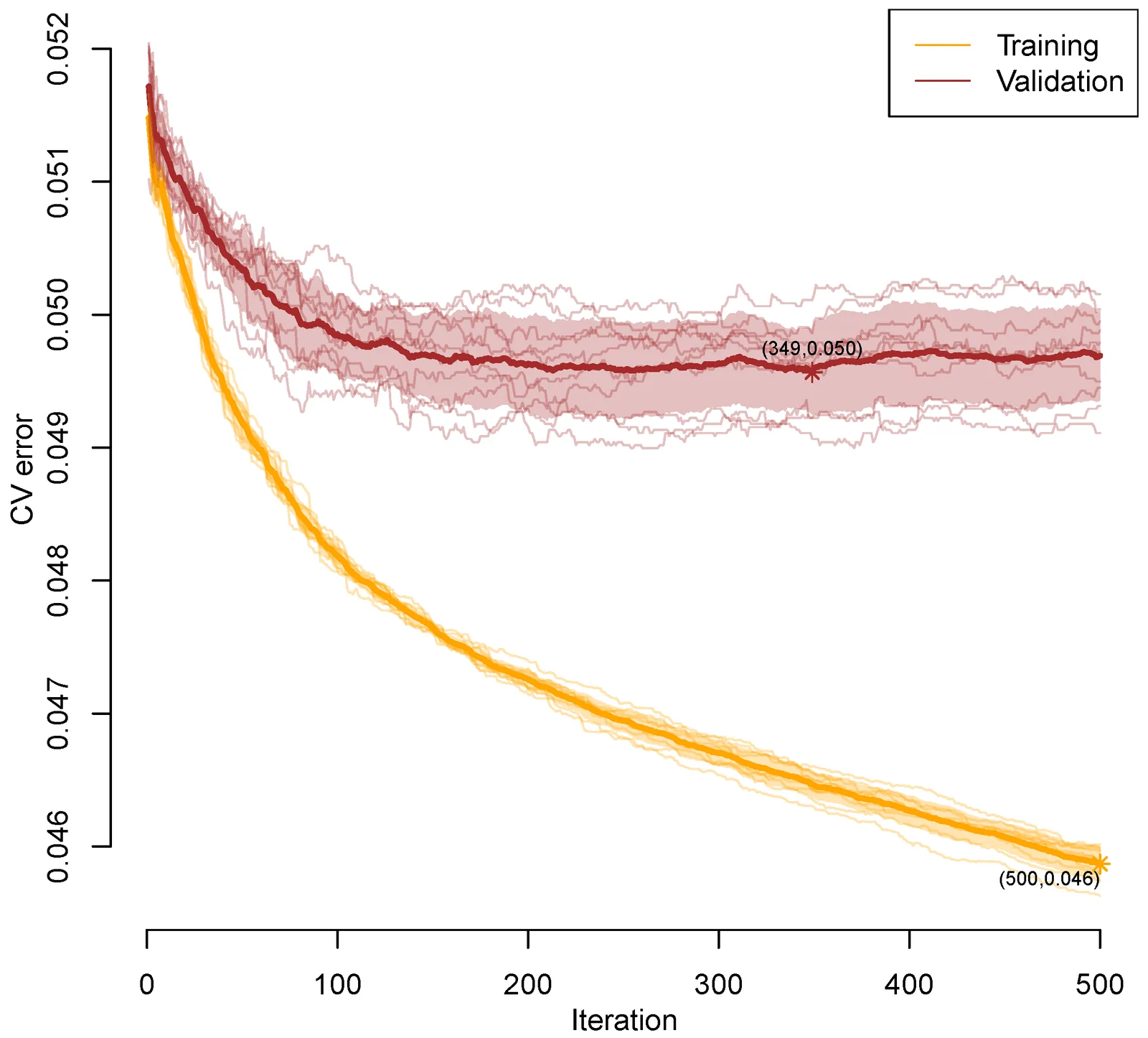
Error curve of the training and validation subsets in 10-fold cross-validation for the XGB model with logistic loss and Accuracy as cost and evaluation function, respectively, on the ARES subset containing only dice inspected for defects of type >0 . Solid thick lines represent the average value over the 10 folds, shaded areas correspond to one standard deviation, and thin lines depict the 10-fold cross-validation curves.

Figure 8 displays the training and 10-fold indicator Accuracy for XGB endowed with objective and evaluation functions given by logistic loss and Accuracy, respectively.

**Figure 9:**
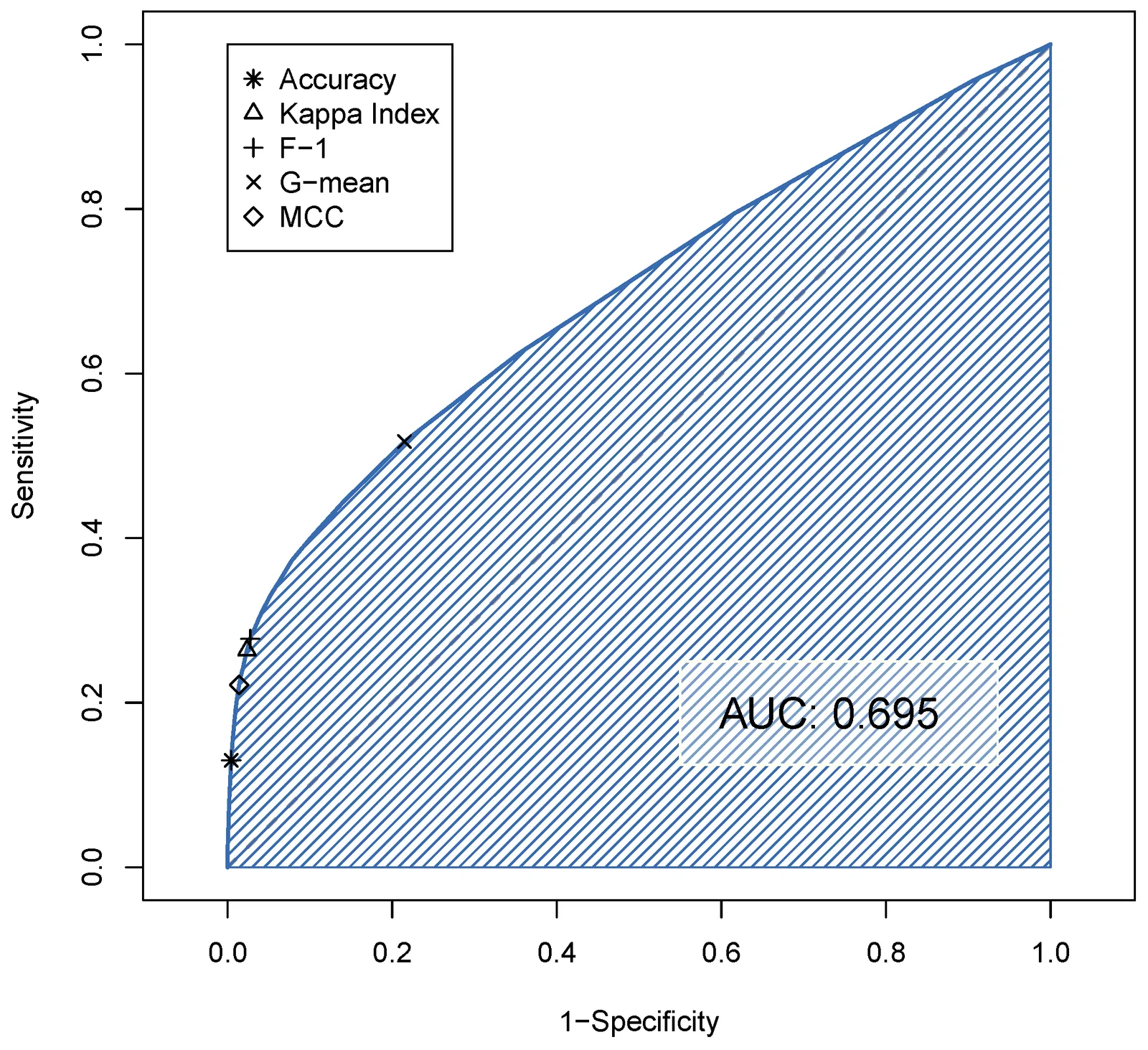
ROC curve for the XGB model with logistic loss and Accuracy as cost and evaluation function, respectively, on the ARES subset containing only dice inspected for defects of type >0 . Optimal values of the indicators are reported on the curve according to the legend.

The ROC curve is shown in Figure 9 with AUC=0.7 .

**Figure 10:**
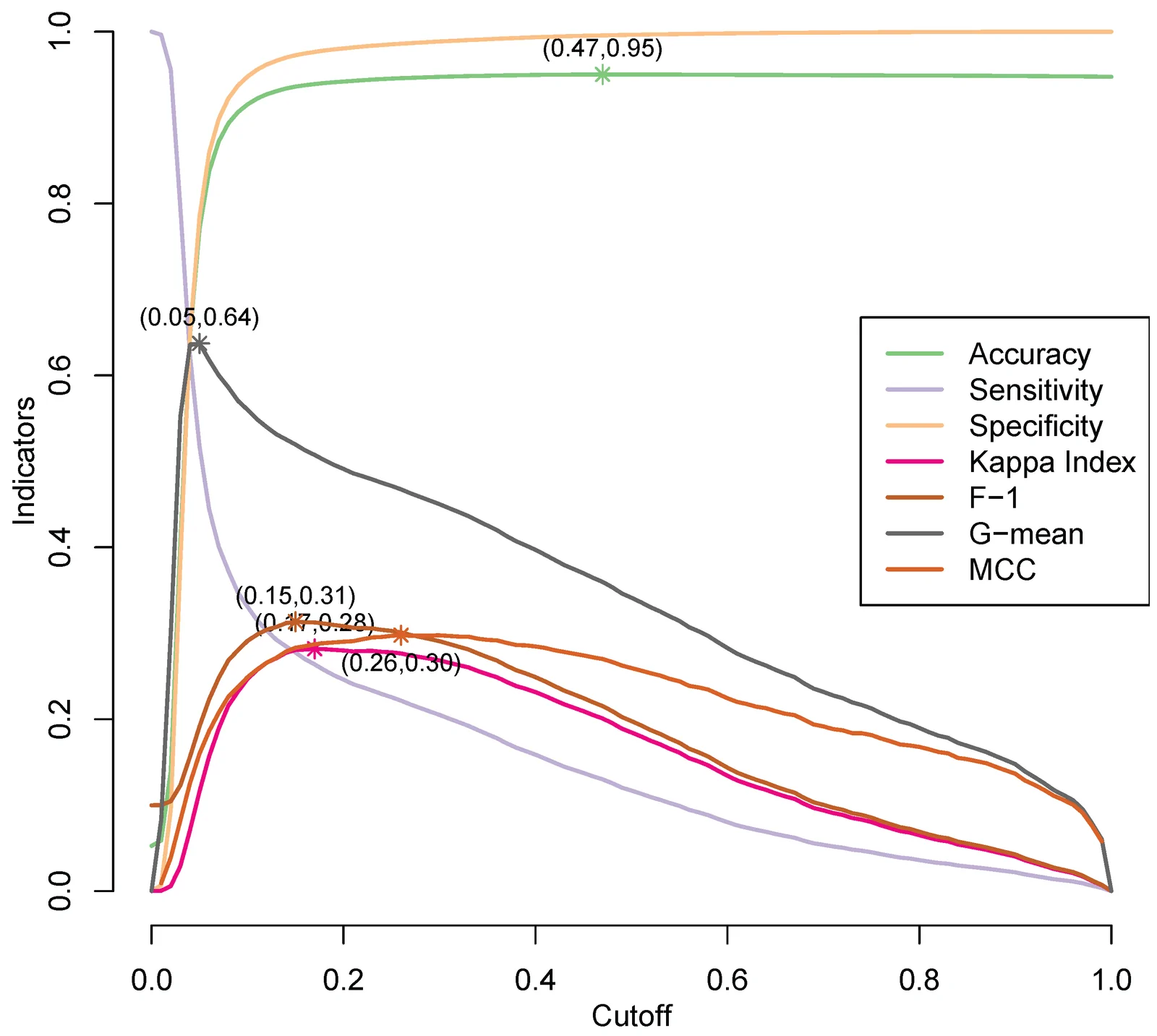
Indicators of accuracy vs. cutoff for the XGB model with logistic loss and Accuracy as cost and evaluation function, respectively, on the ARES subset including only dice inspected for defect of type >0 . Optimal values are marked with an asterisk along with the corresponding values of the cutoff and the indicator.

Figure 10 displays the accuracy indicators considered in this study plotted against the cutoff.

Table 6 summarizes the optimal values of the indicators and their corresponding cutoffs. For each considered indicator (row, presented in the ‘Indicator’ column), the table includes the optimal cutoff (‘Cutoff’ column), the corresponding values of Sensitivity and Specificity, and the corresponding value of all indicators.

**Figure 11:**
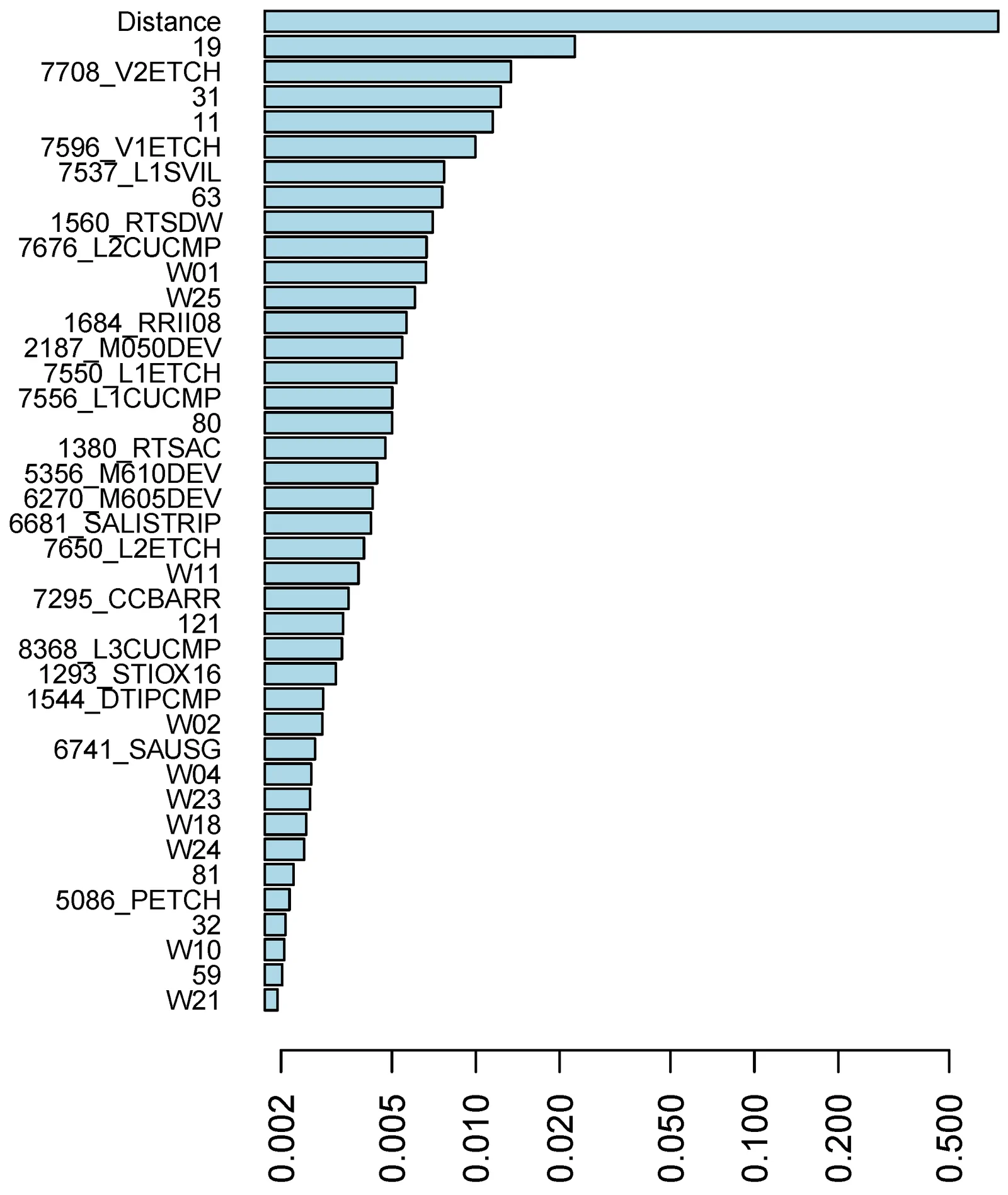
Importance of predictors in the XGB model with logistic loss and Accuracy as cost and evaluation functions, respectively, applied to the ARES subset that includes only dice inspected for defects of type >0 .

Finally, to evaluate and compare the effect of each predictor in the model, we considered the “importance” of variables in a tree-based model. It is based on how much the performance measure of the model improves at a split point of a Decision Tree due to the variable, weighted by the number of observations the node is responsible for. Average values over all trees involved in Gradient Boosting are considered. Figure 11 presents the most important predictors.

### 6.3. Prediction Model—With Interaction Layer-Type of Defect

**Figure 12:**
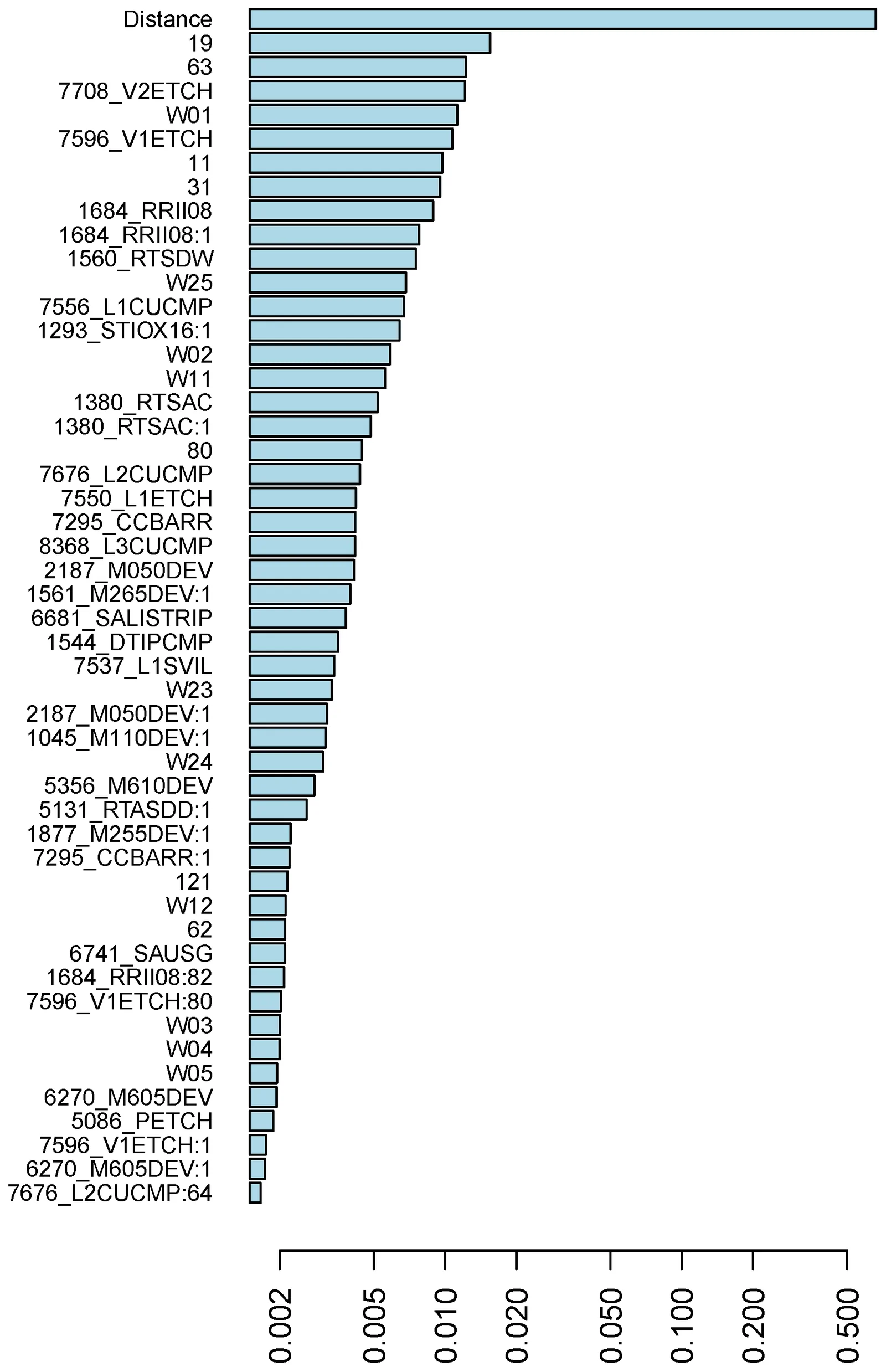
Importance of predictors in the XGB model with logistic loss and Accuracy as cost and evaluation functions, respectively, applied to the ARES subset that includes only dice inspected for defects of type >0 . The prediction model includes interaction between layers and types of defects. Layers are indicated by their name and types of defects by their code. Names of couple layer-types of defects are separated by a column (:).

We ran the same model of Section 6.2 with the inclusion of interaction layer-type of defect. The total number of variables of the model is 2257 with 629,195 dice. We recall that only 26,192 dice show at least one specific defect (4.2%) and only 33,041 dice underwent an electrical failure (5.3%). The model is not able to predict failures not related to defects, or related to defects not detected by SEM. Results are shown in Figure 12, where the most important 50 variables are graphically shown, sorted according to the indicator discussed in Section 6.2.

### 6.4. Prediction Model—Yield of a Wafer

In this section, we estimate the yield for each wafer of a lot by aggregating predictions of the corresponding dice using the classification/regression method introduced earlier in Section 6.2 and Section 6.3.

In this study, we define the rate of failure of a wafer, $RF_{w}$ , as

$$RF_{w}=1-Y_{w}=\frac{F_{w}}{N_{w}},\;{\tilde{RF}}_{w}=1-{\tilde{Y}}_{w}=\frac{{\tilde{F}}_{w}}{N_{w}}.$$

with $Y_{w}$ being the Yield of the wafer, $F_{w}$ and $N_{w}$ the number of electrically failed dice and of dice on the wafer, respectively; a tilde indicates values predicted by the models of Section 6.2 and Section 6.3.

We introduce the classical error indicators RMSE (Root Mean Square Error) and MAE (Mean Absolute Error) as:

(4) $${\mathrm{RMSE}}_{w}=\sqrt{\frac{1}{N_{w}}\sum_{i=1}^{N_{w}}{\left({\tilde{RF}}_{w}^{i}-RF_{w}^{i}\right)}^{2}},\;{\mathrm{MAE}}_{w}=\frac{1}{N_{w}}\sum_{i=1}^{N_{w}}\left|{\tilde{RF}}_{w}^{i}-RF_{w}^{i}\right|,$$

with apex *i*, $i=1,\ldots ,N_{w}$ , referring to the dice belonging to the wafer *w*.

To avoid hyperfitting, we chose the cutoff that minimizes the RMSE during the training/validation step of the prediction models by 10-fold cross validation, and error indicators (Equation (4)) are estimated on the final test subset that is separate from the training/validation one. Results are summarized in Table 7 for both the datasets ARES and TETIS.

**Figure 13:**
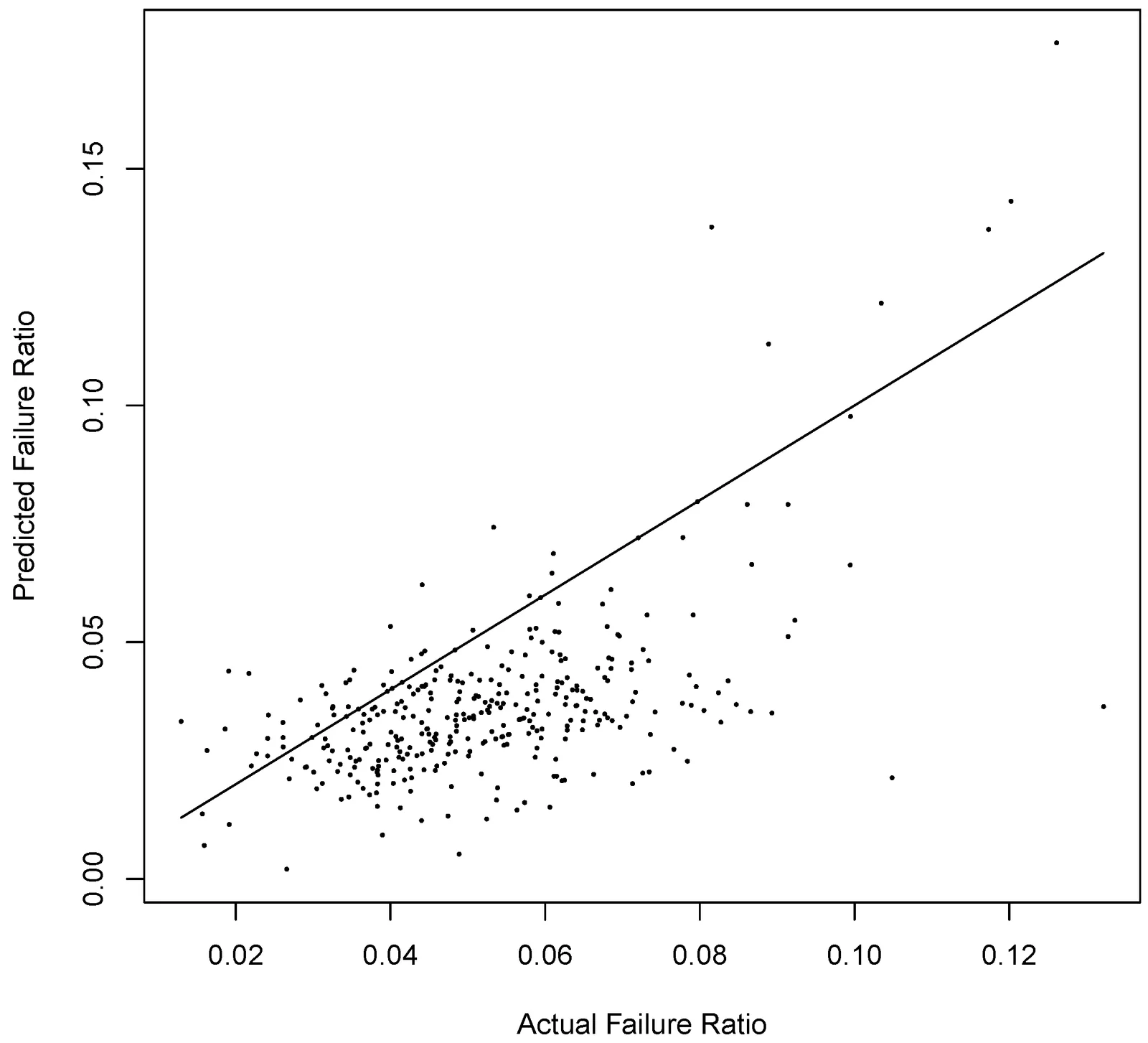
Scatter plot of predicted rate of failure vs. actual one at wafer level. Estimations are obtained aggregating the results of the classification/regression model without the interaction of layers and types of defects. Results refer to the dataset ARES. The diagonal line is the ideal prediction equal to the actual values.

To analyze the results more deeply, Figure 13 reports the scatter plot of the wafer rate of failure without interaction between layers and types of defects.

Similar results are obtained when aggregation is made on the results of the model with interaction between layers and types of defects, deferred to the Supplementary Materials.

## 7. Discussion

### 7.1. Odds Ratio

Odds Ratio analysis with respect to layers in the case of specific defects (Table 3) reveals that most layers are predictive: at almost all layers, the presence of defects significantly predicts a failure of the final electrical test. However, the degree of prediction strongly depends on the layer. As a guideline, considering that the average rate of failure for the entire dataset ARES inspected for specific defects by SEM, with any specific defect found, is 16% (as per Table 1), we can infer that the most interesting layers are the ones exhibiting a rate of failure in the presence of defects higher than 16% and a very low *p*-value. Specifically, these are the entries in Table 3 up to 7695_V2DEV and from 1278_AAETCH to 1045_M110DEV. Nevertheless, there is a high number of dice without defects but with an electrical failure (their number is ranked second in the contingency tables).

If we extend the dataset to the dice inspected for generic defects (type =0 ), which coincide with the entire dataset, the results shown in Table S5 overlap very well in terms of significant layers. We also notice that the failure rate for dice showing at least one generic defect (type =0 ) is lower than the subset of dice with at least one specific defect (type >0 ). In other words, also generic defects (type =0 ) detected on layers result in being associated with an electrical failure of the dice, to, however, a lesser extent.

The analysis of Odds Ratio with respect to types of defect reported in Table 4 shows that the number of predictive types of defect is about one-half of all possible types (41 out of 75 at the 95% confidence level). It also includes types for which only a few dice were found with defects. They were retained despite their ambiguity because, in many cases, they show a high failure rate (in some cases equal to 1, meaning that all dice with at least one defect are faulty), which could be an indicator of very specific and selective predictors of a failure, to be investigated by process engineers. In the case when no dice are found with a successful electrical test and no detected defects, the Odds Ratio and *p*-value of the corresponding test OR=1 are not defined. From the last row of Table 4, we notice that even defects of type =0 are significant, although with a moderate value of the Odds Ratio and of the failure rate in the presence of defects.

The analysis of Odds Ratio for the interaction of layers and types of defect, reported in Table S6 and visually in Figure 3, shows several interesting features: the lack of significance for defect types from 256 onwards; a prominent vertical structure of significances, indicating the propagation of defects across consecutive layers; and the identification of the most significant types of defects.

The comparison of Odds Ratio for defects (Table 4) and for layers (Table 3) with their interaction (Table S6) reveals a consistency between the most significant layers and types of defects, in the sense that top-ranked layers and defects (Figure 4 and Figure 5, respectively) have the lowest *p*-values for the statistical test of the null hypothesis OR=1 . It is noteworthy that the information included in the analysis with interaction is richer, and very specific defects detected at particular layers can exhibit extremely high values of Odds Ratio and rates of failure (above 90%).

Comparison of significant layers found when generic or specific defects are included (Table S5 and Table 3, respectively) reveals a very good agreement in both cases. However, the failure rate when defects are detected is lower for generic defects.

The dataset TETIS shows similar patterns. Comparison of the most significant types of defect between datasets ARES and TETIS (bar charts in Figure 5 and Figure S3 of the Supplementary Materials, respectively) gives impressive results: the top two most significant types of defect (11 and 19) are the same for both datasets; in addition, four defects are in common among the six top-ranked types (including also defects of type 31 and 80), and nine types are shared within the first ten top-ranked types (adding types 32, 62, 63, 69, and 121). In practice, there is an excellent match of significant types of defects between different products. This means that predictability of the electrical failure of dice depends on the intrinsic structure of defects and not on the product, enhancing the results of the Odds Ratio analysis performed so far and fostering application of the methodology also to other products.

These results reach the objectives (a) and (c) of the paper to find the layers mostly associated with the outcome of the final electrical tests of the dice making least assumptions on the missing data. In this way, process engineers can limit the inspection of layers to those ones with the two-fold result of decreasing inspection costs and abating the number of missing data.

As far as layers are concerned, interaction with STMicroelectronics confirmed that layers between datasets ARES and TETIS cannot be compared because of the different products.

### 7.2. Prediction

We begin with the subset of dice inspected for specific defects (type >0 ). Figure 8 shows that overfitting, potentially possible with XGB, is controlled by choosing the number of iterations as 349. All results provided in this paper for the classification/regression models refer to one realization of the random generation process involved in several steps of the entire methodology (e.g., definition of the training, validation, and test subsets). However, conclusions are consistent among different random realizations.

From Figure 10, we observe that indicators generally reach their optimal values at quite different cutoffs. The curves of Sensitivity and Specificity provide insights into their values corresponding to the optimal points of the indicators (also depicted on the ROC curve in Figure 9).

Table 6 shows that different indicators can lead to significantly different cutoffs and corresponding values for other indicators. We also notice the low value of Sensitivity and the high value of Specificity. In practice, the methodology underestimates the probability of the electrical failure of a die, with the consequence that faulty dice are poorly predicted. On the contrary, prediction of functional dice is very good. Interestingly, optimal cutoffs are very similar between the datasets ARES and TETIS (compare Table 6 and Table S8).

Analysis of importance of predictors in the XGB model (Figure 11) indicates that the most important one is distance. Additionally, we notice the significance of slots Wafer25 and Wafer01, confirming the results in Section 6.1.1. Moreover, the most important types of defects (19, 31, 11, 63) are ranked first in the Odds Ratio analysis on pairs of layers and types of defect (Figure 5), and three out of the four most important layers (7556_L1CUCMP, 7596_V1ETCH, and 7676_L2CUCMP) are ranked among the four most significant ones in the Odds Ratio analysis (Figure 4). In other words, the regression results are fully consistent with the Odds Ratio analysis. This result strengthens objectives (a) and (c) of this study.

Results for the TETIS dataset, shown in Supplementary Materials, are similar to the ARES dataset.

Comparing the most interesting features between the datasets ARES and TETIS as resulting from the regression analysis (Figure 11 and Figure S12, respectively), the agreement is impressive: distance is ranked first in the two datasets, and wafer slots 1 and 25 are common to both datasets. Moreover, the most important five types of defect (11, 19, 31, 63, 80) are the same in the two datasets.

Agreement is found also comparing noninteractive and interactive regression/classification models (Figure 11 and Figure 12, respectively). We again see that distance is by far the most predictive variable. If we analyze the interaction between layers and types of defects, we see that some of them are included among the most important ones, and most of them involve the type of defect 1.

Finally, we consider the prediction model of yield aggregating results of the die-by-die prediction model of the outcome of the electrical tests. Table 7 shows that the estimated RMSE and MAE are high when compared with a reference scenario where the predicted rate of failure is constant and equal to the average rate of failure of the analyzed dataset (restricted to dice investigated for specific defects, type >0 ), reported in Table 1 and here repeated in the first row of Table 7 for convenience. No improvement of the indicators is evident when the model with interactions between layers and types of defect is considered. Scatter plots of Figure 13 and Figure S15 show a significant bias in that predicted rate of failure is almost systematically lower than the actual one. In other words, the die-by-die model underestimates the number of faulty dice. This is consistent with the results of the die-by-die model. In particular, in correspondence of the optimal cutoff with respect to the aggregate yield estimated (0.17 and 0.20 for the datasets ARES and TETIS, respectively), one has Sensitivity =0.26 and 0.20 , respectively, for both models without and with interaction of layers and types of defects. In other words, only about one quarter and one fifth of faulty dice, respectively, are correctly predicted as faulty. As a consequence, the confusion matrix of the classification/regression method also reports a high number of false negatives. This could be mainly due to the presence of a large fraction of dice without any specific detected defect but with an electrical failure, as reported in Table 1 of Section 4. We mention that they represent the majority of faulty dice subject to inspection (87–90% depending on the dataset). On the contrary, Specificity reaches values as high as 0.97–0.98.

The results of this section only partially reach objective (b) of this paper, in that a prediction model of yield derived from an underlying model that predicts the outcome of the final electrical tests has been developed that is able to indicate the best predictive features; however, accuracy is not at the level of an operational model. We believe that this behaviour can be partially explained by the following motivations:

(i) the low number of inspected layers (1.2 and 1.4 per wafer in the average for the datasets TETIS and ARES, respectively, when specific defects are investigated), equivalently the high fraction of missing layer data (95.3% and 96.6% for the datasets ARES and TETIS, respectively);
(ii) the high fraction of electrically failed dice without any specific detected defect (see Section 4), which the model predicts as functional;
(iii) the limited amount of lots and wafers, which also decreases the power of the statistical tests of this study.

## 8. Conclusions

### 8.1. Summary and Contribution

We conducted a study to find layers mostly predictive of the outcome of the final electrical tests on dice. Additionally, we developed a model to predict the failures of individual devices and ultimately the yield of the production. Two different datasets from STMicroelectronics related to different products were the source of the data.

To achieve these objectives, we employed two primary statistical tools: (1) Odds Ratio analysis, which, when applied to single layers or types of defects or defects detected at a specific layer, allows us to mitigate the challenges posed by the significant sparsity of data; (2) Gradient Boosting for prediction models. These tools provided insights into the most influential types of defects and layers.

The study was particularly challenging due to the selection of inspection layers by process engineers with criteria that were not disclosed. This led to a highly sparse and non-random selection of inspected layers, resulting in a substantial rate of missing information in the model, reaching as high as 95–96% for the layer predictor.

Both analyses agreed in identifying the most efficient predictors (types of defects and layers) for electrical failures of individual devices. Cross-comparison of results on the two datasets also yielded consistent conclusions, validated by STMicroelectronics process engineers, thereby reinforcing the findings presented in the paper. The agreement of results with two separate methods strengthens the validity of the approach, particularly the choice of making least assumptions on the unavailable data.

Based on the insights gleaned from this study, the industry can focus its investigations on a more limited number of layers, thereby increasing the number of wafers inspected at the second, deeper, and more expensive level of SEM. Establishing a set of layers, albeit more limited, that are consistently or predominantly inspected for each wafer enhances the predictability of models, leading to more accurate estimates of the electrical failures of individual dice and of average yield on a wafer basis.

The prediction models introduced in Section 6.2, Section 6.3, Section 6.4 exhibit poor performance, which we mostly attribute to the insufficient examination of relevant layers. We believe that thoroughly inspecting these layers will lead to significant improvements in the prediction models’ performance.

As a byproduct, several quality indicators were provided that, through with different optimal cutoffs in the prediction models, allow process engineers to select the one that reaches the desired trade-off in terms of Type-I and Type-II errors, contributing to reducing the cost of monitoring the process

### 8.2. Limitations and Future Work

Odds Ratio analysis is computed for each layer or type of defect or their combination separately. While the strength of their association with the outcome of the final electrical test can be estimated, the joint contribution of more layers or more types of defect is hidden. We know that such contribution is present (for example, a defect present in a layer is reasonably present also in subsequent layers, or clusters of type of defects could be present); however, it was studied separately in a suitable model based on graphs (not reported in this study).

Moreover, using only data of each inspected layer separately reduces the size of the sample. Therefore, the power of the test OR = 1 decreases and some significative associations could be hidden by a nonsignificant *p*-value. Also, imbalance of data due to the low rate of failed dice contributes to decreasing the power.

The main limitation of the prediction models is the high number of missing data due to a nonrandom and lacunary selection of the layers to inspect. Even though XGB is able to naturally deal with missing data and therefore mitigate the problem, the accuracy of the models severely decreases and is not acceptable for operative purposes. However, we believe that the model has its own validity.

A general limitation of the study is the assumption that defects are the only source of a failed die, without any other possible independent external cause (see Section 4).

Future research directions include a well-controlled study where inspection by SEM occurs at the same layers (a subset selected based on the Odds Ratio analysis in this study) to create a comprehensive dataset. Additionally, further information from this study, such as the precise position of failures within each die, could be leveraged by the industry to understand the specific reasons behind significant defects.

From a methodological point of view, improvements are to be expected facing the imbalance of data due to the low rate of failed dice in the prediction models. This can be accomplished directly in XGB methodology through suitable cost functions [60,61] or by using advanced tools such as SMOTE.

Another future research direction is the inclusion of information on adjacent dice. As is well known, maps of electrical failures and of defects exhibit spatial patterns that are classified in some defined categories. Therefore, the use of spatial information on dice could enhance the capability to predict failures and yield. However, this extension is challenging from a methodological and computational point of view due to the much higher number of variables involved.

Finally, we believe that a direction of research useful to process engineers is to directly tune the prediction models on the cost of the process for testing the dice, inspecting wafers and discarding failed dice/wafers. This could be made using cost functions in XGB based on such economic costs.

## Figures and Tables

**Table 1:** Datasets ARES and TETIS provided by STMicroelectronics for the study.

	ARES	TETIS
Number of lots	126	120
Total number of wafers	2242	2028
Number of dice	4,075,773	2,283,223
Number of dice per wafer	1818	1126
Number of types of defects	72	75
Number of defects detected	1,002,480 (1 defects every four dice in the average)	448,571 (1 defect every five dice in the average)
Number of defects of type =0	974,092 (97.1% of all defects)	428,590 (95.5% of all defects)
Number of defects of type >0	28,888 (2.9% of all defects)	19,981 (4.4% of all defects)
Number of layers	30	35
Average number of inspected layers per wafer	2.4	2.3
Average number of layers per wafer inspected for specific defects (type >0)	1.4	1.2
Inspected dice (type >0 )	629,195 (15.4% of total dice)	368,148 (16.1% of total dice)
Inspected dice with at least one defect (type >0 )	26,192 (4.2% of inspected dice)	18,699 (5.1% of inspected dice)
Inspected faulty dice (type >0 )	33,041 (5.3% of investigated dice–failure rate)	34,021 (9.2% of investigated dice–failure rate)
Inspected faulty dice with at least one defect (type >0 )	4269 (0.7% of inspected dice; failure rate with respect to inspected dice with at least one defect (type >0 ): 16.3%)	3557 (1.0% of inspected dice; failure rate with respect to inspected dice with at least one defect (type >0 ): 19.0%)
Inspected faulty dice with no defect (type >0 )	28,772 (4.6% of inspected dice; failure rate with respect to inspected dice without defects (type >0 ): 4.8%)	30,464 (8.3% of inspected dice; failure rate with respect to inspected dice without defects (type >0 ): 8.7%)
Inspected functional dice with at least one defect (type >0 )	21,923 (3.5% of inspected dice)	15,142 (4.1% of inspected dice)
Inspected functional dice with no defect (type >0 )	574,231 (91.3% of inspected dice)	318,985 (86.6% of inspected dice)
Inspected dice with at least one defect (type =0 )	836,294 (20.5% of inspected dice)	338,127 (14.8% of inspected dice)
Inspected faulty dice (type =0 )	191,942 (4.7% of investigated dice–failure rate)	207,524 (9.1% of investigated dice–failure rate)
Inspected faulty dice with at least one defect (type =0 )	56,536 (1.4% of inspected dice; failure rate with respect to inspected dice with at least one defect (type =0 ): 6.8%)	44,760 (2.0% of inspected dice; failure rate with respect to inspected dice with at least one defect (type =0 ): 13.2%)
Inspected faulty dice with no defect (type =0 )	135,406 (3.3% of inspected dice; failure rate with respect to inspected dice without defects (type =0 ): 4.2%)	162,764 (7.1% of inspected dice; failure rate with respect to inspected dice without defects (type =0 ): 8.3%)
Inspected functional dice with at least one defect (type =0 )	779,758 (19.1% of inspected dice)	293,367 (12.8% of inspected dice)
Inspected functional dice with no defect (type =0 )	310,4073 (76.2% of inspected dice)	1,782,332 (78.1% of inspected dice)

**Table 2:** Contingency table for computing Odds Ratio.

	Outcome Failed Die ( f=1 )	Outcome Functional Die ( f=0 )
Number of dice with defects detected of a certain type or at a certain layer ( d=1 )	$N_{11}$	$N_{10}$
Number of dice with no defects detected of a certain type or layer ( d=0)	$N_{01}$	$N_{00}$

**Table 3:** Odds Ratio analysis with respect to layers for the dataset ARES (dice investigated for specific defects, Type >0 ).

Layer	Number of Dice	Failure Defects	No Failure Defects	Failure No Defects	No Failure No Defects	Failure Rate (All Dice)	Failure Rate (with Defects)	Odds Ratio	*p*-Value (Adjusted)
1358_STICMP	10,896	98	175	525	10,098	0.057	0.359	10.772	0
7550_L1ETCH	16,335	160	291	644	15,240	0.049	0.355	13.011	0
5356_M610DEV	14,536	134	275	746	13,381	0.061	0.328	8.742	0
7676_L2CUCMP	23,595	221	481	1293	21,600	0.064	0.315	7.677	0
6502_M655DEV	9085	72	172	417	8424	0.054	0.295	8.461	0
8368_L3CUCMP	32,778	257	701	1500	30,320	0.054	0.268	7.412	0
7650_L2ETCH	23,556	187	515	1005	21,849	0.051	0.266	7.896	0
7295_CCBARR	58,080	404	1493	2551	53,632	0.051	0.213	5.69	0
7556_L1CUCMP	67,155	429	1733	2805	62,188	0.048	0.198	5.489	0
1380_RTSAC	23,621	106	456	1125	21,934	0.052	0.189	4.537	0
1684_RRII08	47,216	166	716	2412	43,922	0.055	0.188	4.225	0
7596_V1ETCH	72,480	538	2449	3363	66,130	0.054	0.180	4.321	0
6741_SAUSG	21,804	119	548	1045	20,092	0.053	0.178	4.179	0
7695_V2DEV	12,684	82	415	492	11,695	0.045	0.165	4.702	0
5086_PETCH	27,180	149	830	1186	25,015	0.049	0.152	3.789	0
6270_M605DEV	39,974	176	1019	1913	36,866	0.052	0.147	3.331	0
6681_SALISTRIP	27,180	148	861	1437	24,734	0.058	0.147	2.961	0
1544_DTIPCMP	47,112	218	1283	1946	43,665	0.046	0.145	3.815	0
1560_RTSDW	36,340	141	894	1936	33,369	0.057	0.136	2.721	0
2187_M050DEV	56,327	203	1296	2765	52,063	0.053	0.135	2.951	0
7708_V2ETCH	139,524	726	4654	6582	127,562	0.052	0.135	3.024	0
1278_AAETCH	5436	36	153	213	5034	0.046	0.190	5.574	1.20 × 10^−13^
7537_L1SVIL	3610	16	13	322	3259	0.094	0.552	12.409	7.60 × 10^−10^
1877_M255DEV	7268	17	38	358	6855	0.052	0.309	8.604	1.40 × 10^−9^
1293_STIOX16	3634	30	107	232	3265	0.072	0.219	3.957	2.40 × 10^−8^
7307_CCWCMP	3624	16	58	134	3416	0.041	0.216	7.07	3.70 × 10^−8^
5131_RTASDD	1817	11	38	62	1706	0.04	0.224	8.014	2.20 × 10^−6^
1045_M110DEV	9085	16	83	371	8615	0.043	0.162	4.511	4.90 × 10^−6^
1561_M265DEV	10,902	23	208	521	10,150	0.05	0.100	2.167	0.002
8507_V3RDLBARR	30,855	64	963	1504	28,324	0.051	0.062	1.254	0.095

**Table 4:** Odds Ratio analysis with respect to types of defect for the dataset ARES (dice investigated for specific defects, Type >0 ).

Type of Defect	Number of Dice	Failure Defects	No Failure Defects	Failure No Defects	No Failure No Defects	Failure Rate (All Dice)	Failure Rate (with Defects)	Odds Ratio	*p*-Value (Adjusted)
256	62,9195	1	0	33,040	596,154	0.053	1	–	–
48	629,195	1	0	33,040	596,154	0.053	1	–	–
51	629,195	1	0	33,040	596,154	0.053	1	–	–
52	629,195	1	0	33,040	596,154	0.053	1	–	–
65	629,195	1	0	33,040	596,154	0.053	1	–	–
62	629,195	58	19	32,983	596,135	0.053	0.753	54.852	0
47	629,195	106	64	32,935	596,090	0.053	0.624	29.945	0
15	629,195	37	29	33,004	596,125	0.053	0.561	23.016	0
63	629,195	340	332	32,701	595,822	0.053	0.506	18.659	0
121	629,195	58	58	32,983	596,096	0.053	0.5	18.073	0
164	629,195	27	30	33,014	596,124	0.053	0.474	16.261	0
31	629,195	195	250	32,846	595,904	0.053	0.438	14.154	0
69	629,195	129	199	32,912	595,955	0.053	0.393	11.743	0
64	629,195	61	105	32,980	596,049	0.053	0.367	10.511	0
53	629,195	160	325	32,881	595,829	0.053	0.33	8.926	0
10	629,195	33	68	33,008	596,086	0.053	0.327	8.786	0
80	629,195	352	728	32,689	595,426	0.053	0.326	8.809	0
14	629,195	103	230	32,938	595,924	0.053	0.309	8.109	0
19	629,195	505	1292	32,536	594,862	0.053	0.281	7.148	0
59	629,195	163	508	32,878	595,646	0.053	0.243	5.817	0
11	629,195	1235	3943	31,806	592,211	0.053	0.239	5.832	0
27	629,195	69	320	32,972	595,834	0.053	0.177	3.904	0
81	629,195	134	832	32,907	595,322	0.053	0.139	2.917	0
33	629,195	240	2185	32,801	593,969	0.053	0.099	1.99	0
32	629,195	290	2738	32,751	593,416	0.053	0.096	1.92	0
61	629,195	12	1	33,029	596,153	0.053	0.923	191.472	1.30 × 10^−14^
162	629,195	14	5	33,027	596,149	0.053	0.737	49.526	2.70 × 10^−14^
29	629,195	17	14	33,024	596,140	0.053	0.548	21.875	5.50 × 10^−14^
1	629,195	16	14	33,025	596,140	0.053	0.533	20.6	5.50 × 10^−13^
163	629,195	13	8	33,028	596,146	0.053	0.619	29.106	6.90 × 10^−12^
21	629,195	24	68	33,017	596,086	0.053	0.261	6.4	1.10 × 10^−10^
41	629,195	53	324	32,988	595,830	0.053	0.141	2.962	2.60 × 10^−10^
42	629,195	214	2430	32,827	593,724	0.053	0.081	1.594	1.80 × 10^−9^
28	629,195	10	8	33,031	596,146	0.053	0.556	22.47	9.00 × 10^−9^
85	629,195	7	2	33,034	596,152	0.053	0.778	59.946	6.50 × 10^−8^
82	629,195	31	189	33,010	595,965	0.053	0.141	2.974	1.40 × 10^−6^
56	629,195	173	2074	32,868	594,080	0.053	0.077	1.509	1.60 × 10^−6^
83	629,195	28	162	33,013	595,992	0.053	0.147	3.136	1.80 × 10^−6^
50	629,195	10	20	33,031	596,134	0.053	0.333	9.097	3.10 × 10^−6^
20	629,195	9	24	33,032	596,130	0.053	0.273	6.844	6.30 × 10^−5^
46	629,195	65	773	32,976	595,381	0.053	0.078	1.522	0.003
36	629,195	44	1178	32,997	594,976	0.053	0.036	0.676	0.01
71	629,195	3	5	33,038	596,149	0.053	0.375	11.045	0.01
22	629,195	6	27	33,035	596,127	0.053	0.182	4.094	0.011
77	629,195	3	6	33,038	596,148	0.053	0.333	9.251	0.014
92	629,195	2	2	33,039	596,152	0.053	0.5	18.044	0.021
84	629,195	10	79	33,031	596,075	0.053	0.112	2.317	0.032
115	629,195	2	4	33,039	596,150	0.053	0.333	9.351	0.047
999	629,195	7	53	33,034	596,101	0.053	0.117	2.431	0.06
43	629,195	22	252	33,019	595,902	0.053	0.08	1.586	0.061
159	629,195	24	293	33,017	595,861	0.053	0.076	1.488	0.089
30	629,195	1	1	33,040	596,153	0.053	0.5	18.043	0.118
25	629,195	10	104	33,031	596,050	0.053	0.088	1.761	0.127
72	629,195	1	3	33,040	596,151	0.053	0.25	6.552	0.222
95	629,195	1	3	33,040	596,151	0.053	0.25	6.552	0.222
37	629,195	47	756	32,994	595,398	0.053	0.059	1.126	0.457
136	629,195	2	61	33,039	596,093	0.053	0.032	0.636	0.51
40	629,195	1	31	33,040	596,123	0.053	0.031	0.664	0.672
0	4,075,773	56,536	779,758	135,406	3,104,073	0.047	0.068	1.662	0

**Table 5:** List of predictors used in the prediction models, indicating the type and the number of variables for the datasets ARES and TETIS.

Predictor	Type	ARES	TETIS
Number of defects at each layer	Continuous *^a^*	30	35
Number of defects for each type	Continuous *^a^*	72	75
Number of defects at each layer for each type	Continuous *^a^*	2160	2625
Wafer slot	Categorical	25 *^b^*	25 *^b^*
Distance	Continuous	1 (range [0, 1])	1 (range [0, 1])

*^a^* Actually integer. *^b^* One Hot encoding.

**Table 6:** Best cutoff for each accuracy indicator (rows) and their corresponding values for all best cutoffs (columns). The data pertain to the XGB model with logistic loss and Accuracy as cost and evaluation functions, respectively, applied to the ARES subset including only dice inspected for defects of type >0 .

Indicator	Cutoff	Sensitivity	Specificity	Accuracy	κ Coefficient	*F*-1	*G*-Mean	MCC
Accuracy	0.47	0.130	0.996	0.950	0.200	0.215	0.340	0.270
κ coefficient	0.17	0.264	0.977	0.939	0.282	0.313	0.507	0.287
*F*-1	0.15	0.278	0.973	0.936	0.281	0.313	0.520	0.283
*G*-mean	0.05	0.517	0.785	0.771	0.116	0.192	0.637	0.160
MCC	0.26	0.222	0.973	0.936	0.281	0.301	0.467	0.298

**Table 7:** Accuracy in estimating the yield of a wafer starting from the prediction model die-by-die for the datasets ARES (second and third column) and TETIS (fourth and fifth column). The second and third row show the RMSE and MAE of the models without and with interaction between layers and types of defects. As a reference, the last row indicates the RMSE and the MAE when prediction is the average rate of failure as reported in the first row.

Model	Dataset ARES		Dataset TETIS	
	RMSE	MAE	RMSE	MAE
Rate of failure	0.053		0.092	
Without interaction	0.023	0.018	0.056	0.045
With interaction	0.024	0.018	0.056	0.045
Reference	0.019	0.014	0.033	0.025

## Data Availability

The datasets presented in this article are not available because the property of STMicroelectronics and classified.

## Supplementary Materials

The following supporting information can be downloaded at: https://www.mdpi.com/article/10.3390/s25134218/s1.

## Abbreviations

The following abbreviations are used in this manuscript:

AI	Artificial Intelligence
AUC	Area Under Curve
FN	False Negative
FP	False Positive
GB	Gradient Boosting
MAR	Missing At Random
MCAR	Missing Completely At Random
MCC	Matthews Correlation Coefficient
ROC	Receiving Operating Characteristic
SEM	Scanning Electron Microscope
TN	True Negative
TP	True Positive
XGB	eXtreme Gradient Boosting
