# Correction: Amato et al. Detecting Important Features and Predicting Yield from Defects Detected by SEM in Semiconductor Production. *Sensors* 2025, *25*, 4218

**DOI:** 10.3390/s26165124

**Published:** 2026-08-13

**Authors:** Umberto Amato, Anestis Antoniadis, Italia De Feis, Anastasiia Doinychko, Irène Gijbels, Antonino La Magna, Daniele Pagano, Francesco Piccinini, Easter Selvan Suviseshamuthu, Carlo Severgnini, Andres Torres, Patrizia Vasquez

**Affiliations:** 1 Istituto di Scienze Applicate e Sistemi Intelligenti, National Research Council of Italy, 80131 Napoli, Italy; antoniadis.anestis@na.isasi.cnr.it; 2 Istituto per le Applicazioni del Calcolo ‘Mauro Picone’, National Research Council of Italy, 80131 Napoli, Italy; i.defeis@na.iac.cnr.it; 3 Siemens EDA, Plano, TX 75024, USA; anastasiia.doinychko@siemens.com (A.D.); andres.torres@siemens.com (A.T.); 4 Department of Mathematics, KU Leuven, 3001 Leuven, Belgium; irene.gijbels@kuleuven.be; 5 Istituto per la Microelettronica e Microsistemi, National Research Council of Italy, 95121 Catania, Italy; antonino.lamagna@imm.cnr.it; 6 STMicroelectronics, 95121 Catania, Italy; daniele.pagano@st.com (D.P.); patriziavasquez59@gmail.com (P.V.); 7 STMicroelectronics, 20864 Agrate Brianza, Italy; francesco.piccinini@st.com (F.P.); carlo.severgnini@st.com (C.S.); 8 Kessler Foundation, East Hanover, NJ 07936, USA; eselvan@kesslerfoundation.org

## Affiliation Correction

In the published publication [1], there was an error regarding the affiliation of Patrizia Vasquez. Her affiliation was published as “Institute for the Future of Education, Tecnologico de Monterrey, Monterrey 64740, Nuevo Leon, Mexico” by mistake. The updated affiliation is STMicroelectronics, 95121 Catania, Italy, and she was retired when the paper was published. The university name in Affiliation 4 should be KU Leuven, which has also been updated.

The authors state that the scientific conclusions are unaffected. This correction was approved by the Academic Editor. The original publication has also been updated.
