# Supplementary material for: Detecting Important Features and Predicting Yield from Defects Detected by SEM in Semiconductor Production
Source: Sensors (Basel). 2025 Jul 6;25(13):4218. doi: 10.3390/s25134218 (PMC12252516; doi:10.3390/s25134218)
Supplement: Supplementary file 1 [file sensors-25-04218-s001.zip › sensors-3592627-supplementary.pdf]

Detecting important features and predicting yield from defects detected by SEM in semiconductor production

Umberto Amato
ISASI-CNR, Italy

Anestis Antoniadis
ISASI-CNR, Italy

Italia De Feis
IAC-CNR, Italy

Anastasiia Doinychko
Siemens EDA, USA

Irène Gijbels
University of Leuven, Belgium

Antonino La Magna
IMM-CNR, Italy

Daniele Pagano
STMicroelecrtonics, Italy

Francesco Piccinini
STMicroelecrtonics, Italy

Easter Selvan Suviseshamuthu
Kessler Foundation, USA

Carlo Severgnini
STMicroelecrtonics, Italy

Andres Torres
Siemens EDA, USA

Patrizia Vasquez
Italy

These Supplementary Materials contain analyses that are not essential for the full comprehension of the parent paper but can provide additional insights into certain aspects of the work. Due to space constraints in the parent paper, these Supplementary Materials are included to offer a more comprehensive understanding of specific details.

In particular, Section S1 focuses on full Odds Ratio analysis, discussion for the dataset TETIS and some supplementary tables and figures for the dataset ARES. Subsequently, in Section S2 plots illustrating the distribution of inspected layers are shown in various configurations. Section S3 presents some results of the prediction models developed in Section 6 of the parent paper.

S1 Odds Ratio Analysis

S1.1 Dataset TETIS

S1.1.1 Odds Ratio analysis by layer (dice inspected for generic defects)

Table S1 presents a comprehensive analysis of Odds Ratio by layer for the entire dataset TETIS, where all dice have been inspected for generic defects (type = 0). Similar to the table in the parent paper (Tab. 3 for the dataset ARES), the information provided for each layer includes:

- the number of inspected dice per layer;
- the contingency table used to compute the Odds Ratio, detailing the number of dice indicating electric failure/success and the presence/lack of defects;
- the overall failure rate for all inspected dice at each specific layer;
- the failure rate specifically for the subset showing at least one defect of any type;
- the calculated value of the Odds Ratio;
- the p-value of the test for the null hypothesis $OR = 1$.

If a layer is not predictive of electric failure, we expect that the failure rates for the entire corresponding subset and the subset restricted to dice with at least one detected defect will be similar. Conversely, a higher failure rate in the subset restricted to dice with defects indicates a stronger association between defects and a condition of failed dice, enhancing the predictability of a failure.

Entries in the table are sorted by p-value in decreasing order. Layers without dice showing electric failure and any type of detected defect are excluded as Odds Ratio analysis is not applicable in such cases.

Layer	Number of dice	Failure Defects	No failure Defects	Failure No defects	No failure No defects	Failure rate (all dice)	Failure rate (with defects)	Odds Ratio	p-value (adjusted)
1561_M265DEV	130,266	523	1159	10,820	117,764	0.087	0.311	4.912	0
3177_M015DEV	137,006	642	1432	10,883	124,049	0.084	0.310	5.111	0
3417_M025DEV	147,099	800	1785	13,215	131,299	0.095	0.309	4.453	0
7307_CCWCMP	72,192	1339	3303	5504	62,046	0.095	0.288	4.57	0
7550_L1ETCH	54,048	888	2226	4302	46,632	0.096	0.285	4.324	0
6111_M645DEV	86,471	719	1825	7255	76,672	0.092	0.283	4.164	0
1560_RTDSW	315,549	2138	6348	25,454	281,609	0.087	0.252	3.726	0
1358_STICMP	42,788	865	2768	3410	35,745	0.1	0.238	3.276	0
1293_STIOX16	105,548	401	1390	9412	94,345	0.093	0.224	2.893	0
1380_RTSAC1	154,974	1019	3765	13,882	136,308	0.096	0.213	2.658	0
6741_SAUSG	242,554	3390	12,530	18,291	208,343	0.089	0.213	3.082	0
7596_V1ETCH	68,686	848	3206	5959	58,673	0.099	0.209	2.605	0
3455_RTWELL	161,698	1162	4712	13,889	141,935	0.093	0.198	2.52	0
1278_AAETCH	46,166	655	2881	3789	38,841	0.096	0.185	2.331	0
5086_PETCH	36,032	1265	5712	2600	26,455	0.107	0.181	2.253	0
8368_L3CUCMP	67,668	770	3547	5697	57,654	0.096	0.178	2.197	0
6681_SALISTRIP	42,788	1753	8491	2478	30,066	0.099	0.171	2.505	0
3744_M395DEV	141,484	1168	5861	11,047	123,408	0.086	0.166	2.226	0
5356_M615DEV	170,680	1883	9653	15,769	143,375	0.103	0.163	1.774	0
6502_M657DEV	133,632	1713	8929	10,521	112,469	0.092	0.161	2.051	0
7650_L2ETCH	73,165	1014	5378	6220	60,553	0.099	0.159	1.836	0
3635_POLY2000	425,628	7827	41,538	31,335	344,928	0.092	0.159	2.074	0
7556_L1CUCMP	43,914	1076	5929	3063	33,846	0.094	0.154	2.006	0
1952_RRII01	213,368	2385	13,154	17,194	180,635	0.092	0.153	1.905	0
5131_RTASDD	206,632	2430	13,460	14,662	176,080	0.083	0.153	2.168	0
1192_ETHM583	102,501	701	4167	7721	89,912	0.082	0.144	1.959	0
6270_M607DEV	166,202	2074	13,091	12,443	138,594	0.087	0.137	1.765	0
1684_RRII08	702,574	15,302	104,245	47,690	535,337	0.09	0.128	1.648	0
8024_V3RDLBARR	624,833	938	6706	55,867	561,322	0.091	0.123	1.406	0
7295_CCBARR	181,286	4950	38,225	11,967	126,144	0.093	0.115	1.365	0
7725_PETEOS	97,701	353	2605	7564	87,179	0.081	0.119	1.562	3.10×10^{-13}
7695_V2DEV	13,536	118	734	985	11,699	0.081	0.138	1.911	7.10×10^{-9}
1544_DTIPCMP	42,864	838	6837	3171	32,018	0.094	0.109	1.238	3.50×10^{-7}
7676_L2CUCMP	6756	58	413	442	5843	0.074	0.123	1.86	9.20×10^{-5}
8507_V3RDLBARR	95,710	1706	14,869	7563	71,572	0.097	0.103	1.086	0.004

Table S1: Odds Ratio analysis with respect to layers for the dataset TETIS. For each layer, the table reports the number of inspected dice along with the corresponding contingency table. Additionally, the failure rate is provided for all dice inspected at each layer, both for all dice and those with defects found. The Odds Ratio value, along with the adjusted p-value for the test $OR = 1$, corrected for multiplicity, is presented. Entries are sorted in descending order of p-values. The table is based on the entire dataset (dice inspected for generic defects of type = 0).

We observe that all layers are significant at the significance level 0.01 and that layers from 1561_M265DEV to 3455_RTWELL have a failure rate (with detected defects) higher than the global average.

S1.1.2 Odds Ratio analysis by layer (dice inspected for specific defects)

Layer	Number of dice	Failure Defects	No failure Defects	Failure No defects	No failure No defects	Failure rate (all dice)	Failure rate (with defects)	Odds Ratio	p-value (adjusted)
1380_RTSAC1	5615	50	80	552	4933	0.107	0.385	5.589	0
7550_L1ETCH	4504	71	114	480	3839	0.122	0.384	4.982	0
7307_CCWCMP	4512	101	171	377	3863	0.106	0.371	6.051	0
3417_M025DEV	5615	74	161	573	4807	0.115	0.315	3.859	0
6111_M645DEV	6738	91	208	549	5890	0.095	0.304	4.696	0
6741_SAUSG	40,414	534	1431	3203	35,246	0.092	0.272	4.107	0
7556_L1CUCMP	12,386	140	405	1084	10,757	0.099	0.257	3.432	0
3635_POLY2000	13,512	126	388	1162	11,836	0.095	0.245	3.31	0
5086_PETCH	12,386	161	518	1308	10,399	0.119	0.237	2.472	0
8368_L3CUCMP	10,152	97	313	805	8937	0.089	0.237	3.444	0
6502_M657DEV	24,706	235	962	2181	21,328	0.098	0.196	2.39	0
1684_RRII08	21,394	129	553	1711	19,001	0.086	0.189	2.593	0
6270_M607DEV	28,075	256	1214	2290	24,315	0.091	0.174	2.24	0
5356_M615DEV	35,922	342	1667	2992	30,921	0.093	0.170	2.121	0
3744_M395DEV	25,815	196	1083	2052	22,484	0.087	0.153	1.984	5.90×10^{-15}
1560_RTDSW	8984	66	165	863	7890	0.103	0.286	3.661	1.00×10^{-14}
7295_CCBARR	24,772	211	1178	2099	21,284	0.093	0.152	1.817	1.20×10^{-12}
3177_M015DEV	5615	47	137	470	4961	0.092	0.255	3.627	7.60×10^{-11}
7596_V1ETCH	10,134	110	450	1022	8552	0.112	0.196	2.047	2.80×10^{-9}
1952_RRII01	24,706	183	1101	2146	21,276	0.094	0.143	1.649	1.70×10^{-8}
6681_SALISTRIP	5630	56	265	421	4888	0.085	0.174	2.458	1.60×10^{-7}
7650_L2ETCH	10,109	82	380	929	8718	0.1	0.177	2.028	2.70×10^{-7}
1293_STIOX16	2246	17	59	128	2042	0.065	0.224	4.619	5.70×10^{-6}
1358_STICMP	2252	25	94	169	1964	0.086	0.210	3.101	2.40×10^{-5}
7695_V2DEV	4512	34	195	311	3972	0.076	0.148	2.234	1.90×10^{-4}
1544_DTIPCMP	7896	55	357	588	6896	0.081	0.133	1.811	2.90×10^{-4}
5131_RTASDD	3369	14	64	207	3084	0.066	0.179	3.285	6.70×10^{-4}
7676_L2CUCMP	1126	10	43	62	1011	0.064	0.189	3.825	0.002
8024_V3RDLBARR	45,113	105	810	3735	40,463	0.085	0.115	1.406	0.002
1192_ETHM583	7888	29	157	667	7035	0.088	0.156	1.957	0.003
1278_AAETCH	9008	36	242	894	7836	0.103	0.129	1.309	0.162
7725_PETEOS	15,722	59	526	1294	13,843	0.086	0.101	1.203	0.205
8507_V3RDLBARR	2252	8	69	198	1977	0.091	0.104	1.178	0.676

Table S2: Odds Ratio analysis with respect to layers for the dataset TETIS (dice investigated for specific defects, Type > 0). For each layer, the table reports the number of inspected dice along with the corresponding contingency table. Additionally, the failure rate is provided for all dice inspected at each layer, both for all dice and those with defects found. The Odds Ratio value, along with the adjusted p-value for the test $OR = 1$, corrected for multiplicity, is presented. Entries are sorted in ascending order of p-values.

Table S2 reveals that most layers are predictive: at almost all layers, the presence of defects significantly predicts a failure of the final electric test. However, the degree of prediction strongly depends on the layer. As a guideline, considering that the average rate of failure for the entire dataset TETIS inspected for specific defects by SEM, with any specific defect found, is 19% (as per Tab. 1 of the parent paper), we can infer that the most interesting layers are the ones exhibiting a rate of failure in the presence of defects higher than 19% and a very low p-value. Specifically, these are the entries in Tab. S2 up to 8368_L3CUCMP, including layers 1560_RTDSW and 3177_M015DEV. Nevertheless, there is a high number of dice without defects but with an electric failure (their number is ranked 2nd in the contingency tables).

Comparison with the analogous case of generic defects (type = 0, Tab. S1) shows that significant layers are similar.

S1.1.3 Odds Ratio analysis by type of defect

Table S3 presents a comprehensive analysis of Odds Ratio by type of defect for the entire dataset TETIS.

Type of defect	Number of dice	Failure Defects	No failure Defects	Failure No defects	No failure No defects	Failure rate (all dice)	Failure rate (with defects)	Odds Ratio	p-value (adjusted)
169	368,148	10	0	34,011	334,127	0.092	1	–	–
61	368,148	4	0	34,017	334,127	0.092	1	–	–
4	368,148	1	0	34,020	334,127	0.092	1	–	–
681	368,148	1	0	34,020	334,127	0.092	1	–	–
71	368,148	1	0	34,020	334,127	0.092	1	–	–
15	368,148	21	4	34,000	334,123	0.092	0.84	49.98	0
62	368,148	28	6	33,993	334,121	0.092	0.824	44.918	0
162	368,148	21	5	34,000	334,122	0.092	0.808	40.289	0
92	368,148	25	6	33,996	334,121	0.092	0.806	40.13	0
47	368,148	34	12	33,987	334,115	0.092	0.739	27.609	0
121	368,148	35	31	33,986	334,096	0.092	0.53	11.092	0
31	368,148	85	94	33,936	334,033	0.092	0.475	8.902	0
63	368,148	108	153	33,913	333,974	0.092	0.414	6.955	0
80	368,148	197	297	33,824	333,830	0.092	0.399	6.548	0
27	368,148	117	218	33,904	333,909	0.092	0.349	5.289	0
83	368,148	173	372	33,848	333,755	0.092	0.317	4.588	0
19	368,148	727	1843	33,294	332,284	0.092	0.283	3.937	0
32	368,148	328	1565	33,693	332,562	0.092	0.173	2.07	0
11	368,148	629	3013	33,392	331,114	0.092	0.173	2.07	0
81	368,148	204	1068	33,817	333,059	0.092	0.16	1.882	6.0×10^{-14}
69	368,148	29	33	33,992	334,094	0.092	0.468	8.643	7.3×10^{-14}
10	368,148	33	51	33,988	334,076	0.092	0.393	6.371	5.7×10^{-13}
56	368,148	369	2357	33,652	331,770	0.092	0.135	1.544	7.3×10^{-13}
64	368,148	44	104	33,977	334,023	0.092	0.297	4.168	5.6×10^{-12}
14	368,148	44	153	33,977	333,974	0.092	0.223	2.834	1.2×10^{-7}
52	368,148	8	2	34,013	334,125	0.092	0.8	37.188	5.5×10^{-7}
82	368,148	101	546	33,920	333,581	0.092	0.156	1.822	6.4×10^{-7}
73	368,148	31	94	33,990	334,033	0.092	0.248	3.253	8.9×10^{-7}
24	368,148	10	16	34,011	334,111	0.092	0.385	6.177	1.6×10^{-4}
53	368,148	10	21	34,011	334,106	0.092	0.323	4.718	0.001
42	368,148	158	1143	33,863	332,984	0.092	0.121	1.361	0.001
41	368,148	24	103	33,997	334,024	0.092	0.189	2.301	0.002
33	368,148	139	1033	33,882	333,094	0.092	0.119	1.324	0.006
940	368,148	65	431	33,956	333,696	0.092	0.131	1.485	0.009
815	368,148	3	2	34,018	334,125	0.092	0.6	14.39	0.013
901	368,148	3	2	34,018	334,125	0.092	0.6	14.39	0.013
20	368,148	6	15	34,015	334,112	0.092	0.286	3.992	0.021
21	368,148	8	28	34,013	334,099	0.092	0.222	2.847	0.034
48	368,148	2	1	34,019	334,126	0.092	0.667	18.459	0.041
164	368,148	9	40	34,012	334,087	0.092	0.184	2.241	0.075
43	368,148	2	56	34,019	334,071	0.092	0.034	0.377	0.174
166	368,148	1	1	34,020	334,126	0.092	0.5	9.821	0.28
46	368,148	30	231	33,991	333,896	0.092	0.115	1.282	0.318

Continued on next page

Table S3 – *Continued from previous page*

Type of defect	Number of dice	Failure Defects	No failure Defects	Failure No defects	No failure No defects	Failure rate (all dice)	Failure rate (with defects)	Odds Ratio	p-value (adjusted)
159	368,148	6	34	34,015	334,093	0.092	0.15	1.772	0.333
54	368,148	7	42	34,014	334,085	0.092	0.143	1.669	0.339
35	368,148	1	2	34,020	334,125	0.092	0.333	5.226	0.36
65	368,148	1	2	34,020	334,125	0.092	0.333	5.226	0.36
72	368,148	1	2	34,020	334,125	0.092	0.333	5.226	0.36
904	368,148	20	250	34,001	333,877	0.092	0.074	0.792	0.381
44	368,148	2	9	34,019	334,118	0.092	0.182	2.311	0.429
28	368,148	2	12	34,019	334,115	0.092	0.143	1.741	0.62
36	368,148	4	30	34,017	334,097	0.092	0.118	1.355	0.7
902	368,148	6	49	34,015	334,078	0.092	0.109	1.231	0.749
903	368,148	1	7	34,020	334,120	0.092	0.125	1.572	0.805
999	368,148	2	16	34,019	334,111	0.092	0.111	1.31	0.822
22	368,148	1	9	34,020	334,118	0.092	0.1	1.229	0.887
25	368,148	5	45	34,016	334,082	0.092	0.1	1.123	0.887
29	368,148	1	9	34,020	334,118	0.092	0.1	1.229	0.887
84	368,148	1	9	34,020	334,118	0.092	0.1	1.229	0.887
630	368,148	7	67	34,014	334,060	0.092	0.095	1.047	0.925
59	368,148	6	58	34,015	334,069	0.092	0.094	1.041	0.927
0	2,283,223	44,760	293,367	162,764	1,782,332	0.091	0.132	1.671	0

Table S3: Odds Ratio analysis with respect to types of defect for the dataset TETIS. For each type of defect, the table reports the number of inspected dice along with the corresponding contingency table. Additionally, the failure rate is provided for all dice inspected at each layer, both for all dice and those with defects found. The Odds Ratio value, along with the adjusted p-value for the test $OR = 1$, corrected for multiplicity, is presented. Entries are sorted in ascending order of p-values. The last row (type of defect = 0) includes the entire dataset.

Table S3 shows that the number of predictive types of defect is about one-half of all possible types (39 out of 75 at the 95% confidence level). It also includes types for which only a few dice were found with defects. They were retained despite their ambiguity because in many cases they show a high failure rate (in some cases equal to 1, meaning all dice with at least one defect are faulty), which could be indicators of very specific and selective predictors of a failure, to be investigated by process engineers. In the case when no dice are found with a successful electric test and no detected defects, the Odds Ratio and p-value of the corresponding test $OR = 1$ are not defined. From the last row of Table S3, we notice that even defects of type = 0 are significant, although with a moderate value of the Odds Ratio and of the failure rate in the presence of defects.

S1.1.4 Odds Ratio analysis by layer and type of defect for dice inspected for specific defects

The analyses presented in Tabs. S1 and S3 consider layers and types of defects separately. This means that all detected defects at each layer are included, and all inspected layers are considered for each type of defect, respectively. This approach introduces a degree of weakness into the Odds Ratio analysis, as it combines both predictive and nonpredictive conditions within each Odds Ratio instance. For instance, fixing a layer allows the detection of both predictive and nonpredictive types of defects for electric failure. Conversely, a type of defect might be predictive only when detected at a specific layer, and mixing all layers could obscure its association or predictability capability. Essentially, this approach does not account for the interaction between layers and types of defects, where a specific layer could predict electric failure only when specific types of defects are detected.

To address this limitation, the Odds Ratio Analysis was extended to consider all pairs of layers and types of defects.

Results are presented in tabular form in Tab. S4. The table does not include non-significant cases where the adjusted p-value is > 0.05. First, instances are displayed where no electrically healthy dice with at least one defect exist, making the Odds Ratio undefined (and consequently, the test for the hypothesis $OR = 1$ cannot be applied). These instances, sorted in decreasing order by the number of electrically failed dice with at least one detected defect, serve as a significant indicator. Following this set of cases, pairs of layer and type of defect with a p-value of the test $OR = 1$ smaller than zero machine are added, assimilated to 0. These cases are sorted in decreasing order of the failure rate restricted to dice with at least one defect. Finally, all other pairs are presented, sorted by p-value in decreasing order.

Layer	Type of defect	Number of dice	Failure Defects	No failure Defects	Failure No defects	No failure No defects	Failure rate (all dice)	Failure rate (with defects)	Odds Ratio	p-value (adjusted)
1684_RRII08	15	21,394	13	0	1827	19,554	0.086	1	Inf	NA
1560_RTDSW	169	8984	10	0	919	8055	0.103	1	Inf	NA
6741_SAUSG	162	40,414	10	0	3727	36,677	0.092	1	Inf	NA
1952_RRII01	92	24,706	8	0	2321	22,377	0.094	1	Inf	NA
1952_RRII01	80	24,706	6	0	2323	22,377	0.094	1	Inf	NA
3744_M395DEV	47	25,815	6	0	2242	23,567	0.087	1	Inf	NA
3635_POLY2000	47	13,512	5	0	1283	12,224	0.095	1	Inf	NA
1192_ETHM583	19	7888	4	0	692	7192	0.088	1	Inf	NA
1380_RTSAC1	69	5615	4	0	598	5013	0.107	1	Inf	NA
6741_SAUSG	92	40,414	4	0	3733	36,677	0.092	1	Inf	NA
7556_L1CUCMP	64	12,386	4	0	1220	11,162	0.099	1	Inf	NA
1684_RRII08	47	21,394	3	0	1837	19,554	0.086	1	Inf	NA
3744_M395DEV	162	25,815	3	0	2245	23,567	0.087	1	Inf	NA
5086_PETCH	52	12,386	3	0	1466	10,917	0.119	1	Inf	NA
5356_M615DEV	80	35,922	3	0	3331	32,588	0.093	1	Inf	NA
6741_SAUSG	10	40,414	3	0	3734	36,677	0.092	1	Inf	NA
1380_RTSAC1	47	5615	2	0	600	5013	0.107	1	Inf	NA
3417_M025DEV	47	5615	2	0	645	4968	0.115	1	Inf	NA
3635_POLY2000	10	13,512	2	0	1286	12,224	0.095	1	Inf	NA
5131_RTASDD	15	3369	2	0	219	3148	0.066	1	Inf	NA
6270_M607DEV	15	28,075	2	0	2544	25,529	0.091	1	Inf	NA
6270_M607DEV	162	28,075	2	0	2544	25,529	0.091	1	Inf	NA
6270_M607DEV	62	28,075	2	0	2544	25,529	0.091	1	Inf	NA
6270_M607DEV	92	28,075	2	0	2544	25,529	0.091	1	Inf	NA
6502_M657DEV	15	24,706	2	0	2414	22,290	0.098	1	Inf	NA
6681_SALISTRIP	47	5630	2	0	475	5153	0.085	1	Inf	NA
6741_SAUSG	31	40,414	2	0	3735	36,677	0.092	1	Inf	NA
7550_L1ETCH	62	4504	2	0	549	3953	0.122	1	Inf	NA
7556_L1CUCMP	61	12,386	2	0	1222	11,162	0.099	1	Inf	NA
7596_V1ETCH	69	10,134	2	0	1130	9002	0.112	1	Inf	NA
7676_L2CUCMP	48	1126	2	0	70	1054	0.064	1	Inf	NA
7676_L2CUCMP	62	1126	2	0	70	1054	0.064	1	Inf	NA
1293_STIOX16	63	2246	1	0	144	2101	0.065	1	Inf	NA
1684_RRII08	64	21,394	1	0	1839	19,554	0.086	1	Inf	NA
1952_RRII01	164	24,706	1	0	2328	22,377	0.094	1	Inf	NA
1952_RRII01	4	24,706	1	0	2328	22,377	0.094	1	Inf	NA
3177_M015DEV	121	5615	1	0	516	5098	0.092	1	Inf	NA
3177_M015DEV	33	5615	1	0	516	5098	0.092	1	Inf	NA
3635_POLY2000	121	13,512	1	0	1287	12,224	0.095	1	Inf	NA

Continued on next page

Table S4 – *Continued from previous page*

Layer	Type of defect	Number of dice	Failure Defects	No failure Defects	Failure No defects	No failure No defects	Failure rate (all dice)	Failure rate (with defects)	Odds Ratio	p-value (adjusted)
3635_POLY2000	20	13,512	1	0	1287	12,224	0.095	1	Inf	NA
3635_POLY2000	21	13,512	1	0	1287	12,224	0.095	1	Inf	NA
3744_M395DEV	44	25,815	1	0	2247	23,567	0.087	1	Inf	NA
3744_M395DEV	63	25,815	1	0	2247	23,567	0.087	1	Inf	NA
5086_PETCH	69	12,386	1	0	1468	10,917	0.119	1	Inf	NA
5131_RTASDD	62	3369	1	0	220	3148	0.066	1	Inf	NA
5356_M615DEV	10	35,922	1	0	3333	32,588	0.093	1	Inf	NA
5356_M615DEV	164	35,922	1	0	3333	32,588	0.093	1	Inf	NA
5356_M615DEV	53	35,922	1	0	3333	32,588	0.093	1	Inf	NA
6111_M645DEV	46	6738	1	0	639	6098	0.095	1	Inf	NA
6111_M645DEV	69	6738	1	0	639	6098	0.095	1	Inf	NA
6270_M607DEV	121	28,075	1	0	2545	25,529	0.091	1	Inf	NA
6270_M607DEV	52	28,075	1	0	2545	25,529	0.091	1	Inf	NA
6270_M607DEV	53	28,075	1	0	2545	25,529	0.091	1	Inf	NA
6270_M607DEV	61	28,075	1	0	2545	25,529	0.091	1	Inf	NA
6270_M607DEV	65	28,075	1	0	2545	25,529	0.091	1	Inf	NA
6270_M607DEV	71	28,075	1	0	2545	25,529	0.091	1	Inf	NA
6270_M607DEV	72	28,075	1	0	2545	25,529	0.091	1	Inf	NA
6502_M657DEV	80	24,706	1	0	2415	22,290	0.098	1	Inf	NA
6681_SALISTRIP	52	5630	1	0	476	5153	0.085	1	Inf	NA
6741_SAUSG	52	40,414	1	0	3736	36,677	0.092	1	Inf	NA
6741_SAUSG	61	40,414	1	0	3736	36,677	0.092	1	Inf	NA
7295_CCBARR	21	24,772	1	0	2309	22,462	0.093	1	Inf	NA
7596_V1ETCH	10	10,134	1	0	1131	9002	0.112	1	Inf	NA
7695_V2DEV	14	4512	1	0	344	4167	0.076	1	Inf	NA
7725_PETEOS	121	15,722	1	0	1352	14,369	0.086	1	Inf	NA
8024_V3RDLBARR	681	45,113	1	0	3839	41,273	0.085	1	Inf	NA
8368_L3CUCMP	15	10,152	1	0	901	9250	0.089	1	Inf	NA
8368_L3CUCMP	62	10,152	1	0	901	9250	0.089	1	Inf	NA
8368_L3CUCMP	69	10,152	1	0	901	9250	0.089	1	Inf	NA
1380_RTSAC1	80	5615	36	5	566	5008	0.107	0.878	61.907	0
7295_CCBARR	63	24,772	38	16	2272	22,446	0.093	0.704	23.319	0
7307_CCWCMP	19	4512	60	72	418	3962	0.106	0.455	7.895	0
8368_L3CUCMP	27	10,152	48	58	854	9192	0.089	0.453	8.909	0
3417_M025DEV	19	5615	48	66	599	4902	0.115	0.421	5.954	0
6741_SAUSG	19	40,414	159	240	3578	36,437	0.092	0.398	6.748	0
3635_POLY2000	80	13,512	56	104	1232	12,120	0.095	0.35	5.303	0
6111_M645DEV	19	6738	54	103	586	5995	0.095	0.344	5.368	0
6741_SAUSG	83	40,414	173	372	3564	36,305	0.092	0.317	4.739	0
7556_L1CUCMP	27	12,386	68	159	1156	11,003	0.099	0.3	4.075	0
5356_M615DEV	19	35,922	139	472	3195	32,116	0.093	0.227	2.962	0
7550_L1ETCH	11	4504	43	55	508	3898	0.122	0.439	6	9.80×10^{-14}
1684_RRII08	80	21,394	43	93	1797	19,461	0.086	0.316	5.017	6.60×10^{-13}
6502_M657DEV	19	24,706	86	285	2330	22,005	0.098	0.232	2.853	7.80×10^{-13}
6741_SAUSG	11	40,414	44	97	3693	36,580	0.092	0.312	4.502	7.10×10^{-12}
7295_CCBARR	11	24,772	76	270	2234	22,192	0.093	0.22	2.8	3.70×10^{-11}
6270_M607DEV	19	28,075	68	239	2478	25,290	0.091	0.221	2.909	1.10×10^{-10}
5356_M615DEV	32	35,922	55	166	3279	32,422	0.093	0.249	3.282	2.40×10^{-10}
1952_RRII01	63	24,706	18	13	2311	22,364	0.094	0.581	13.35	4.60×10^{-10}

Continued on next page

Table S4 – *Continued from previous page*

Layer	Type of defect	Number of dice	Failure Defects	No failure Defects	Failure No defects	No failure No defects	Failure rate (all dice)	Failure rate (with defects)	Odds Ratio	p-value (adjusted)
6741_SAUSG	81	40,414	106	495	3631	36,182	0.092	0.176	2.136	2.20×10^{-9}
6502_M657DEV	31	24,706	14	6	2402	22,284	0.098	0.7	21.317	3.10×10^{-9}
3177_M015DEV	19	5615	32	69	485	5029	0.092	0.317	4.819	3.10×10^{-9}
6270_M607DEV	32	28,075	92	421	2454	25,108	0.091	0.179	2.239	4.40×10^{-9}
7650_L2ETCH	19	10,109	18	20	993	9078	0.1	0.474	8.232	7.90×10^{-8}
1560_RTDSW	10	8984	14	9	915	8046	0.103	0.609	13.584	8.50×10^{-8}
3744_M395DEV	80	25,815	10	3	2238	23,564	0.087	0.769	33.859	8.90×10^{-8}
7307_CCWCMP	121	4512	11	5	467	4029	0.106	0.688	18.635	7.20×10^{-7}
7556_L1CUCMP	11	12,386	28	65	1196	11,097	0.099	0.301	4.009	7.80×10^{-7}
6111_M645DEV	14	6738	16	21	624	6077	0.095	0.432	7.433	1.10×10^{-6}
5086_PETCH	31	12,386	8	1	1461	10,916	0.119	0.889	53.159	4.20×10^{-6}
5086_PETCH	62	12,386	8	1	1461	10,916	0.119	0.889	53.159	4.20×10^{-6}
6502_M657DEV	11	24,706	68	301	2348	21,989	0.098	0.184	2.119	4.30×10^{-6}
6270_M607DEV	31	28,075	14	19	2532	25,510	0.091	0.424	7.445	5.30×10^{-6}
6741_SAUSG	56	40,414	56	244	3681	36,433	0.092	0.187	2.277	5.30×10^{-6}
6741_SAUSG	63	40,414	8	3	3729	36,674	0.092	0.727	25.405	8.20×10^{-6}
7307_CCWCMP	11	4512	23	50	455	3984	0.106	0.315	4.041	1.10×10^{-5}
3744_M395DEV	31	25,815	10	9	2238	23,558	0.087	0.526	11.673	1.30×10^{-5}
5356_M615DEV	31	35,922	11	12	3323	32,576	0.093	0.478	8.996	2.40×10^{-5}
5086_PETCH	11	12,386	25	53	1444	10,864	0.119	0.321	3.56	2.40×10^{-5}
7596_V1ETCH	11	10,134	30	79	1102	8923	0.112	0.275	3.085	2.40×10^{-5}
1278_AAETCH	80	9008	12	15	918	8063	0.103	0.444	7.042	6.10×10^{-5}
1684_RRII08	82	21,394	22	68	1818	19,486	0.086	0.244	3.485	6.90×10^{-5}
7307_CCWCMP	14	4512	14	22	464	4012	0.106	0.389	5.521	8.60×10^{-5}
3744_M395DEV	19	25,815	25	86	2223	23,481	0.087	0.225	3.085	9.70×10^{-5}
3635_POLY2000	11	13,512	13	22	1275	12,202	0.095	0.371	5.683	1.10×10^{-4}
6270_M607DEV	47	28,075	6	2	2540	25,527	0.091	0.75	28.714	1.20×10^{-4}
1544_DTIPCMP	82	7896	17	48	626	7205	0.081	0.262	4.1	1.20×10^{-4}
6502_M657DEV	32	24,706	38	151	2378	22,139	0.098	0.201	2.35	1.60×10^{-4}
7596_V1ETCH	19	10,134	11	13	1121	8989	0.112	0.458	6.798	1.90×10^{-4}
3744_M395DEV	92	25,815	5	1	2243	23,566	0.087	0.833	47.268	2.30×10^{-4}
6111_M645DEV	31	6738	7	5	633	6093	0.095	0.583	13.346	3.00×10^{-4}
7556_L1CUCMP	62	12,386	5	1	1219	11,161	0.099	0.833	41.181	4.10×10^{-4}
8368_L3CUCMP	80	10,152	6	4	896	9246	0.089	0.6	15.273	6.00×10^{-4}
5086_PETCH	33	12,386	26	74	1443	10,843	0.119	0.26	2.651	7.70×10^{-4}
7596_V1ETCH	31	10,134	7	5	1125	8997	0.112	0.583	11.092	8.10×10^{-4}
7307_CCWCMP	24	4512	10	15	468	4019	0.106	0.4	5.751	1.00×10^{-3}
3635_POLY2000	56	13,512	30	123	1258	12,101	0.095	0.196	2.356	1.00×10^{-3}
7550_L1ETCH	80	4504	5	1	546	3952	0.122	0.833	32.533	1.00×10^{-3}
5356_M615DEV	73	35,922	14	37	3320	32,551	0.093	0.275	3.736	0.001
3744_M395DEV	69	25,815	4	1	2244	23,566	0.087	0.8	38.083	0.002
1358_STICMP	81	2252	11	28	183	2030	0.086	0.282	4.39	0.002
6270_M607DEV	69	28,075	7	10	2539	25,519	0.091	0.412	7.083	0.003
6111_M645DEV	32	6738	8	13	632	6085	0.095	0.381	5.967	0.003
7695_V2DEV	11	4512	19	88	326	4079	0.076	0.178	2.718	0.003
8024_V3RDLBARR	940	45,113	65	431	3775	40,842	0.085	0.131	1.635	0.003
3744_M395DEV	32	25,815	41	232	2207	23,335	0.087	0.15	1.875	0.004
6681_SALISTRIP	62	5630	4	2	473	5151	0.085	0.667	20.988	0.004
1293_STIOX16	81	2246	10	37	135	2064	0.065	0.213	4.173	0.004

Continued on next page

Layer	Type of defect	Number of dice	Failure Defects	No failure Defects	Failure No defects	No failure No defects	Failure rate (all dice)	Failure rate (with defects)	Odds Ratio	p-value (adjusted)
3177_M015DEV	31	5615	4	2	513	5096	0.092	0.667	19.147	0.006
5356_M615DEV	47	35,922	4	2	3330	32,586	0.093	0.667	18.879	0.006
8368_L3CUCMP	19	10,152	5	5	897	9245	0.089	0.5	10.301	0.006
7556_L1CUCMP	19	12,386	7	11	1217	11,151	0.099	0.389	5.877	0.007
7295_CCBARR	32	24,772	7	12	2303	22,450	0.093	0.368	5.74	0.007
6270_M607DEV	56	28,075	11	32	2535	25,497	0.091	0.256	3.491	0.009
7307_CCWCMP	63	4512	4	2	474	4032	0.106	0.667	16.394	0.009
1380_RTSAC1	19	5615	4	2	598	5011	0.107	0.667	16.154	0.009
5086_PETCH	64	12,386	9	16	1460	10,901	0.119	0.36	4.232	0.01
6270_M607DEV	10	28,075	4	3	2542	25,526	0.091	0.571	13.222	0.01
1358_STICMP	64	2252	3	1	191	2057	0.086	0.75	29.562	0.012
1560_RTDSW	56	8984	16	55	913	8000	0.103	0.225	2.567	0.014
6681_SALISTRIP	121	5630	4	4	473	5149	0.085	0.5	10.875	0.015
3417_M025DEV	11	5615	15	44	632	4924	0.115	0.254	2.674	0.015
8368_L3CUCMP	63	10,152	16	68	886	9182	0.089	0.19	2.457	0.018
1278_AAETCH	33	9008	1	70	929	8008	0.103	0.014	0.141	0.021
1293_STIOX16	69	2246	3	3	142	2098	0.065	0.5	14.727	0.023
1560_RTDSW	11	8984	9	23	920	8032	0.103	0.281	3.453	0.024
7596_V1ETCH	121	10,134	3	1	1129	9001	0.112	0.75	21.944	0.025
5086_PETCH	121	12,386	7	12	1462	10,905	0.119	0.368	4.391	0.025
7650_L2ETCH	31	10,109	4	4	1007	9094	0.1	0.5	9.027	0.025
8024_V3RDLBARR	815	45,113	3	2	3837	41,271	0.085	0.6	15.757	0.025
8024_V3RDLBARR	901	45,113	3	2	3837	41,271	0.085	0.6	15.757	0.025
7550_L1ETCH	64	4504	11	27	540	3926	0.122	0.289	2.987	0.026
6681_SALISTRIP	32	5630	20	106	457	5047	0.085	0.159	2.097	0.027
1560_RTDSW	80	8984	6	11	923	8044	0.103	0.353	4.809	0.028
6502_M657DEV	41	24,706	5	8	2411	22,282	0.098	0.385	5.843	0.029
1293_STIOX16	56	2246	3	4	142	2097	0.065	0.429	11.181	0.035
3417_M025DEV	42	5615	7	14	640	4954	0.115	0.333	3.913	0.036
3417_M025DEV	41	5615	5	7	642	4961	0.115	0.417	5.566	0.038
5086_PETCH	32	12,386	31	134	1438	10,783	0.119	0.188	1.742	0.039
5356_M615DEV	56	35,922	12	48	3322	32,540	0.093	0.2	2.474	0.046
3744_M395DEV	121	25,815	3	3	2245	23,564	0.087	0.5	10.494	0.047
8368_L3CUCMP	11	10,152	7	21	895	9229	0.089	0.25	3.489	0.048
1192_ETHM583	11	7888	6	16	690	7176	0.088	0.273	3.964	0.048
6741_SAUSG	73	40,414	7	20	3730	36,657	0.092	0.259	3.491	0.048
1684_RRII08	63	21,394	4	7	1836	19,547	0.086	0.364	6.184	0.048
1544_DTIPCMP	53	7896	6	18	637	7235	0.081	0.25	3.852	0.05

Table S4: Odds Ratio analysis with respect to layers and types of defect for the dataset TETIS. For each layer and type of defect, the table reports the number of inspected dice along with the corresponding contingency table. Additionally, the failure rate is provided for all dice inspected at each layer, both for all dice and those with defects found. The Odds Ratio value, along with the adjusted p-value for the test $OR = 1$, corrected for multiplicity, is presented. Entries are sorted in descending order of p-values. The table is based on the entire dataset (dice inspected for generic defects of type = 0).

This table can be valuable for process engineers, as the results presented separately for layers and types of defect in Tabs. S1 and S3 might obscure crucial predictability of specific defects at specific layers, as discussed earlier.

Figure S1 illustrates the Odds Ratio analysis through a matrix in graphical form. Rows correspond to layers, and columns represent the types of defect. Each pair is colored based on the p-value of the corresponding test for $OR = 1$, as indicated in the legend. Black cells indicate undefined values of Odds Ratio, primarily due to a lack of useful data, such as the absence of defects.

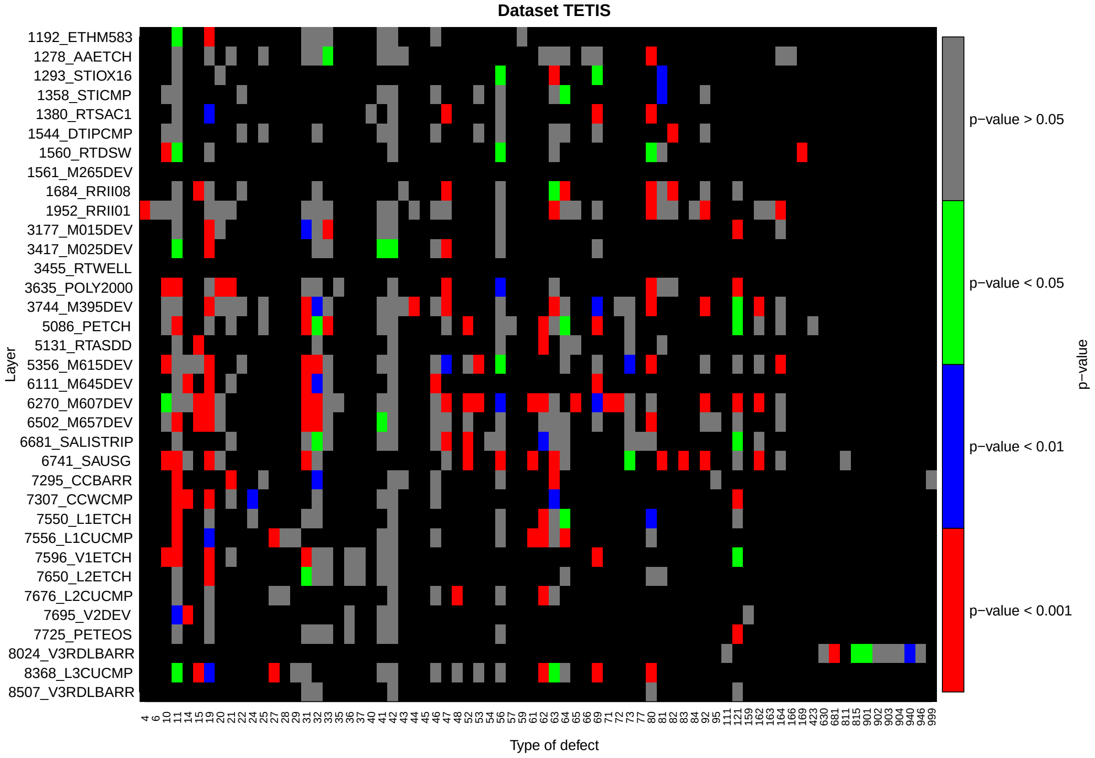

Figure S1: Matrix plot depicting the significance of the test $OR = 1$ based on layers (rows) and types of defects (columns) for the dataset TETIS. Color representation is assigned according to the p-value of the test: red for p-value ≤ 0.001, blue for $0.001 <$ p-value ≤ 0.01, green for $0.01 <$ p-value ≤ 0.05, and gray for p-value > 0.05 (not significant). The background in black indicates an undefined test.

Several features can be observed from the figure: the lack of significance for defect types from 163 onwards (except for one layer at most); a prominent vertical structure of significances, indicating the propagation of defects across consecutive layers; and the identification of the most significant types of defects. For a clearer understanding of the most significant layers and types of defects, Figs. S2 and S3 present the distribution of the number of significances detected for layers and types of defects, respectively, through the Odds Ratio analysis on the pairs of layer and type of defect (significance level 0.001).

The comparison of Figs. S2 and S3 with Tabs. S2 and S3, respectively, reveals a consistency between the most significant layers and types of defects identified through the separate Odds Ratio analysis for layers and types of defects and their interaction.

Nevertheless, the comprehensive table contains richer information valuable to process engineers in identifying instances of defect types at specific layers with high predictability for die failure.

S1.2 Dataset ARES

The dataset for the Odds Ratio analysis comprises 629,195 dice, among which 33,041 exhibited a failed final electric test (5.3%). This failure rate is comparable to that of the entire dataset. Therefore, it can be inferred that the selection of wafers for investigation of specific defects is not made with a targeted approach but is instead compatible with a random one, or the selection criterion is not effectively distinguishing failure-prone wafers. Various subsets can be considered:

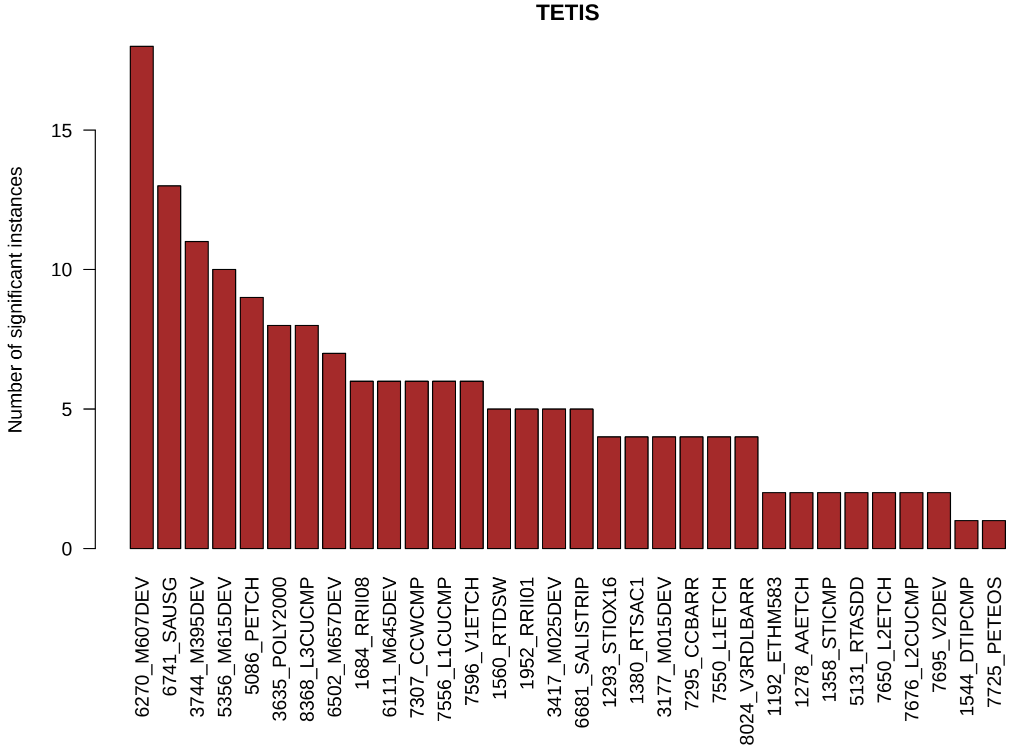

Figure S2: Bar chart illustrating the number of significances by layer, derived from the Odds Ratio analysis on pairs of layers and types of defects for the dataset TETIS (significance level 0.001).

a) The dice with a successful final electric test and no detected specific defects of type > 0 total 574,231 dice, making up 91.3% of all inspected ones (a fraction comparable to the dataset TETIS).

b) The dice with a failed electric test and at least one detected specific defect of type > 0 amount to 4,269, constituting 16.3% of the inspected dice having at least a specific defect of type > 0. In practice, this is the rate of failure of the subset of dice having at least one specific defect of type > 0, which is much higher than the overall rate for the inspected dice (4.2%).

c) The dice with a successful final electric test but with at least one detected defect of type > 0) amount to 21,923, representing 3.5% of the inspected dice, a similar fraction as observed in the dataset TETIS.

d) The electrically failed dice without any detected defect of any type amount to 28,772, constituting 4.6% of the inspected dice, a fraction almost half that of the dataset TETIS. The rate of failure in this subset is 4.8%, once again similar to the rate observed in the entire inspected set.

Subsets a) and b) directly correlate the presence or absence of electric failures with the presence or absence of defects, respectively, encompassing 92.0% of the entire inspected set.

S1.2.1 Odds Ratio analysis by layer for dice inspected for generic defects

Table S5 shown the full analysis of Odds Ratio with respect to layer in the case of dice inspected for generic defects (type $= 0$).

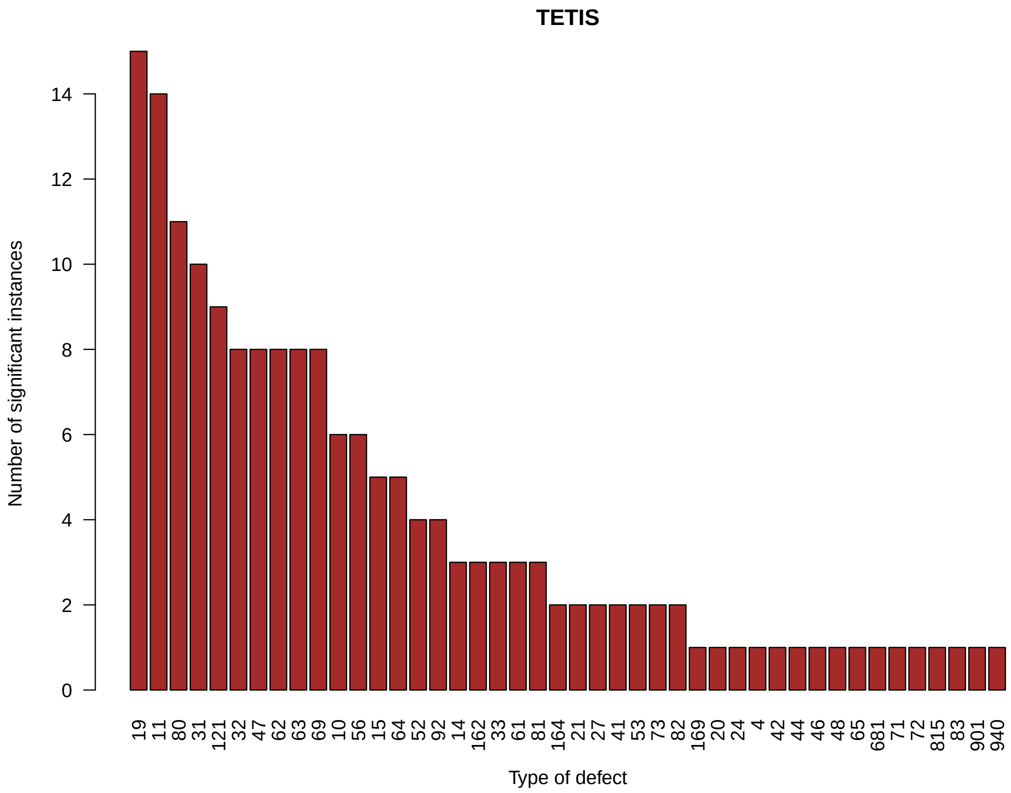

Figure S3: Bar chart illustrating the number of significances by type of defect, derived from the Odds Ratio analysis on pairs of layers and types of defects for the dataset TETIS (significance level 0.001).

Layer	Number of dice	Failure Defects	No failure Defects	Failure No defects	No failure No defects	Failure rate (all dice)	Failure rate (with defects)	Odds Ratio	p-value (adjusted)
7550_L1ETCH	141,570	1389	3528	5322	131,331	0.047	0.282	9.716	0
1293_STIOX16	303,439	756	2098	12,034	288,551	0.042	0.265	8.641	0
7537_L1SVIL	45,125	382	1069	2214	41,460	0.058	0.263	6.693	0
7307_CCWCMP	173,952	1691	4877	6468	160,916	0.047	0.257	8.626	0
1877_M255DEV	352,498	691	2277	15,641	333,889	0.046	0.233	6.479	0
1045_M110DEV	317,975	469	1781	14,319	301,406	0.047	0.208	5.544	0
8368_L3CUCMP	132,933	1412	5414	5290	120,817	0.05	0.207	5.957	0
7676_L2CUCMP	141,570	1238	4837	6089	129,406	0.052	0.204	5.44	0
5356_M610DEV	272,550	808	3180	11,897	256,665	0.047	0.203	5.482	0
1561_M265DEV	405,188	489	2121	18,880	383,698	0.048	0.187	4.687	0
1278_AAETCH	92,412	719	3250	3678	84,765	0.048	0.181	5.099	0
7650_L2ETCH	137,712	1096	5039	5382	126,195	0.047	0.179	5.1	0
7596_V1ETCH	161,268	1023	4770	7110	148,365	0.05	0.177	4.476	0
6502_M655DEV	212,586	737	3439	8293	200,117	0.042	0.176	5.172	0
1380_RTSAC	872,160	2317	11,597	37232	821,014	0.045	0.167	4.406	0
1358_STICMP	150,728	1669	8559	5323	135,177	0.046	0.163	4.952	0
7708_V2ETCH	235,560	2789	14,715	9158	208,898	0.051	0.159	4.323	0
1560_RTSDW	839,454	2454	13,949	34,597	788,454	0.044	0.150	4.01	0
5131_RTASDD	412,459	1196	6922	18,006	386,335	0.047	0.147	3.708	0
6741_SAUSG	168,981	741	4645	6945	156,650	0.045	0.138	3.599	0

Continued on next page

Table S5 – *Continued from previous page*

Layer	Number of dice	Failure Defects	No failure Defects	Failure No defects	No failure No defects	Failure rate (all dice)	Failure rate (with defects)	Odds Ratio	p-value (adjusted)
7556_L1CUCMP	152,460	2749	19,424	4517	125,770	0.048	0.124	3.941	0
2187_M050DEV	381,570	876	6873	16,958	356,863	0.047	0.113	2.683	0
6270_M605DEV	270,733	1073	8608	11,869	249,183	0.048	0.111	2.617	0
7295_CCBARR	313,995	3600	29,256	11,202	269,937	0.047	0.110	2.965	0
7695_V2DEV	27,180	380	3609	877	22,314	0.046	0.095	2.679	0
1544_DTIPCMP	166,704	2308	28,286	5643	130,467	0.048	0.075	1.887	0
5086_PETCH	154,020	3629	44,600	4171	101,620	0.051	0.075	1.982	0
6681_SALISTRIP	170,328	4688	62,971	3752	98,917	0.05	0.069	1.963	0
1684_RRII08 2	261,064	34,713	543,537	73,520	1609294	0.048	0.060	1.398	0
8507_V3RDLBARR	292,215	2195	37,877	12,658	239,485	0.051	0.055	1.096	1.20×10^{-4}

Table S5: Odds Ratio analysis with respect to layers for the dataset ARES. For each layer, the table reports the number of inspected dice along with the corresponding contingency table. Additionally, the failure rate is provided for all dice inspected at each layer, both for all dice and those with defects found. The Odds Ratio value, along with the adjusted p-value for the test $OR = 1$, corrected for multiplicity, is presented. Entries are sorted in descending order of p-values. The table is based on the entire dataset (dice inspected for generic defects of type = 0).

Comparison of Tabs. S5 and 3 of the companion paper reveals a good agreement of results between the datasets inspected for specific (type > 0) and generic (type = 0) defects, respectively, with the same layers being significant. However, as observed with the dataset TETIS, the failure rate when defects are detected is lower for generic defects.

S1.2.2 Odds Ratio analysis by layer and type of defect

Results are presented in tabular form in Tab. S6.

Layer	Type of defect	Number of dice	Failure Defects	No failure Defects	Failure No defects	No failure No defects	Failure rate (all dice)	Failure rate (with defects)	Odds Ratio	p-value (adjusted)
7650_L2ETCH	69	23,556	16	0	1176	22,364	0.051	1	–	–
5086_PETCH	14	27,180	15	0	1320	25,845	0.049	1	–	–
1560_RTSDW	47	36,340	8	0	2069	34,263	0.057	1	–	–
7556_L1CUCMP	61	67,155	7	0	3227	63,921	0.048	1	–	–
5086_PETCH	64	27,180	5	0	1330	25,845	0.049	1	–	–
1684_RRII08	47	47,216	4	0	2574	44,638	0.055	1	–	–
7676_L2CUCMP	61	23,595	4	0	1510	22,081	0.064	1	–	–
7695_V2DEV	69	12,684	4	0	570	12,110	0.045	1	–	–
1380_RTSAC	162	23,621	3	0	1228	22,390	0.052	1	–	–
5086_PETCH	162	27,180	3	0	1332	25,845	0.049	1	–	–
5086_PETCH	31	27,180	3	0	1332	25,845	0.049	1	–	–
5356_M610DEV	47	14,536	3	0	877	13,656	0.061	1	–	–
7708_V2ETCH	10	139,524	3	0	7305	132,216	0.052	1	–	–
1278_AAETCH	15	5436	2	0	247	5187	0.046	1	–	–

Continued on next page

Layer	Type of defect	Number of dice	Failure Defects	No failure Defects	Failure No defects	No failure No defects	Failure rate (all dice)	Failure rate (with defects)	Odds Ratio	p-value (adjusted)
1278_AAETCH	62	5436	2	0	247	5187	0.046	1	–	–
1380_RTSAC	164	23,621	2	0	1229	22,390	0.052	1	–	–
5086_PETCH	62	27,180	2	0	1333	25,845	0.049	1	–	–
5356_M610DEV	80	14,536	2	0	878	13,656	0.061	1	–	–
6270_M605DEV	15	39,974	2	0	2087	37,885	0.052	1	–	–
6681_SALISTRIP	31	27,180	2	0	1583	25,595	0.058	1	–	–
6741_SAUSG	162	21,804	2	0	1162	20,640	0.053	1	–	–
7550_L1ETCH	15	16,335	2	0	802	15,531	0.049	1	–	–
7556_L1CUCMP	71	67,155	2	0	3232	63,921	0.048	1	–	–
1045_M110DEV	63	9085	1	0	386	8698	0.043	1	–	–
1293_STIOX16	63	3634	1	0	261	3372	0.072	1	–	–
1358_STICMP	121	10,896	1	0	622	10,273	0.057	1	–	–
1358_STICMP	22	10,896	1	0	622	10,273	0.057	1	–	–
1358_STICMP	62	10,896	1	0	622	10,273	0.057	1	–	–
1380_RTSAC	41	23,621	1	0	1230	22,390	0.052	1	–	–
1380_RTSAC	71	23,621	1	0	1230	22,390	0.052	1	–	–
1544_DTIPCMP	31	47,112	1	0	2163	44,948	0.046	1	–	–
1560_RTSDW	15	36,340	1	0	2076	34,263	0.057	1	–	–
1560_RTSDW	65	36,340	1	0	2076	34,263	0.057	1	–	–
1684_RRII08	162	47,216	1	0	2577	44,638	0.055	1	–	–
1684_RRII08	22	47,216	1	0	2577	44,638	0.055	1	–	–
1684_RRII08	92	47,216	1	0	2577	44,638	0.055	1	–	–
1877_M255DEV	162	7268	1	0	374	6893	0.052	1	–	–
2187_M050DEV	51	56,327	1	0	2967	53,359	0.053	1	–	–
5086_PETCH	20	27,180	1	0	1334	25,845	0.049	1	–	–
5086_PETCH	63	27,180	1	0	1334	25,845	0.049	1	–	–
6270_M605DEV	10	39,974	1	0	2088	37,885	0.052	1	–	–
6270_M605DEV	164	39,974	1	0	2088	37,885	0.052	1	–	–
6270_M605DEV	52	39,974	1	0	2088	37,885	0.052	1	–	–
6270_M605DEV	62	39,974	1	0	2088	37,885	0.052	1	–	–
6502_M655DEV	121	9085	1	0	488	8596	0.054	1	–	–
6502_M655DEV	30	9085	1	0	488	8596	0.054	1	–	–
6502_M655DEV	62	9085	1	0	488	8596	0.054	1	–	–
6502_M655DEV	63	9085	1	0	488	8596	0.054	1	–	–
6502_M655DEV	64	9085	1	0	488	8596	0.054	1	–	–
6502_M655DEV	69	9085	1	0	488	8596	0.054	1	–	–
6681_SALISTRIP	15	27,180	1	0	1584	25,595	0.058	1	–	–
6681_SALISTRIP	164	27,180	1	0	1584	25,595	0.058	1	–	–
6681_SALISTRIP	61	27,180	1	0	1584	25,595	0.058	1	–	–
6741_SAUSG	20	21,804	1	0	1163	20,640	0.053	1	–	–
7307_CCWCMP	63	3624	1	0	149	3474	0.041	1	–	–
7550_L1ETCH	256	16,335	1	0	803	15,531	0.049	1	–	–
7550_L1ETCH	69	16,335	1	0	803	15,531	0.049	1	–	–
7556_L1CUCMP	10	67,155	1	0	3233	63,921	0.048	1	–	–
7596_V1ETCH	27	72,480	1	0	3900	68,579	0.054	1	–	–
7676_L2CUCMP	47	23,595	1	0	1513	22,081	0.064	1	–	–
7676_L2CUCMP	48	23,595	1	0	1513	22,081	0.064	1	–	–
7676_L2CUCMP	92	23,595	1	0	1513	22,081	0.064	1	–	–
7708_V2ETCH	56	139,524	1	0	7307	132,216	0.052	1	–	–

Continued on next page

Table S6 – *Continued from previous page*

Layer	Type of defect	Number of dice	Failure Defects	No failure Defects	Failure No defects	No failure No defects	Failure rate (all dice)	Failure rate (with defects)	Odds Ratio	p-value (adjusted)
8507_V3RDLBARR	121	30,855	1	0	1567	29,287	0.051	1	–	–
7708_V2ETCH	63	139,524	47	2	7261	132,214	0.052	0.959	398.289	0
7676_L2CUCMP	62	23,595	20	2	1494	22,079	0.064	0.909	138.14	0
7556_L1CUCMP	15	67,155	18	2	3216	63,919	0.048	0.9	167.398	0
7295_CCBARR	63	58,080	122	14	2833	55,111	0.051	0.897	167.729	0
7556_L1CUCMP	62	67,155	15	2	3219	63,919	0.048	0.882	139.618	0
6681_SALISTRIP	47	27,180	43	8	1542	25,587	0.058	0.843	87.694	0
7556_L1CUCMP	69	67,155	25	7	3209	63,914	0.048	0.781	69.953	0
6502_M655DEV	11	9085	41	12	448	8584	0.054	0.774	64.745	0
1358_STICMP	80	10,896	31	13	592	10,260	0.057	0.705	40.986	0
1358_STICMP	47	10,896	20	11	603	10,262	0.057	0.645	30.709	0
7550_L1ETCH	63	16,335	30	17	774	15,514	0.049	0.638	35.199	0
7596_V1ETCH	31	72,480	46	36	3855	68,543	0.054	0.561	22.694	0
7708_V2ETCH	31	139,524	46	39	7262	132,177	0.052	0.541	21.453	0
8368_L3CUCMP	11	32,778	132	153	1625	30,868	0.054	0.463	16.387	0
7676_L2CUCMP	63	23,595	41	58	1473	22,023	0.064	0.414	10.579	0
5356_M610DEV	11	14,536	43	61	837	13,595	0.061	0.413	11.457	0
5086_PETCH	11	27,180	38	54	1297	25,791	0.049	0.413	14.007	0
7650_L2ETCH	32	23,556	27	39	1165	22,325	0.051	0.409	13.287	0
7550_L1ETCH	19	16,335	42	61	762	15,470	0.049	0.408	13.988	0
1684_RRII08	80	47,216	59	92	2519	44,546	0.055	0.391	11.35	0
7596_V1ETCH	19	72,480	44	72	3857	68,507	0.054	0.379	10.869	0
7550_L1ETCH	11	16,335	26	44	778	15,487	0.049	0.371	11.787	0
5356_M610DEV	19	145,36	73	129	807	13,527	0.061	0.361	9.491	0
1544_DTIPCMP	53	47,112	131	233	2033	44,715	0.046	0.36	12.371	0
6741_SAUSG	19	21,804	39	77	1125	20,563	0.053	0.336	9.274	0
7596_V1ETCH	80	72,480	130	259	3771	68,320	0.054	0.334	9.099	0
7556_L1CUCMP	63	67,155	55	114	3179	63,807	0.048	0.325	9.698	0
7295_CCBARR	11	58,080	250	564	2705	54,561	0.051	0.307	8.943	0
7708_V2ETCH	11	139,524	183	435	7125	131,781	0.052	0.296	7.785	0
7596_V1ETCH	11	72,480	77	217	3824	68,362	0.054	0.262	6.352	0
7676_L2CUCMP	11	23,595	75	225	1439	21,856	0.064	0.25	5.069	0
7708_V2ETCH	80	139,524	81	244	7227	131,972	0.052	0.249	6.07	0
2187_M050DEV	19	56,327	57	173	2911	53,186	0.053	0.248	6.031	0
7650_L2ETCH	11	23,556	52	160	1140	22,204	0.051	0.245	6.342	0
7708_V2ETCH	59	139,524	99	307	7209	131,909	0.052	0.244	5.907	0
7556_L1CUCMP	11	67,155	200	652	3034	63,269	0.048	0.235	6.4	0
6270_M605DEV	19	39,974	88	340	2001	37,545	0.052	0.206	4.863	0
7650_L2ETCH	31	23,556	21	24	1171	22,340	0.051	0.467	16.703	4.90×10^{-15}
7708_V2ETCH	19	139,524	43	147	7265	132,069	0.052	0.226	5.332	4.90×10^{-15}
7596_V1ETCH	63	72,480	14	3	3887	68,576	0.054	0.824	79.075	9.40×10^{-15}
7596_V1ETCH	33	72,480	72	390	3829	68,189	0.054	0.156	3.294	9.40×10^{-15}
8368_L3CUCMP	47	32,778	15	7	1742	31,014	0.054	0.682	37.676	1.00×10^{-13}
7695_V2DEV	11	12,684	59	373	515	11,737	0.045	0.137	3.612	2.20×10^{-13}
7596_V1ETCH	59	72,480	37	127	3864	68,452	0.054	0.226	5.177	1.00×10^{-12}
7556_L1CUCMP	14	67,155	34	121	3200	63,800	0.048	0.219	5.621	1.00×10^{-12}
6741_SAUSG	14	21,804	11	1	1153	20,639	0.053	0.917	174.161	1.00×10^{-12}
1358_STICMP	53	10,896	25	50	598	10,223	0.057	0.333	8.571	1.90×10^{-12}
7556_L1CUCMP	121	67,155	13	7	3221	63,914	0.048	0.65	36.459	3.80×10^{-12}

Continued on next page

Layer	Type of defect	Number of dice	Failure Defects	No failure Defects	Failure No defects	No failure No defects	Failure rate (all dice)	Failure rate (with defects)	Odds Ratio	p-value (adjusted)
7695_V2DEV	19	12,684	15	15	559	12,095	0.045	0.5	21.619	4.30×10^{-12}
1380_RTSAC	19	23,621	17	19	1214	22,371	0.052	0.472	16.498	4.30×10^{-12}
7550_L1ETCH	31	16,335	20	35	784	15,496	0.049	0.364	11.329	5.00×10^{-12}
7650_L2ETCH	19	23,556	16	17	1176	22,347	0.051	0.485	17.888	7.60×10^{-12}
1380_RTSAC	163	23,621	13	7	1218	22,383	0.052	0.65	33.756	9.10×10^{-12}
7676_L2CUCMP	27	23,595	25	47	1489	22,034	0.064	0.347	7.894	9.20×10^{-12}
7650_L2ETCH	59	23,556	26	73	1166	22,291	0.051	0.263	6.835	2.10×10^{-11}
1877_M255DEV	19	7268	13	9	362	6884	0.052	0.591	27.278	3.90×10^{-11}
5086_PETCH	69	27,180	10	3	1325	25,842	0.049	0.769	62.706	1.50×10^{-10}
7676_L2CUCMP	29	23,595	11	3	1503	22,078	0.064	0.786	51.878	1.70×10^{-10}
1045_M110DEV	19	9085	12	11	375	8687	0.043	0.522	25.208	2.00×10^{-10}
1278_AAETCH	11	5436	12	10	237	5177	0.046	0.545	26.087	2.10×10^{-10}
8368_L3CUCMP	14	32,778	12	8	1745	31,013	0.054	0.6	26.474	3.40×10^{-10}
7676_L2CUCMP	31	23,595	12	7	1502	22,074	0.064	0.632	24.947	1.10×10^{-9}
1684_RRII08	69	47,216	11	7	2567	44,631	0.055	0.611	27.091	2.00×10^{-9}
2187_M050DEV	164	56,327	12	11	2956	53,348	0.053	0.522	19.661	2.50×10^{-9}
2187_M050DEV	47	56,327	8	1	2960	53,358	0.053	0.889	128.286	3.40×10^{-9}
1380_RTSAC	63	23,621	9	3	1222	22,387	0.052	0.75	53.103	3.50×10^{-9}
7537_L1SVIL	1	3610	16	13	322	3259	0.094	0.552	12.409	3.80×10^{-9}
7708_V2ETCH	41	139,524	27	101	7281	132,115	0.052	0.211	4.872	4.00×10^{-9}
7556_L1CUCMP	64	67,155	11	10	3223	63,911	0.048	0.524	21.778	4.70×10^{-9}
5356_M610DEV	31	14,536	11	7	869	13,649	0.061	0.611	24.463	5.20×10^{-9}
7650_L2ETCH	62	23,556	8	2	1184	22,362	0.051	0.8	71.459	1.10×10^{-8}
7676_L2CUCMP	69	23,595	9	3	1505	22,078	0.064	0.75	42.527	2.00×10^{-8}
1684_RRII08	81	47,216	58	399	2520	44,239	0.055	0.127	2.558	2.10×10^{-8}
6502_M655DEV	19	9085	18	46	471	8550	0.054	0.281	7.138	2.50×10^{-8}
5086_PETCH	80	27,180	7	1	1328	25,844	0.049	0.875	121.481	3.00×10^{-8}
7596_V1ETCH	121	72,480	9	6	3892	68,573	0.054	0.6	26.194	8.20×10^{-8}
6270_M605DEV	31	39,974	14	28	2075	37,857	0.052	0.333	9.174	8.90×10^{-8}
1560_RTSDW	14	36,340	8	3	2069	34,260	0.057	0.727	42.768	9.00×10^{-8}
7295_CCBARR	32	58,080	15	35	2940	55,090	0.051	0.3	8.08	9.70×10^{-8}
1560_RTSDW	69	36,340	19	54	2058	34,209	0.057	0.26	5.881	1.10×10^{-7}
1380_RTSAC	64	23,621	7	2	1224	22,388	0.052	0.778	60.725	1.80×10^{-7}
7676_L2CUCMP	19	23,595	12	15	1502	22,066	0.064	0.444	11.781	1.90×10^{-7}
8368_L3CUCMP	10	32,778	7	2	1750	31,019	0.054	0.778	58.857	2.10×10^{-7}
8368_L3CUCMP	85	32,778	7	2	1750	31,019	0.054	0.778	58.857	2.10×10^{-7}
1684_RRII08	32	47,216	10	11	2568	44,627	0.055	0.476	15.819	2.50×10^{-7}
1684_RRII08	21	47,216	9	8	2569	44,630	0.055	0.529	19.497	3.60×10^{-7}
1293_STIOX16	81	3634	27	94	235	3278	0.072	0.223	4.02	3.60×10^{-7}
1544_DTIPCMP	82	47,112	28	166	2136	44,782	0.046	0.144	3.553	5.40×10^{-7}
1380_RTSAC	80	23,621	8	6	1223	22,384	0.052	0.571	24.234	6.10×10^{-7}
8368_L3CUCMP	69	32,778	9	10	1748	31,011	0.054	0.474	15.991	1.00×10^{-6}
6681_SALISTRIP	80	27,180	7	3	1578	25,592	0.058	0.7	36.752	1.10×10^{-6}
7550_L1ETCH	64	16,335	12	27	792	15,504	0.049	0.308	8.762	1.10×10^{-6}
5131_RTASDD	80	1817	6	3	67	1741	0.04	0.667	50.245	1.20×10^{-6}
6741_SAUSG	11	21,804	9	11	1155	20,629	0.053	0.45	14.655	1.60×10^{-6}
6741_SAUSG	81	21,804	37	241	1127	20,399	0.053	0.133	2.789	1.80×10^{-6}
7550_L1ETCH	42	16,335	10	18	794	15,513	0.049	0.357	10.927	2.20×10^{-6}
7650_L2ETCH	33	23,556	18	71	1174	22,293	0.051	0.202	4.846	2.20×10^{-6}

Continued on next page

Layer	Type of defect	Number of dice	Failure Defects	No failure Defects	Failure No defects	No failure No defects	Failure rate (all dice)	Failure rate (with defects)	Odds Ratio	p-value (adjusted)
8368_L3CUCMP	27	32,778	20	84	1737	30,937	0.054	0.192	4.267	3.10×10^{-6}
5086_PETCH	50	27,180	10	19	1325	25,826	0.049	0.345	10.335	3.20×10^{-6}
6741_SAUSG	83	21,804	27	151	1137	20,489	0.053	0.152	3.238	5.10×10^{-6}
1278_AAETCH	31	5436	9	17	240	5170	0.046	0.346	11.483	5.60×10^{-6}
7550_L1ETCH	62	16,335	5	1	799	15,530	0.049	0.833	87.395	7.00×10^{-6}
7650_L2ETCH	63	23,556	5	1	1187	22,363	0.051	0.833	84.736	8.00×10^{-6}
7596_V1ETCH	32	72,480	27	154	3874	68,425	0.054	0.149	3.112	8.90×10^{-6}
7596_V1ETCH	42	72,480	32	205	3869	68,374	0.054	0.135	2.771	9.80×10^{-6}
7550_L1ETCH	32	16,335	16	69	788	15,462	0.049	0.188	4.584	1.60×10^{-5}
7708_V2ETCH	121	139,524	8	13	7300	132,203	0.052	0.381	11.23	2.70×10^{-5}
1560_RTSDW	10	36,340	13	40	2064	34,223	0.057	0.245	5.434	3.10×10^{-5}
7708_V2ETCH	33	139,524	130	1531	7178	130,685	0.052	0.078	1.548	3.10×10^{-5}
8368_L3CUCMP	63	32,778	21	111	1736	30,910	0.054	0.159	3.39	3.70×10^{-5}
7556_L1CUCMP	19	67,155	11	35	3223	63,886	0.048	0.239	6.293	4.00×10^{-5}
5086_PETCH	164	27,180	5	3	1330	25,842	0.049	0.625	31.73	5.60×10^{-5}
7556_L1CUCMP	32	67,155	8	17	3226	63,904	0.048	0.32	9.42	6.20×10^{-5}
6270_M605DEV	11	39,974	35	272	2054	37,613	0.052	0.114	2.366	7.40×10^{-5}
7307_CCWCMP	14	3624	7	16	143	3458	0.041	0.304	10.691	1.00×10^{-4}
7550_L1ETCH	121	16,335	6	8	798	15,523	0.049	0.429	14.677	1.20×10^{-4}
1561_M265DEV	19	10,902	14	65	530	10,293	0.05	0.177	4.22	1.40×10^{-4}
6270_M605DEV	32	39,974	33	262	2056	37,623	0.052	0.112	2.315	1.80×10^{-4}
1380_RTSAC	21	23,621	11	40	1220	22,350	0.052	0.216	5.091	2.20×10^{-4}
1684_RRII08	15	47,216	9	25	2569	44,613	0.055	0.265	6.323	2.60×10^{-4}
7556_L1CUCMP	29	67,155	4	2	3230	63,919	0.048	0.667	38.178	2.70×10^{-4}
7676_L2CUCMP	28	23,595	4	1	1510	22,080	0.064	0.8	53.018	2.90×10^{-4}
2187_M050DEV	63	56,327	5	5	2963	53,354	0.053	0.5	18.004	3.00×10^{-4}
8368_L3CUCMP	121	32,778	5	5	1752	31,016	0.054	0.5	17.698	3.20×10^{-4}
7708_V2ETCH	42	139,524	37	318	7271	131,898	0.052	0.104	2.119	3.20×10^{-4}
7556_L1CUCMP	31	67,155	5	6	3229	63,915	0.048	0.455	16.578	3.40×10^{-4}
1560_RTSDW	64	36,340	4	2	2073	34,261	0.057	0.667	31.882	5.10×10^{-4}
6681_SALISTRIP	69	27,180	7	15	1578	25,580	0.058	0.318	7.656	6.70×10^{-4}
1877_M255DEV	11	7268	6	12	369	6881	0.052	0.333	9.439	7.20×10^{-4}
7676_L2CUCMP	121	23,595	4	2	1510	22,079	0.064	0.667	28.203	7.80×10^{-4}
2187_M050DEV	80	56,327	5	7	2963	53,352	0.053	0.417	12.974	8.20×10^{-4}
1684_RRII08	19	47,216	4	3	2574	44,635	0.055	0.571	22.833	9.20×10^{-4}
7596_V1ETCH	41	72,480	11	47	3890	68,532	0.054	0.19	4.17	9.30×10^{-4}
5356_M610DEV	56	14,536	7	16	873	13,640	0.061	0.304	6.921	0.001
7596_V1ETCH	84	72,480	6	14	3895	68,565	0.054	0.3	7.659	0.002
7708_V2ETCH	20	139,524	3	1	7305	132,215	0.052	0.75	49.84	0.002
7708_V2ETCH	25	139,524	3	1	7305	132,215	0.052	0.75	49.84	0.002
7556_L1CUCMP	27	67,155	23	198	3211	63,723	0.048	0.104	2.32	0.002
1560_RTSDW	63	36,340	3	1	2074	34,262	0.057	0.75	45.481	0.002
6270_M605DEV	69	39,974	5	10	2084	37,875	0.052	0.333	9.229	0.003
2187_M050DEV	11	56,327	26	219	2942	53,140	0.053	0.106	2.156	0.003
8368_L3CUCMP	19	32,778	4	5	1753	31,016	0.054	0.444	14.259	0.003
2187_M050DEV	69	56,327	7	23	2961	53,336	0.053	0.233	5.57	0.003
7307_CCWCMP	19	3624	7	32	143	3442	0.041	0.179	5.351	0.003
1278_AAETCH	64	5436	5	13	244	5174	0.046	0.278	8.305	0.003
2187_M050DEV	14	56,327	6	18	2962	53,341	0.053	0.25	6.109	0.004

Continued on next page

Table S6 – *Continued from previous page*

Layer	Type of defect	Number of dice	Failure Defects	No failure Defects	Failure No defects	No failure No defects	Failure rate (all dice)	Failure rate (with defects)	Odds Ratio	p-value (adjusted)
1684_RRII08	164	47,216	3	2	2575	44,636	0.055	0.6	25.392	0.005
6681_SALISTRIP	11	27,180	13	72	1572	25,523	0.058	0.153	2.961	0.005
1358_STICMP	63	10,896	3	2	620	10,271	0.057	0.6	24.251	0.005
8368_L3CUCMP	80	32,778	5	12	1752	31,009	0.054	0.294	7.51	0.005
7650_L2ETCH	159	23,556	5	13	1187	22,351	0.051	0.278	7.382	0.005
7596_V1ETCH	10	72,480	6	19	3895	68,560	0.054	0.24	5.66	0.006
1560_RTSDW	11	36,340	29	252	2048	34,011	0.057	0.103	1.921	0.007
8368_L3CUCMP	28	32,778	3	3	1754	31,018	0.054	0.5	17.679	0.008
8368_L3CUCMP	62	32,778	4	8	1753	31,013	0.054	0.333	9.016	0.009
7295_CCBARR	46	58,080	5	15	2950	55,110	0.051	0.25	6.359	0.009
7556_L1CUCMP	28	67,155	3	4	3231	63,917	0.048	0.429	15.019	0.01
7676_L2CUCMP	64	23,595	4	7	1510	22,074	0.064	0.364	8.49	0.012
1278_AAETCH	42	5436	5	20	244	5167	0.046	0.2	5.415	0.015
5086_PETCH	19	27,180	3	5	1332	25,840	0.049	0.375	11.871	0.016
1560_RTSDW	80	36,340	6	23	2071	34,240	0.057	0.207	4.398	0.017
6681_SALISTRIP	14	27,180	8	39	1577	25,556	0.058	0.17	3.377	0.018
2187_M050DEV	121	56,327	3	5	2965	53,354	0.053	0.375	11.013	0.019
2187_M050DEV	64	56,327	3	5	2965	53,354	0.053	0.375	11.013	0.019
2187_M050DEV	56	56,327	29	298	2939	53,061	0.053	0.089	1.766	0.019
1877_M255DEV	15	7268	2	1	373	6892	0.052	0.667	34.672	0.02
7596_V1ETCH	20	72,480	2	1	3899	68,578	0.054	0.667	33.051	0.022
1684_RRII08	121	47,216	2	1	2576	44,637	0.055	0.667	32.559	0.022
1684_RRII08	64	47,216	2	1	2576	44,637	0.055	0.667	32.559	0.022
1544_DTIPCMP	42	47,112	9	65	2155	44,883	0.046	0.122	2.929	0.022
7708_V2ETCH	32	139,524	35	388	7273	131,828	0.052	0.083	1.642	0.022
1560_RTSDW	162	36,340	2	1	2075	34,262	0.057	0.667	31.023	0.024
1544_DTIPCMP	46	47,112	44	604	2120	44,344	0.046	0.068	1.529	0.028
1380_RTSAC	42	23,621	5	21	1226	22,369	0.052	0.192	4.449	0.03
7650_L2ETCH	43	23,556	7	41	1185	22,323	0.051	0.146	3.278	0.031
6681_SALISTRIP	77	27,180	3	6	1582	25,589	0.058	0.333	8.291	0.034
7650_L2ETCH	64	23,556	2	2	1190	22,362	0.051	0.5	18.784	0.036
1560_RTSDW	32	36,340	2	2	2075	34,261	0.057	0.5	16.508	0.046
1544_DTIPCMP	11	47,112	8	62	2156	44,886	0.046	0.114	2.734	0.047

Table S6: Odds Ratio analysis with respect to layers and types of defect for the dataset ARES. For each layer and type of defect, the table reports the number of inspected dice along with the corresponding contingency table. Additionally, the failure rate is provided for all dice inspected at each layer, both for all dice and those with defects found. The Odds Ratio value, along with the adjusted p-value for the test $OR = 1$, corrected for multiplicity, is presented. Entries are sorted in descending order of p-values. The table is based on the entire dataset (dice inspected for generic defects of type = 0).

S2 Layers of dice inspected for specific defects

This section presents the structure of layers inspected for specific (type > 0) or generic (type $= 0$) defects in a graphical format, featuring a main matrix plot and two histograms on the right and top.

S2.1 Dataset TETIS

Figures S4 and S5 represent the matrix plot of the layer structure for the dataset TETIS for dice inspected by SEM and the entire dataset, respectively. Each row of the matrix represents a specific set of layers subject to inspection. For instance, the bottom row of Fig. S4 includes a single layer, 1192_ETHM583, depicted in brown color in the first cell of the row. The top row of the matrix for the same dataset encompasses 4 layers, represented with brown colors in the cells corresponding to the inspected layers. Rows are sorted by the number of inspected layers, with the lowest (1) at the bottom and the highest (4) at the top. Lot-wafers with the same total number of inspected layers but different layers are alphabetically sorted based on the name of the first inspected layer. If we fix the number of inspected layers, different sets of layers at which dice are actually inspected exist, and they are visually clustered together: each block corresponds to a fixed number of inspected layers, with dashed lines separating blocks.

One or more lot-wafers may have been inspected at a particular set of inspected layers. This information is represented in the histogram on the right y-axis. For example, the histogram bin at the right of the first bottom row indicates that dice were inspected at the unique layer 1192_ETHM583 on 7 lot-wafers. The highest number of lot-wafers (32) corresponds to the layers 6741_SAUSG and 8024_V3RDLBARR of inspection. The figure visually shows that the selection of layers to investigate is not random. The top x-axis histogram displays the total number of lot-wafers inspected at the layers indicated on the x-axis.

The structure significantly differs from that of all dice. Notably, the number of layers inspected per wafer is considerably smaller. The histogram on the right y-axis, representing the frequency of lot-wafers inspected, is predominantly concentrated on block 1 (indicated on the left y-axis), corresponding to only one layer inspected per wafer. Furthermore, the maximum number of layers inspected per lot-wafer is 4, much lower than the 13 layers observed in the entire dataset. Simultaneously, there is a noticeable decrease in the significance of layer 1684_RRII08, which was the most frequent in the entire dataset.

S2.2 Dataset ARES

We examine the analogous structure of inspected layers for the datasets ARES with type $= 0$ (Fig. S6). Remarkable differences are noticeable compared to the structure of the entire dataset (dice inspected for generic defects, type $= 0$, Fig. 2 of the parent paper). Notably, the role of the same layer 1684_RRII08, which was prominent in the dataset for generic defects, is now much less significant. Moreover, the maximum number of inspected layers for a lot-wafer is 6, which is one third of the case observed in the entire dataset.

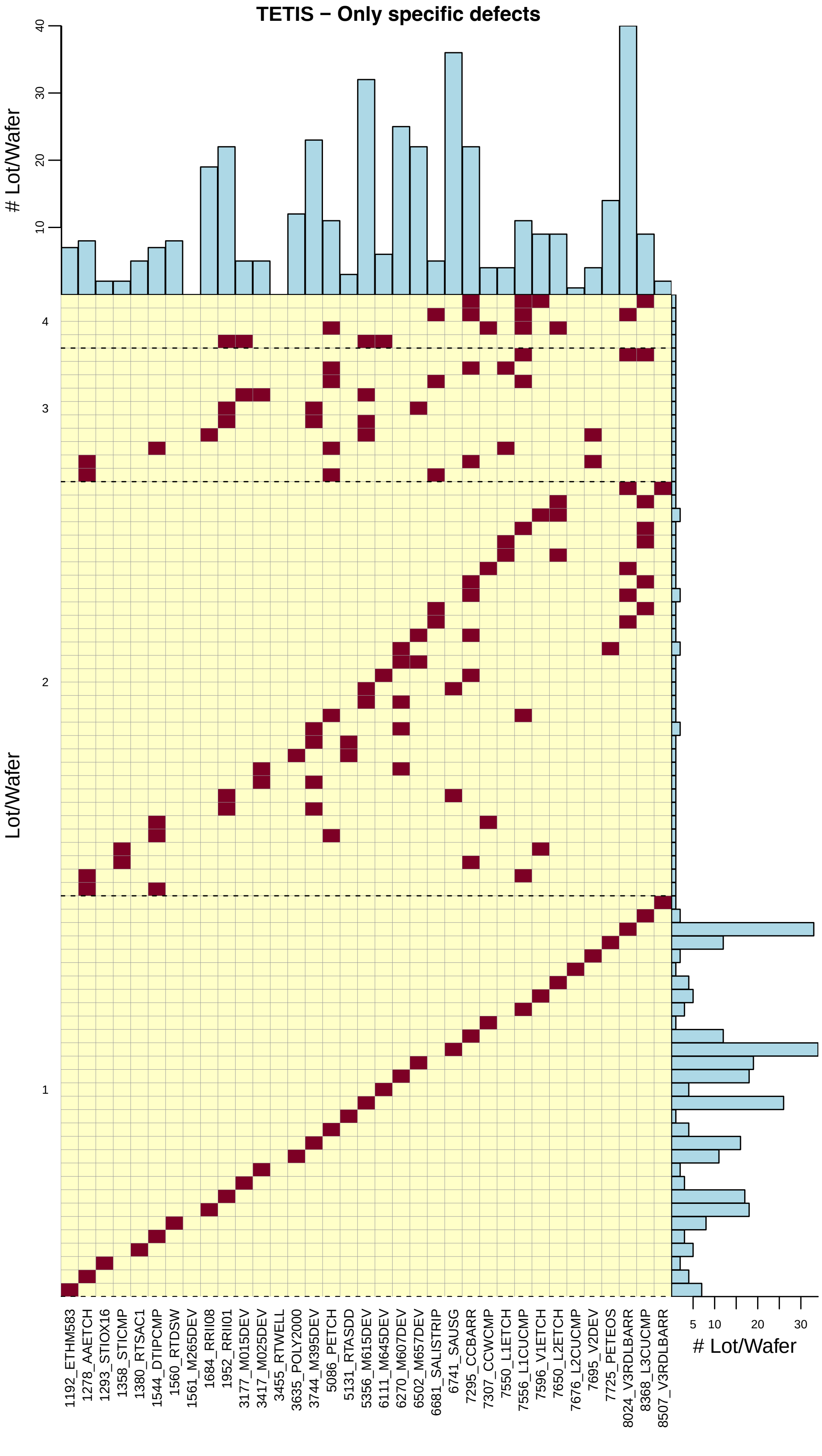

Figure S4: Structure of inspected layers (only specific defects, type > 0) for the dataset TETIS.

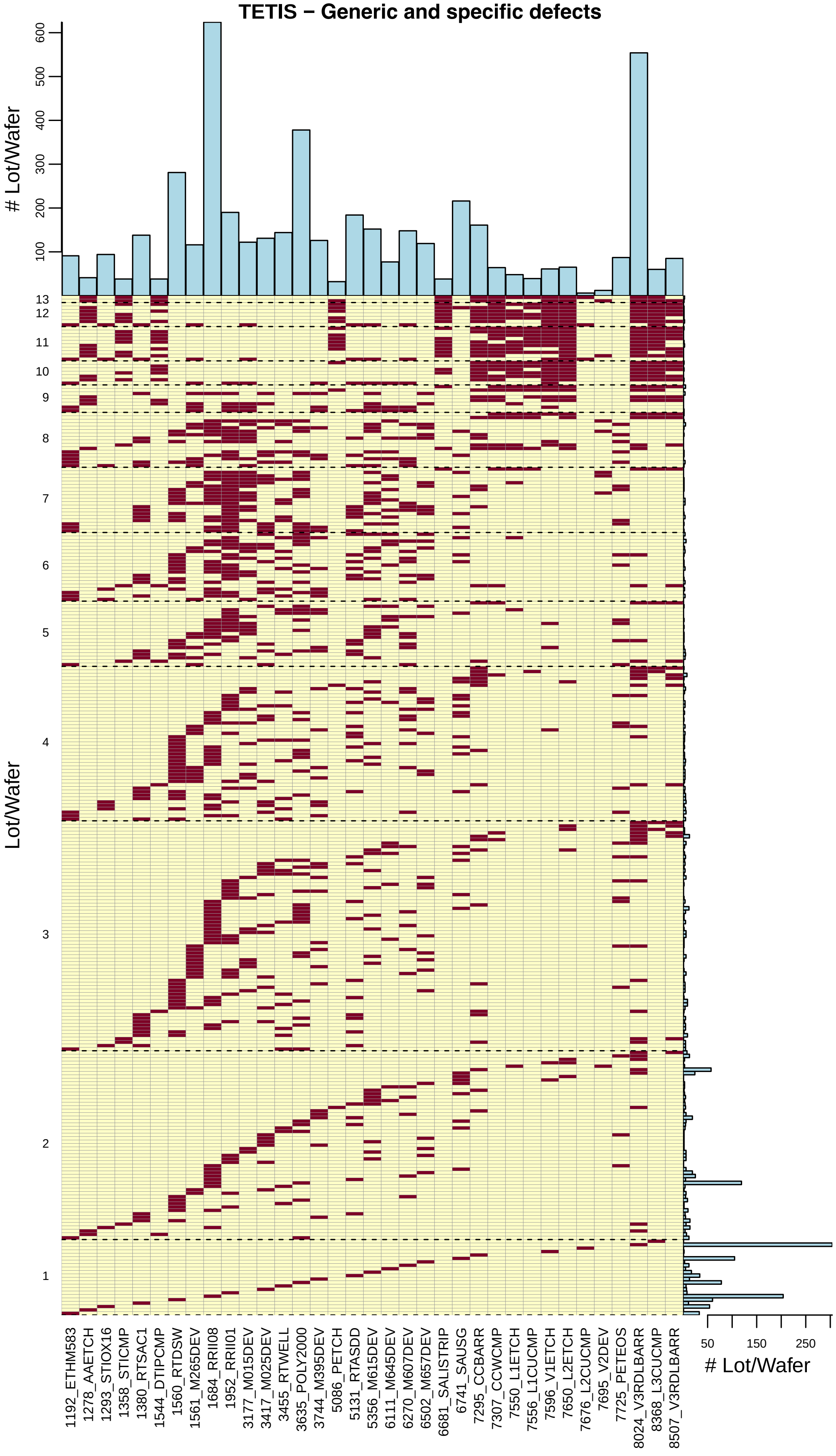

Figure S5: Structure of inspected layers for the dataset TETIS, considering dice for which at least one defect of any type (including generic defects of type = 0) is detected.

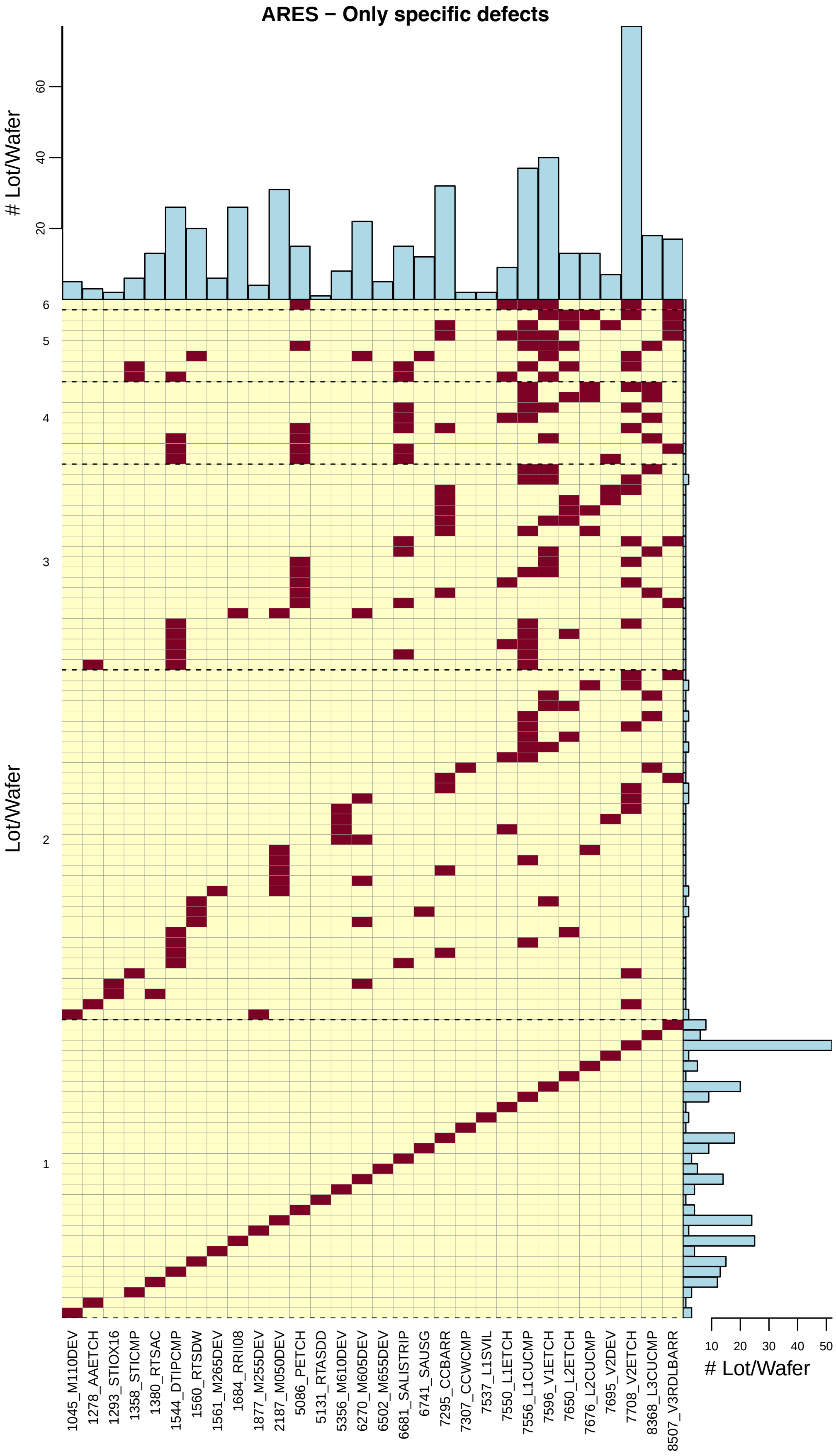

Figure S6: Structure of inspected layers (only specific defects, type > 0) for the dataset ARES.

S3 Prediction model

This Section contains materials on the prediction models not included in the parent paper for brevity's sake.

S3.1 Dataset TETIS

S3.1.1 Predictor variables

Fig. S7 depicts the average failure rate of dice per wafer slot.

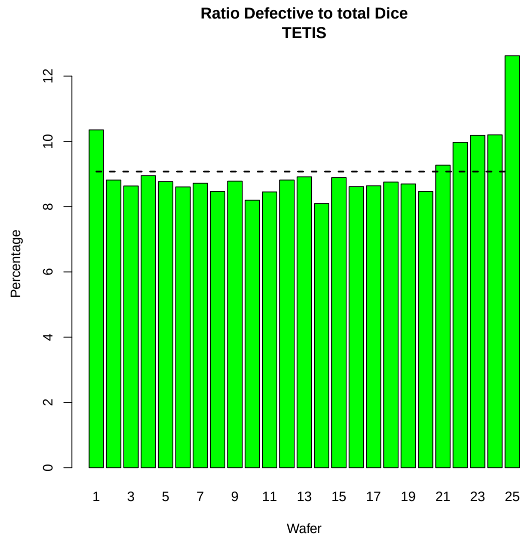

Figure S7: Bar chart illustrating the average rate of electrically failed dice by wafer slot for the dataset TETIS. The dashed horizontal line indicates the global average rate of electrical failures for the dice.

The failure rate is not uniform across wafer slots, with slot 25 and, to a lesser extent, 1 showing a higher rate of failure. This observation is supported quantitatively by an ANOVA analysis on the failure rate for each wafer slot, yielding a p-value numerically close to 0. Subsequent post-hoc analysis using Tukey Honest Significant Differences (HSD) with multiplicity correction reveals that the average failure rate for wafer slot 25 is significantly different from all other slots, and wafer slot 1 is significantly different from the other slots. No other significant differences are observed among the remaining wafers slots. In practice, we can cluster the wafer slots into three different behaviors: wafer slot 1, 25, and all the other ones. This observation has been confirmed through interaction with STMicroelectronics engineers, particularly regarding wafer slot 25.

Figure S8 (left) illustrates the spatial distribution of the average rate of electrical failures for the dice. We observe that the majority of failures are concentrated at the boundary of the wafer, reaching high values that exceed 70%. To better visualize the global effect, Fig. S8 (right) presents a scatter plot of the rate of electrical failures against the distance of the die from the center of the wafer (normalized to 1). The impact of failures on the boundary is highly evident. In other regions of the wafer, the average rate of failure remains relatively constant up to a (normalized) distance of about 0.80 (depending on the dataset). Beyond this point, it gradually increases before steeply rising at the boundary.

S3.1.2 Prediction model by die–case without interaction

Figs. S9–S12 and Tab. S7 present results similar to those obtained for the dataset ARES (cf. Figs. 8–11 and Tab. 6 of the parent paper).

The optimal number of iterations for controlling overfitting is 144, which is lower than the dataset ARES, and the AUC is 0.66, slightly worse than the dataset ARES. Both indicate a higher level of noise in the dataset and a less accurate fit of the regression. Once again, each accuracy indicator has its own optimal cutoff, and their range is quite wide (from 0.08 to 0.51, see Tab. S7). Interestingly, optimal cutoffs are very similar between the datasets ARES and TETIS (compare with Tab. 6 of the parent paper).

As in the dataset ARES, distance is by far the most important feature, and slots 1 and 25 play the most critical roles among wafers. Four out of the five most important types of defects are ranked first in the list of the most

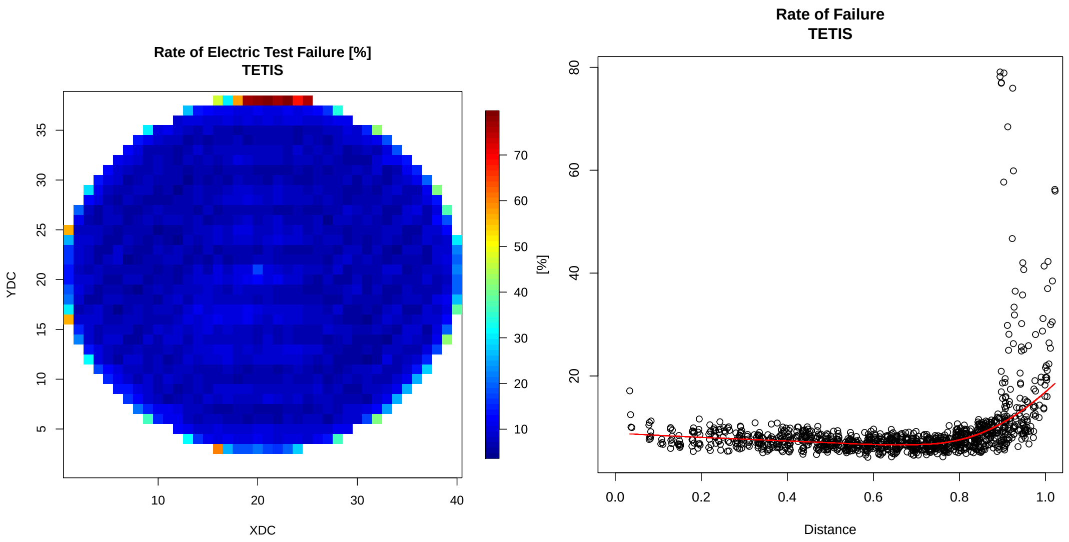

Figure S8: Spatial distribution of electrical failures on the wafer (left). Plot of the rate of electrical failures of dice vs. the distance from the center of the wafer, normalized to the range $[0, 1]$ (right). The red line represents LOWESS nonparametric smoothing. Plots refer to dataset TETIS.

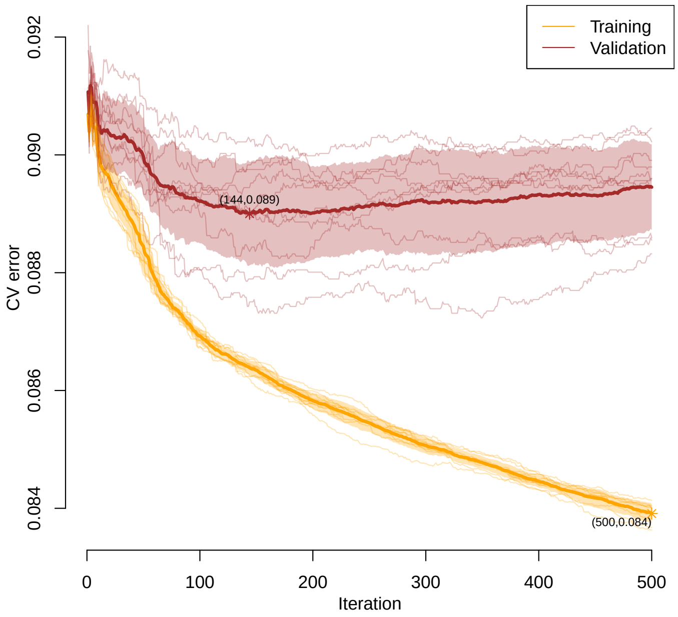

Figure S9: Error curve of the training and validation subsets in 10-fold cross-validation for the XGB model with logistic loss and Accuracy as cost and evaluation function, respectively, on the subset of TETIS containing only dice inspected for defects of type > 0. Solid thick lines represent the average value over the 10 folds, shaded areas correspond to one standard deviation, and thin lines depict the 10-fold cross-validation curves.

significant defects according to the Odds Ratio analysis (Fig. S3), In addition, five out of the seven most important layers are ranked first among the most significant ones from the Odds Ratio analysis (Fig. S2).

S3.1.3 Prediction model by die–case with interaction

This Section shows the results of the prediction model with interaction between layers and types of defects for the dataset TETIS. The total number of variables of the model is 2,575 with 368,148 dice. They include distance, layers, types of defects, wafers and couples layers-types of defects. However, we recall that only 18,699 show at least one specific defect (5.1%) and only 34,021 dice underwent an electric failure (9.2%). This is surely the most

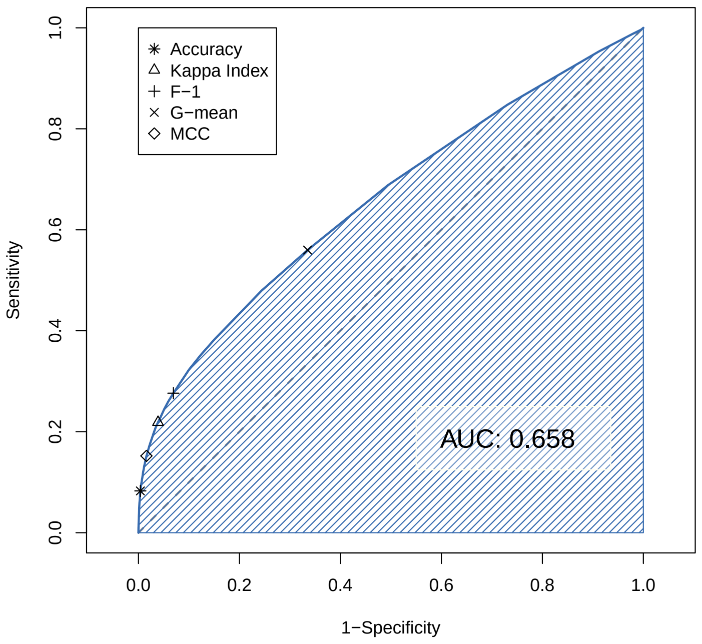

Figure S10: ROC curve for the XGB model with logistic loss and Accuracy as cost and evaluation function, respectively, on the subset of TETIS containing only dice inspected for defects of type > 0. Optimal values of the indicators are reported on the curve according to the legend.

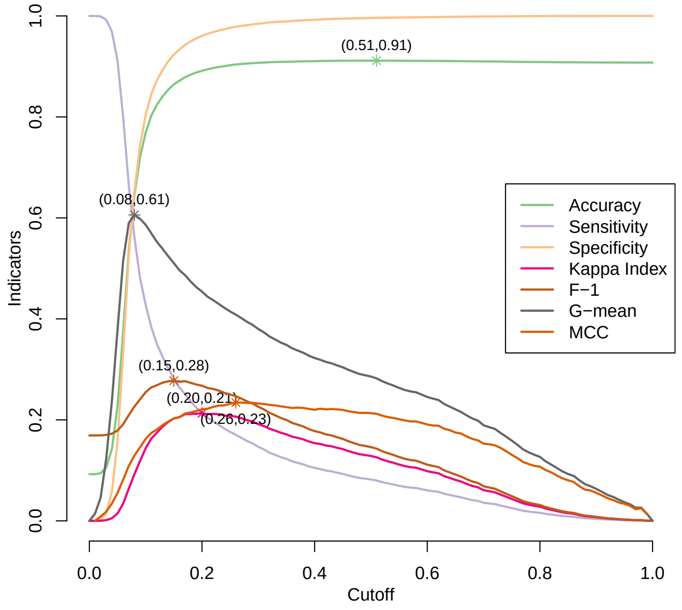

Figure S11: Indicators of accuracy vs. cutoff for the XGB model with logistic loss and Accuracy as cost and evaluation function, respectively, on the subset of TETIS including only dice inspected for defect of type > 0. Optimal values are marked with an asterisk along with the corresponding values of the cutoff and the indicator.

interesting subset of data, because it directly links defects detected and electric failures for finding eventual causal relationships.

Results are shown in Fig. S13, where the most important 50 variables are graphically shown sorted according to the indicator discussed in Section 6.2 of the parent paper. They are well consistent with analysis of the noninteractive predictive model for layers, types of defects and wafers (compare Fig. S13 with Fig. S12). We again see that distance is by far the most predictive variable. If we analyze interaction between layers and types of defects, we see that very few of them are included among the most important ones, and they involve the type of defect 11.

Indicator	Cutoff	Sensitivity	Specificity	Accuracy	κ coefficient	F-1	G-mean	MCC
Accuracy	0.51	0.080	0.996	0.911	0.126	0.143	0.282	0.212
κ coefficient	0.20	0.215	0.960	0.891	0.213	0.267	0.454	0.221
F-1	0.15	0.282	0.924	0.864	0.203	0.278	0.510	0.203
G-mean	0.08	0.562	0.654	0.645	0.092	0.226	0.606	0.129
MCC	0.26	0.170	0.979	0.904	0.206	0.247	0.408	0.234

Table S7: Best cutoff for each accuracy indicator (rows) and their corresponding values for all best cutoffs (columns). The data pertain to the XGB model with logistic loss and Accuracy as cost and evaluation functions, respectively, applied to the subset of TETIS including only dice inspected for defects of type > 0.

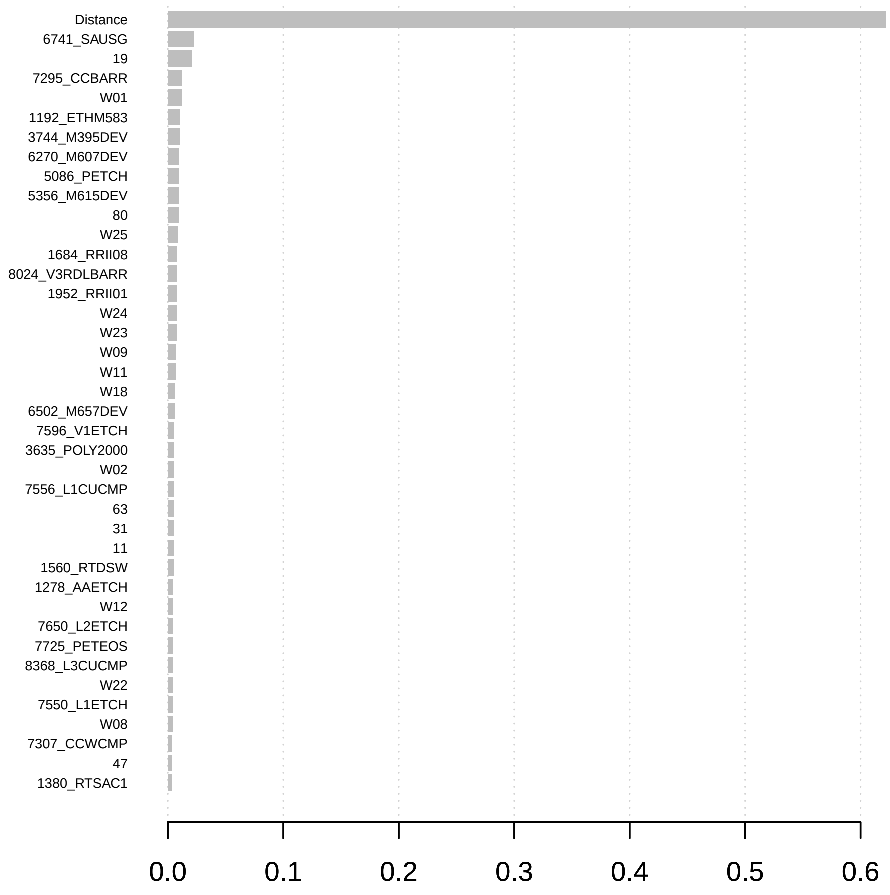

Figure S12: Importance of predictors in the XGB model with logistic loss and Accuracy as cost and evaluation functions, respectively, applied to the subset of TETIS that includes only dice inspected for defects of type > 0.

S3.1.4 Prediction model–Yield of a wafer

Results of the prediction of yield of a wafer are reported here for the dataset TETIS analogously to Section 6.4 of the parent paper.

A summary is reported in Tab. S8.

Fig. S14 reports the scatter plot of the wafer rate of failure without interaction between layers and types of defects.

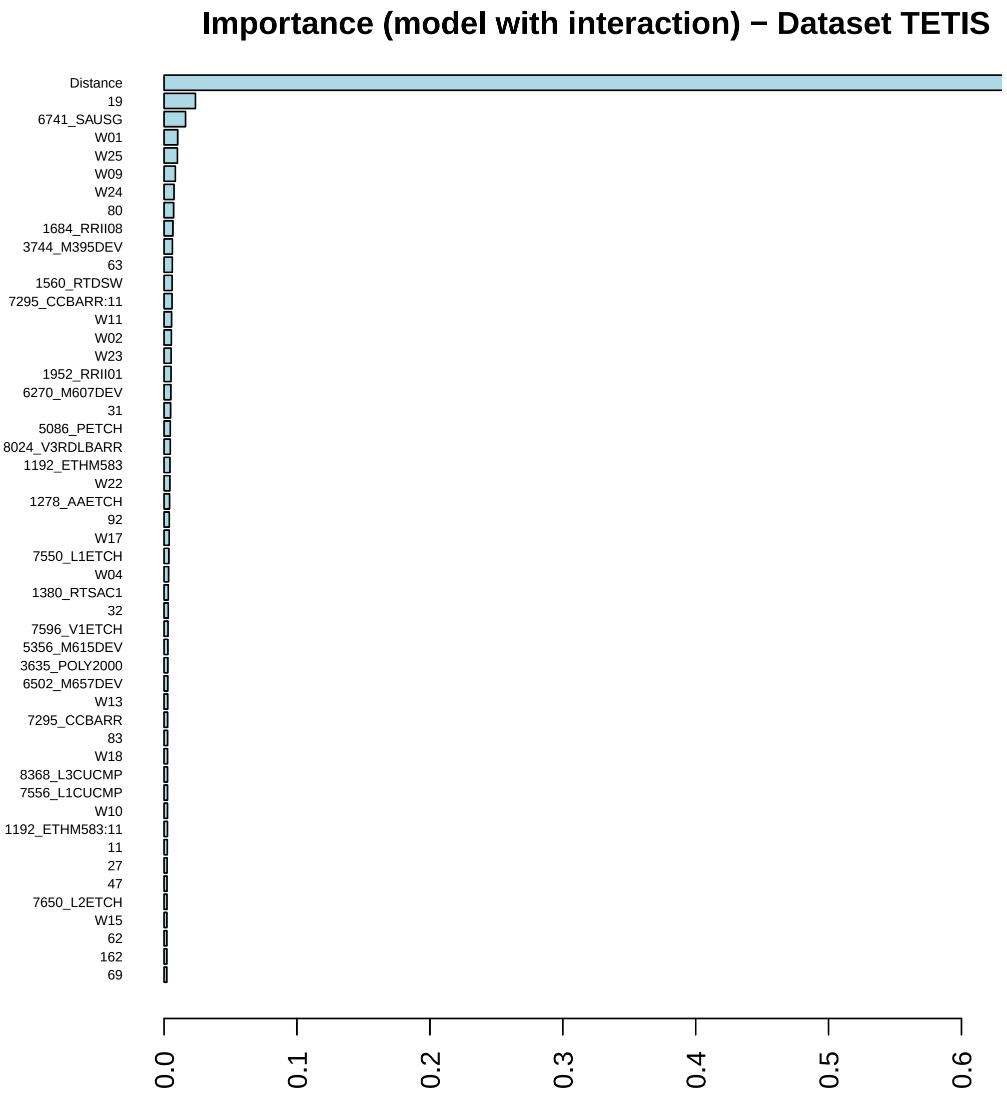

Figure S13: Importance of predictors in the XGB model with logistic loss and Accuracy as cost and evaluation functions, respectively, applied to the subset of TETIS that includes only dice inspected for defects of type > 0. The prediction model includes interaction between layers and types of defects. Layers are indicated by their name and types of defects by their code. Names of couples layers-types of defects are separated by a column (:).

Model	Dataset ARES		Dataset TETIS	
	RMSE	MAE	RMSE	MAE
Rate of failure	0.053		0.092	
Without interaction	0.023	0.018	0.056	0.045
With interaction	0.024	0.018	0.056	0.045
Reference	0.019	0.014	0.033	0.025

Table S8: Accuracy in estimating the yield of a wafer starting from the prediction model die-by-die for the datasets ARES (second and third column) and TETIS (fourth and fifth column). The second and third row show the RMSE and MAE of the models without and with interaction between layers and types of defects. As a reference, the last row indicates the RMSE and the MAE when prediction is the average rate of failure as reported in the first row.

S3.2 Dataset ARES

S3.3 Prediction model–Yield of a wafer

We report in Fig. S15 the scatter plot of the wafer rate of failure with interaction between layers and types of defects.

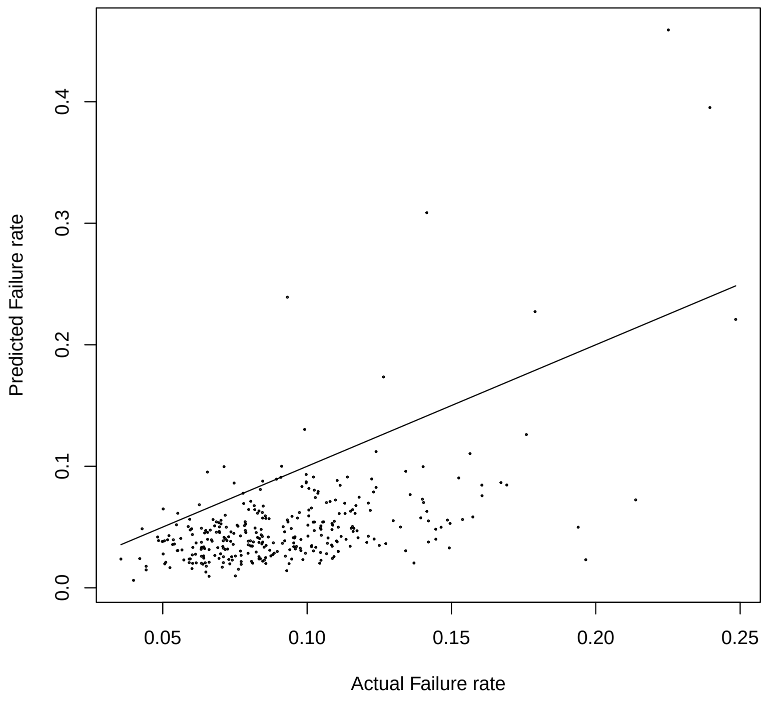

Figure S14: Scatter plot of predicted rate of failure vs. actual one at wafer level. Estimations are obtained aggregating results of the classification/regression model without interaction of layers and types of defects. Results refer to the dataset TETIS.

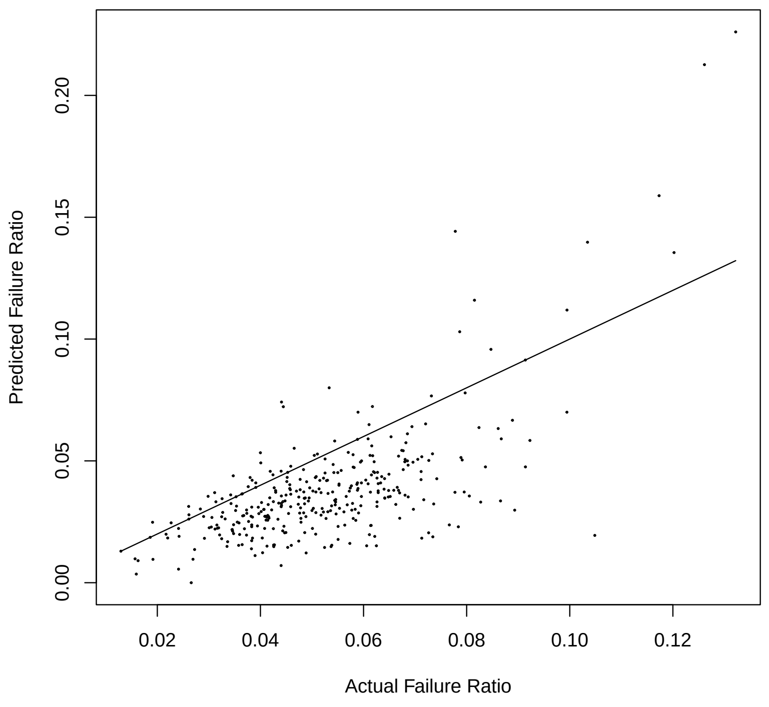

Figure S15: Scatter plot of predicted rate of failure vs. actual one at wafer level. Estimations are obtained aggregating results of the classification/regression model without interaction of layers and types of defects. Results refer to the dataset ARES.
